# Communicating the Imperfect Diagnostic Accuracy of COVID-19 Rapid Antigen Self-Tests: An Online Randomized Experiment

**DOI:** 10.1177/0272989X241242131

**Published:** 2024-04-23

**Authors:** Huijun Li, Megha Kalra, Lin Zhu, Deonna M. Ackermann, Melody Taba, Carissa Bonner, Katy J.L. Bell

**Affiliations:** Sydney School of Public Health, Faculty of Medicine and Health, University of Sydney, Sydney, NSW, Australia; Sydney School of Public Health, Faculty of Medicine and Health, University of Sydney, Sydney, NSW, Australia; Sydney School of Public Health, Faculty of Medicine and Health, University of Sydney, Sydney, NSW, Australia; Sydney School of Public Health, Faculty of Medicine and Health, University of Sydney, Sydney, NSW, Australia; Sydney School of Public Health, Faculty of Medicine and Health, University of Sydney, Sydney, NSW, Australia; Sydney School of Public Health, Faculty of Medicine and Health, University of Sydney, Sydney, NSW, Australia; Sydney School of Public Health, Faculty of Medicine and Health, University of Sydney, Sydney, NSW, Australia

**Keywords:** diagnostic accuracy, risk communication, COVID-19, rapid antigen tests, consumer self tests

## Abstract

**Objective:**

To investigate the potential impacts of optimizing coronavirus disease 2019 (COVID-19) rapid antigen test (RAT) self-testing diagnostic accuracy information.

**Design:**

Online randomized experiment using hypothetical scenarios: in scenarios 1 to 3 (RAT result positive), the posttest probability was considered to be very high (likely true positives), and in scenarios 4 and 5 (RAT result negative), the posttest probability was considered to be moderately high (likely false negatives).

**Setting:**

December 12 to 22, 2022, during the mixed-variant Omicron wave in Australia.

**Participants:**

Australian adults. Intervention: diagnostic accuracy of a COVID-19 self-RAT presented in a health literacy-sensitive way; usual care: diagnostic accuracy information provided by the manufacturer; control: no diagnostic accuracy information.

**Main Outcome Measure:**

Intention to self-isolate.

**Results:**

A total of 226 participants were randomized (control *n* = 75, usual care *n* = 76, intervention *n* = 75). More participants in the intervention group correctly interpreted the meaning of the diagnostic accuracy information (*P* = 0.08 for understanding sensitivity, *P* < 0.001 for understanding specificity). The proportion who would self-isolate was similar across scenarios 1 to 3 (likely true positives). The proportion was higher in the intervention group than in the control for scenarios 4 and 5 (likely false negatives). These differences were not statistically significant. The largest potential effect was seen in scenario 5 (dinner party with confirmed cases, the person has symptoms, negative self-RAT result), with 63% of the intervention group and 49% of the control group indicating they would self-isolate (absolute difference 13.3%, 95% confidence interval: −2% to 30%, *P* = 0.10).

**Conclusion:**

Health literacy sensitive formatting supported participant understanding and recall of diagnostic accuracy information. This may increase community intentions to self-isolate when there is a likely false-negative self-RAT result. Trial registration: Australia New Zealand Clinical Trial Registry (ACTRN12622001517763)

**Highlights:**

- Community-based diagnostic accuracy studies of COVID-19 self-RATs indicate substantially lower sensitivity (and higher risk of false-negative results) than the manufacturer-supplied information on most government public Web sites.
- This online randomized study found that a health literacy–sensitive presentation of the imperfect diagnostic accuracy COVID-19 self-RATs supported participant understanding and recall of diagnostic accuracy information.
- Health literacy–sensitive presentation may increase community intentions to self-isolate after a negative test result where the posttest probability is still moderately high (i.e., likely false-negative result).
- To prevent the onward spread of infection, efforts to improve communication about the high risk of false-negative results from COVID-19 self-RATs are urgently needed.

Although the pandemic has been officially declared as over,^1^ the SARS-CoV-2 virus continues to spread in communities, causing morbidity and mortality from COVID-19.^2^ A key strategy to prevent onward spread is testing to facilitate detection and isolation of SARS-CoV-2–infected people until they are no longer infectious.^3,4^ The use of reverse-transcription polymerase chain reaction (RT-PCR) tests was previously the mainstay for identifying COVID-19–positive cases. However, access to RT-PCR tests is now more limited and requires a referral from a general practitioner or nurse practitioner. Self-testing using a rapid antigen test (RAT) is now the mainstay method of identifying COVID-19 infection in Australia.^5^

RATs offer several advantages over RT-PCR tests, including allowing self-testing at home with results available within 15 to 20 min^6^ and high specificity for infectiousness.^7^ The use of RATs for self-testing was first approved by the Australian Therapeutic Goods Administration in November 2021.^8^ The World Health Organization recommends that RATs have a minimum sensitivity of 80% and a minimum specificity of 97% among symptomatic individuals.^9^ However, when RATs are used in a real-world setting, their clinical sensitivity often fails to meet this minimum threshold,^6^ especially when used as a self-test by consumers.^10^ Despite findings of research studies of suboptimal sensitivity, the publicly available information on the accuracy of RATs in use in Australia is that provided by test manufacturers.^8^ This is likely to lead consumers to believe the tests have very high sensitivity and can be relied on for ruling out COVID-19 infection.

Overestimation of the sensitivities of RATs may lead to underappreciation of the chance of false-negative test results, resulting in avoidable onward transmission of SARS-CoV-2 and new cases of COVID-19. The understanding and interpretation of test results may improve if information provided is based on decision science principles,^11^ is health literacy sensitive,^12^ and includes graphics such as pictograms or photographs.^13^

We aimed to investigate the potential impacts of providing diagnostic accuracy information that is more applicable to consumers undertaking self-testing^14^ and presented in a health literacy–sensitive way, compared with the accuracy information currently provided to consumers.

## Methods

A summary of the methods is provided below. and a more detailed description is provided in the prespecified study protocol.^15^

### Setting and COVID-19 Context

The study was conducted from December 12 to 22, 2022, during the Omicron wave in Australia, when there was an average of 15,937 new COVID-19 cases per day nationwide.^16^ At this time, there was no longer mandatory reporting of positive RATs nor isolation of COVID-19–positive cases in Australia, both requirements having ceased in October 2022. Although health policies continued to encourage these behaviors, the responsibility to do these and other preventative behaviors was left up to the individual.

### Study Population

Participants were recruited from the general Australian public through an independent social research company (Dynata), which has a panel of 600,000 participants whose demographic characteristics align closely with those of the national population. Dynata has a points system in which participants receive points after completing surveys. The points can then be used to redeem vouchers, cash, or other rewards. Participants were eligible if they were at least 18 y of age, able to read and understand English, and resided in Australia. Stratified sampling was used, with quotas in place for gender (50% male, 50% female or other), age (50% younger than 40 y, 50% 40 y or older), and education (50% no university degree, 50% university degree).

### Design

Participants were randomly assigned to 1 of 3 study arms: intervention arm, diagnostic accuracy information from a community-based study of self-testing provided in a way that was health literacy sensitive; usual care arm, manufacturer supplied diagnostic information provided; or control arm, no diagnostic accuracy information provided (allocation ratio, 1:1:1).

### Interventions

#### Hypothetical scenarios

Participants in each of the 3 randomized groups were asked to read through the materials for the condition they were allocated to and to imagine they were in 5 different hypothetical scenarios that were presented sequentially. In all scenarios, there was a high probability that the individual was infectious with COVID-19, but the RAT results differed across the 5 scenarios. In scenarios 1 to 3, the RAT result was positive, and posttest probability was considered to be very high (likely true positives). In scenarios 4 and 5, the RAT result was negative, but the posttest probability was still considered to be moderately high (likely false negatives; Box 1).

**Box 1:** The 5 Hypothetical Scenarios in the Survey^a^

**Scenario 1 (very high posttest probability)**: Imagine you have been unwell with symptoms including headache, sore throat, fever, runny nose, and loss of taste and smell.	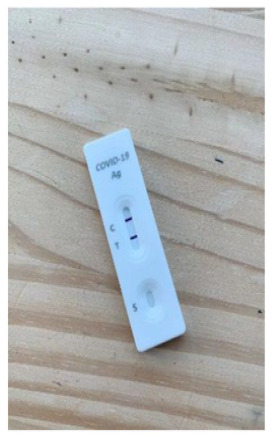	**Scenario 3 (high posttest probability):** It is now day 6 since you first developed symptoms. You have felt well since day 4, with no symptoms.	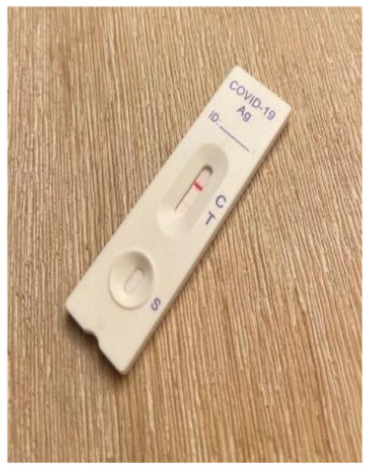	**Scenario 5 (moderately high posttest probability):** Imagine it is now 6 months later and you have dinner at your friend’s house with 9 other people. The dinner lasted about 3 hours. Two days later your friend told you that they and 2 other people at the dinner party have tested positive for COVID-19. You are experiencing a sore throat and runny nose.	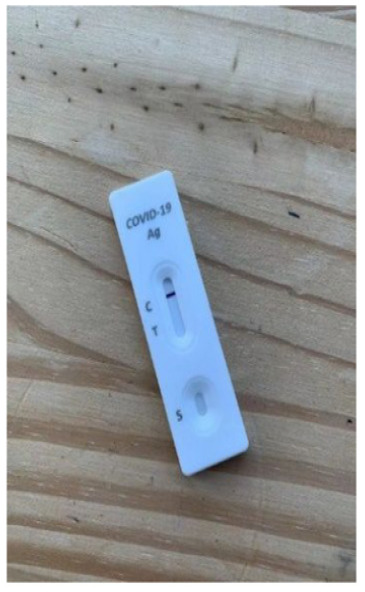
**Scenario 2 (very high posttest probability):** You have COVID-19 and have been isolating at home. It is now day 4 since you first developed symptoms, and you have felt well since waking up today and no longer have symptoms.	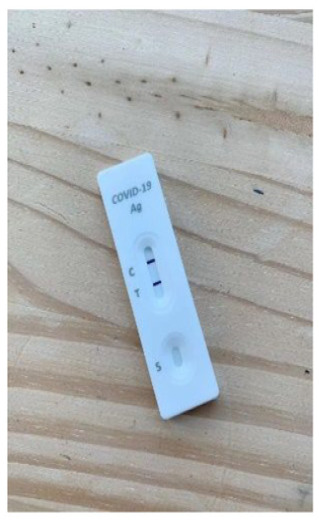	**Scenario 4 (moderately high posttest probability):** Now imagine someone was staying at your home while you were sick with COVID-19. On day 7 since you first developed symptoms, they wake up with a sore throat and runny nose.	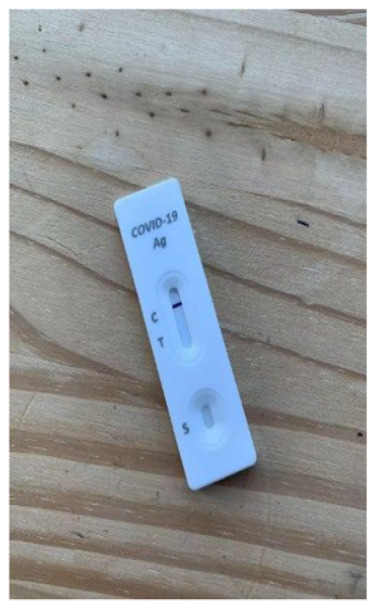		

a Posttest probabilities were calculated using the methods outlined in an article presenting Fagan’s nomogram for coronavirus disease 19 (COVID-19).^17^ We assumed a high pretest probability across all 5 scenarios, consistent with the information provided and the context of high community transmission in the Australian community at the time of the study (December 2022). Using the sensitivity of 52% and specificity of 99% found in the community-based study of the Flowflex RAT (positive likelihood ratio 52, negative likelihood ratio 0.48) and assuming a pretest probability of 50%, we calculated that posttest probabilities were >80% across scenarios 1 to 3 with a positive RAT result, which we considered to be very high. Assuming a pretest probability of 50% in scenarios 4 and 5 with a negative RAT result, we calculated posttest probabilities of 25%, which we considered moderately high (i.e., probability is still well above suggested management decision thresholds to self-isolate).^17^

#### Diagnostic accuracy information

We used diagnostic accuracy information for the Flowflex RAT. This test is approved for use in Australia, and diagnostic accuracy information was available from a large independent community-based study of self-testing^14^ as well as from the manufacturer.^8,18^

**Figure 1:**
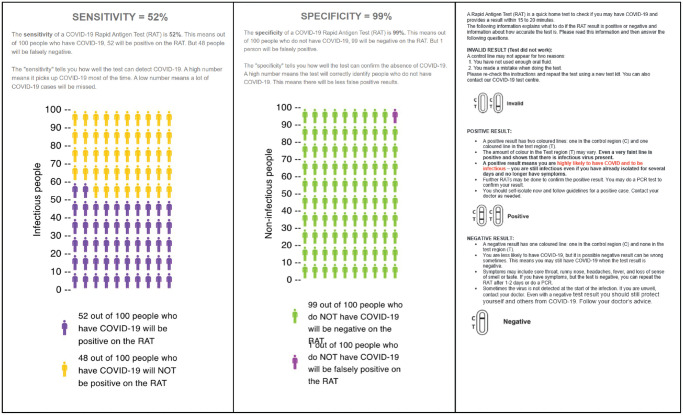
Diagnostic accuracy information for intervention. Graphical presentation of clinical diagnostic accuracy estimates from a population-based study of the Flowflex RAT.^14^

**Figure 2:**
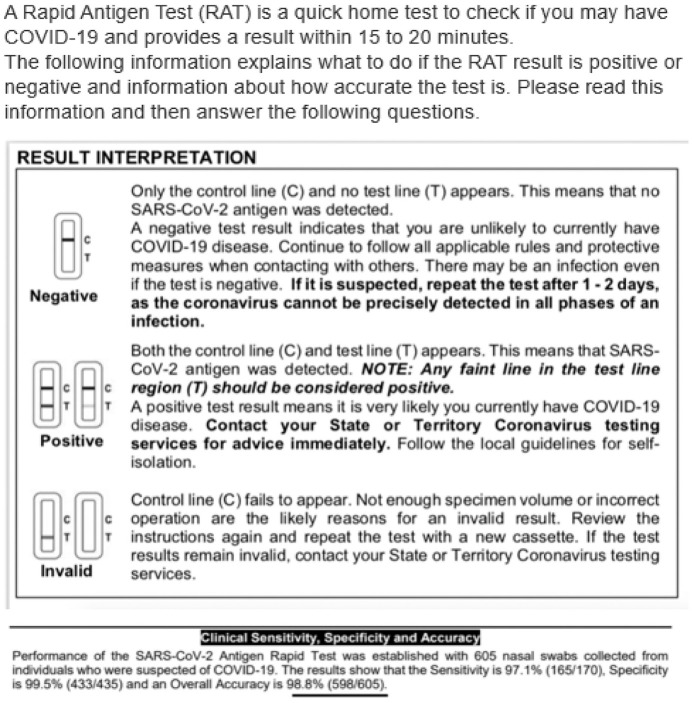
Diagnostic accuracy information for usual care. Presentation of clinical diagnostic accuracy estimates based on those supplied by the manufacturer of the Flowflex RAT and available online at the Australian Therapeutic Goods Authority Web site.^18^

The intervention arm used clinical sensitivity and specificity from the community-based study of the Flowflex RAT.^14^ This study was considered to be at low risk of bias.^10^ The diagnostic accuracy information was presented in health literacy–sensitive information using graphical methods (Figure 1). The content and format of the intervention was developed and evaluated using a “universal precautions” approach to health literacy.^19^ This included using the Sydney Health Literacy Lab Health Literacy Editor^20^ to simplify the language and reduce the grade reading level of the text, use of white space and formatting to reduce information overload, and inclusion of supporting images including icon arrays, in line with recommended best practice for patient decision aids.^21^ The usual care arm instructions were the Flowflex RAT instructions and diagnostic accuracy information provided in the Flowflex RAT kit by the manufacturer, downloaded from the Therapeutic Goods Administration Web site at the time of the study^8,18^ (Figure 2). The control arm had no information provided on the diagnostic accuracy of the test.

### Procedure

The questionnaire and randomization were created using Qualtrics, which uses the Mersenne Twister pseudorandom number generator to randomize participants.^22^ Participants and researchers were blinded to the allocation sequence until completion of data collection. After eligibility screening and provision of consent, participants were randomized, read the diagnostic accuracy information, and answered questions to see how well they understood the information (if in the intervention or usual care arms). They then read the hypothetical scenarios and completed outcome measures. Finally, participants answered demographic questions and a health literacy question (single-item literacy screener^23^). The full questionnaire is provided in the Supplement.

#### Outcome measures

Box 2 outlines the prespecified primary, secondary, and process outcome measures. For scenarios 4 and 5, an additional post hoc secondary outcome (not prespecified) was also analyzed: participants who indicated they would neither stay at home nor avoid people at higher risk. This variable was created from responses to the primary outcome and secondary outcome 5.

**Box 2:** Outcomes

**1. Primary outcome:** intention to self-isolate (yes/no; this was dichotomized from a 5-point scale: Yes (Stay at home without exception, except for shopping, except for work, except for shopping and work) v. No (Continue to leave the house as normal) **Secondary outcomes:** **2.** Would do a RAT (yes/no) **3.** Would isolate from other household members (yes/no/not applicable) **4.** Would do further tests (yes/no) **5.** Would avoid visiting people at higher risk of developing complications from COVID-19 (such as older people) (yes/no) **6.** Would avoid crowds (yes/no) **7.** Would keep 1.5 m away from others (yes/no) **8.** Would wash hands more often (yes/no) **9.** Would wear a mask (yes/no) **Process Outcomes** **10. Understanding of the test information.** This was measured in 3 ways: 1) perceived sensitivity and specificity of the test after reading the test information (0–100 for each), 2) perceived reading ease of the material (5-point item from very difficult to very easy), 3) perception on whether the information provided in the study helped the participant know what to do if the test result is positive/negative (5-point item ranging from *strongly disagree* to *strongly agree*). **11.** Participants’ perceived chance of being infected with COVID-19 or still being infectious with COVID-19. This outcome was measured in 2 ways: 1) 5-point response item ranging from *very unlikely* to *very likely* and 2) perceived chance between 1 and 100.

RAT, rapid antigen test.

#### Analysis

We summarized participants’ characteristics at baseline for the 3 randomized groups using frequency and percentages. We estimated the effects of the intervention and of usual care (relative to control) by comparing outcomes across randomized groups. To address the issue of repeated measurements nested within participants across the 5 scenarios, we used generalized estimated equation statistical models. All statistical analyses were conducted using R.^24^

#### Sample size

A sample size of 219 participants (73 per group) with 1:1:1 allocation to intervention or usual care or control groups was calculated to ensure that we would have 80% power to detect a pairwise difference in the proportion choosing to self-isolate as small as 10%. The assumptions were that 76% would choose to isolate in the control group for each scenario, a 10% dropout rate (individuals who do not complete the survey or who complete it faster than 1 min who are likely to have not read the survey questions), α = 0.05, the normal approximation to the binomial distribution, and the standard formula for comparing proportions in independent equal-sized groups.

### Ethics/Trial Registration

The Human Research Ethics Committee of the University of Sydney approved the study (2022/419), and it was registered with Australia New Zealand Clinical Trial Registry (ACTRN12622001517763). This study was piloted with a convenience sample of 25 individuals prior to data collection. This identified problems with the branching logic, typos, wording, formatting, and other minor changes, allowing correction.

The study is reported according to the Consolidated Standards of Reporting Trials (CONSORT) guidelines.^25^

## Results

**Figure 3:**
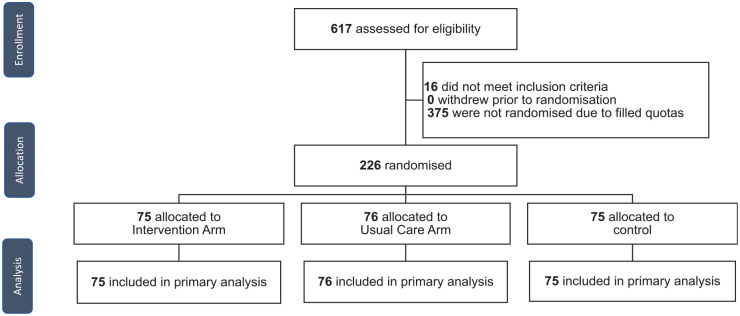
Study design and flow of participants.

**Figure 4:**
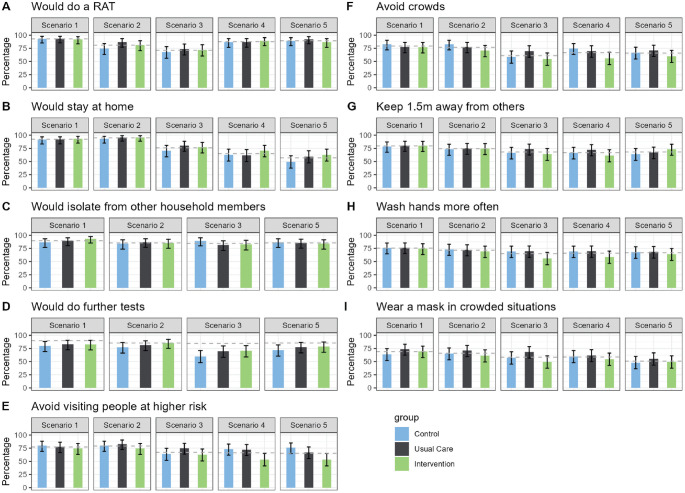
Effects of the intervention and usual care compared with control in each of 5 scenarios. (A) Would do a RAT (rapid antigen test), (B) Would stay at home, (C) Would isolate from other household members, (D) Would do further tests, (E) Would avoid visiting people at higher risk, (F) Would avoid crowds, (G) Would keep 1.5m away from others, (H) Would wash hands more often, and (I) Would wear a mask in crowded situations.

**Figure 5:**
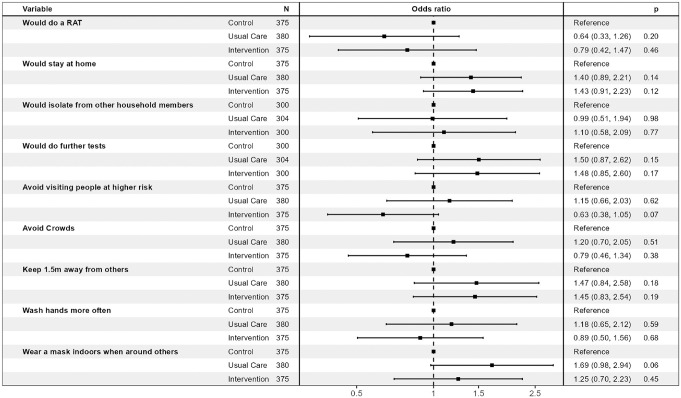
Effects of the intervention and usual care compared with control across the 5 scenarios (generalized estimating equation models).

The sample comprised 226 individuals randomized to the 3 arms (control *n* = 75, usual care *n* = 76, intervention *n* = 75; see Figure 3 for the flow of participants). There were no dropouts (all participants completed the survey, with the shortest completion time being 2 min). Characteristics were well-balanced across randomized label conditions, including the quota characteristics of location (state and territories), age, gender, and education (Table 1). Most participants were living with someone, born in Australia, non-Indigenous, spoke English at home, and had high health literacy. Nearly one-quarter (23%) had used a COVID-19 test in the past month, just less than half (42%) had a history of COVID-19 infection, and more than three-quarters (73%) had at least 3 doses of a COVID-19 vaccine. Just more than half (55%) were in full- or part-time work, and just less than half (46%) had an employer who supported them to work from home if sick. The results of separate scenario evaluations are presented in Table 2 and Figure 4, while Figure 5 presents the results when all scenarios are evaluated together within the generalized estimated equation model.

**Table 1:** Baseline Characteristics of Randomized Groups

Characteristic, *n* (%)	Control, *n* = 75	Usual Care, *n* = 76	Intervention, *n* = 75
Location			
Australian Capital Territory	2 (3)	2 (3)	1 (1)
New South Wales	17 (23)	19 (25)	12 (16)
Northern Territory	0 (0)	1 (1.3)	0 (0)
Queensland	20 (27)	12 (16)	14 (19)
South Australia	11 (15)	11 (15)	6 (8)
Tasmania	3 (4)	2 (3)	5 (7)
Victoria	15 (20)	20 (26)	19 (25)
Western Australia	7 (9)	9 (12)	18 (24)
Age, y			
18–29	10 (13)	12 (16)	17 (23)
30–39	19 (25)	24 (32)	30 (40)
40–49	9 (12)	10 (13)	4 (5)
50–59	9 (12)	9 (12)	3 (4)
60–69	19 (25)	12 (16)	7 (9)
≥70	9 (12)	9 (12)	14 (19)
Female	43 (57)	36 (47)	43 (57)
Living with someone	58 (77)	62 (82)	55 (73)
University diploma or degree	39 (52)	39 (51)	35 (47)
Born in Australia	58 (77)	64 (84)	57 (76)
Indigenous	0 (0)	4 (5)	6 (8)
Speak English at home	68 (91)	73 (96)	71 (95)
Confident filling out medical forms by self^a^	66 (88)	62 (82)	57 (76)
COVID-19 test in the past month	16 (21)	22 (29)	15 (20)
Have ever tested positive for COVID-19	34 (45)	28 (37)	34 (45)
Three or more doses of a COVID-19 vaccine	59 (79)	56 (74)	49 (65)
In full- or part-time work	40 (53)	42 (55)	42 (56)
Employer supports them to work from home if sick	37 (49)	34 (45)	33 (44)

a High health literacy indicated by responses that the person was “quite a bit” or “extremely” confident filling out medical forms by themselves.

**Table 2:** Effects of the Intervention and Usual Care Compared with Control in Each of the 5 Scenarios^a^

Scenario	Outcome	Control, *n* (%)	Usual Care, *n* (%)	Difference in Proportions Compared with Control (95% CI)	Intervention, *n* (%)	Difference in Proportions Compared with Control (95% CI)
Scenario 1 (very high posttest probability): symptoms, positive RAT	Would do a RAT	70 (93.3)	71 (93.4)	0.1 (−7.9, 8.1)	69 (92)	−1.3 (−11, 8.3)
	**Would stay at home**	**69 (92)**	**70 (92.1)**	**0.1 (−8.6, 8.8)**	**70 (93.3)**	**1.3 (−8.3, 11)**
	Would isolate from other household members	65 (86.7)	68 (89.5)	2.8 (-8.9,14.5)	70 (93.3)	6.7 (−4.2,17.5)
	Would do further tests	60 (80)	63 (82.9)	2.9 (−10.8, 16.6)	62 (82.7)	2.7 (−11.1, 16.5)
	Avoid visiting people at higher risk	60 (80)	59 (77.6)	−2.4 (−16.7, 12)	56 (74.7)	−5.3 (−20, 9.4)
	Avoid crowds	62 (82.7)	59 (77.6)	−5 (−19.1, 9)	58 (77.3)	−5.3 (−19.4, 8.8)
	Keep 1.5 m away from others	59 (78.7)	61 (80.3)	1.6 (−12.6, 15.8)	60 (80)	1.3 (−13, 15.6)
	Wash hands more often	57 (76)	58 (76.3)	0.3 (-13.6,14.2)	56 (74.7)	−1.3 (−16.5,13.8)
	Wear a mask in crowded situations	48 (64)	56 (73.7)	9.7 (−6.3, 25.7)	52 (69.3)	5.3 (−11.1, 21.7)
Scenario 2 (very high posttest probability): no symptoms, positive RAT × 2 (3 d ago and today)	Would do a RAT	56 (74.7)	66 (86.8)	12.2 (−1.6, 25.9)	61 (81.3)	6.7 (−7.9, 21.2)
	**Would stay at home**	**70 (93.3)**	**73 (96.1)**	**2.7 (−5.7, 11.2)**	**72 (96)**	**2.7** (−**5.8, 11.2)**
	Would isolate from other household members	63 (84)	66 (86.8)	2.8 (−9.7, 15.4)	64 (85.3)	1.3 (−11.5, 14.2)
	Would do further tests	58 (77.3)	62 (81.6)	4.2 (−10, 18.4)	64 (85.3)	8 (−5.7, 21.7)
	Avoid visiting people at higher risk	60 (80)	63 (82.9)	2.9 (−10.8, 16.6)	56 (74.7)	−5.3 (−20, 9.4)
	Avoid crowds	62 (82.7)	59 (77.6)	−5 (−19.1, 9)	53 (70.7)	−12 (−26.7, 2.7)
	Keep 1.5 m away from others	55 (73.3)	57 (75)	1.7 (−13.6, 17)	56 (74.7)	1.3 (−14, 16.7)
	Wash hands more often	55 (73.3)	55 (72.4)	−1 (−16.1, 14.2)	52 (69.3)	−4 (−19.8, 11.8)
	Wear a mask in crowded situations	49 (65.3)	54 (71.1)	5.7 (−10.4, 21.9)	46 (61.3)	−4 (−20.7, 12.7)
Scenario 3 (high posttest probability): no symptoms for 2 d, positive RAT (faint)	Would do a RAT	51 (68)	56 (73.7)	5.7 (-10.1,21.5)	54 (72)	4 (−12,20)
	**Would stay at home**	**53 (70.7)**	**61 (80.3)**	**9.6 (−5.4, 24.6)**	**58 (77.3)**	**6.7 (−8.7, 22)**
	Would isolate from other household members	67 (89.3)	62 (81.6)	−7.8 (−20.2, 4.7)	62 (82.7)	−6.7 (−19.1, 5.7)
	Would do further tests	45 (60)	53 (69.7)	9.7 (−6.7, 26.2)	53 (70.7)	10.7 (−5.8, 27.1)
	Avoid visiting people at higher risk	48 (64)	57 (75)	11 (−4.9, 26.9)	47 (62.7)	−1.3 (−18.1, 15.4)
	Avoid crowds	44 (58.7)	53 (69.7)	11.1 (−5.4, 27.6)	41 (54.7)	−4 (−21.2, 13.2)
	Keep 1.5 m away from others	50 (66.7)	56 (73.7)	7 (−8.9, 22.9)	48 (64)	−2.7 (−19.2, 13.9)
	Wash hands more often	52 (69.3)	53 (69.7)	0.4 (−14.7, 15.5)	42 (56)	−13.3 (−30, 3.3)
	Wear a mask in crowded situations	43 (57.3)	52 (68.4)	11.1 (−5.6, 27.7)	37 (49.3)	−8 (−25.2, 9.2)
Scenario 4 (moderately high posttest probability): symptoms, household close contact of confirmed case, negative RAT	Would do a RAT	65 (86.7)	66 (86.8)	0.2 (−10.8, 11.2)	67 (89.3)	2.7 (−9.1, 14.4)
	**Would stay at home**	**47 (62.7)**	**47 (61.8)**	**-0.8 (-17.1,15.5)**	**53 (70.7)**	**8 (−8.4,24.4)**
	Avoid visiting people at higher risk	55 (73.3)	55 (72.4)	−1 (−16.1, 14.2)	40 (53.3)	−20 (−36.4, −3.6)
	Avoid crowds	56 (74.7)	53 (69.7)	−4.9 (−20.5, 10.7)	42 (56)	−18.7 (−34.9, −2.4)
	Keep 1.5 m away from others	50 (66.7)	55 (72.4)	5.7 (−10.3, 21.7)	46 (61.3)	−5.3 (−22, 11.3)
	Wash hands more often	52 (69.3)	53 (69.7)	0.4 (−14.7, 15.5)	44 (58.7)	−10.7 (−27.3, 5.9)
	Wear a mask in crowded situations	45 (60)	47 (61.8)	1.8 (−15, 18.7)	41 (54.7)	−5.3 (−22.5, 11.8)
Scenario 5 (moderately high posttest probability): symptoms, close contact of confirmed case (dinner party), negative RAT	Would do a RAT	67 (89.3)	70 (92.1)	2.8 (−7.8, 13.3)	65 (86.7)	−2.7 (−14.4, 9.1)
	**Would stay at home**	**37 (49.3)**	**45 (59.2)**	**9.9 (−7.3, 27)**	**47 (62.7)**	**13.3 (−3.7, 30.4)**
	Would isolate from other household members	65 (86.7)	65 (85.5)	−1.1 (−13.3, 11)	63 (84)	−2.7 (−15.3, 10)
	Would do further tests	54 (72)	59 (77.6)	5.6 (−9.5, 20.8)	59 (78.7)	6.7 (−8.4, 21.8)
	Avoid visiting people at higher risk	57 (76)	51 (67.1)	−8.9 (−24.5, 6.7)	40 (53.3)	−22.7 (−38.9, −6.5)
	Avoid crowds	50 (66.7)	54 (71.1)	4.4 (−11.7, 20.5)	45 (60)	−6.7 (−23.4, 10.1)
	Keep 1.5 m away from others	48 (64)	51 (67.1)	3.1 (−13.4, 19.6)	55 (73.3)	9.3 (−6.8, 25.4)
	Wash hands more often	51 (68)	52 (68.4)	0.4 (−14.9, 15.7)	48 (64)	−4 (−20.5, 12.5)
	Wear a mask in crowded situations	36 (48)	42 (55.3)	7.3 (−10, 24.5)	37 (49.3)	1.3 (−16, 18.7)

RAT, rapid antigen test. Primary outcome = would stay at home.

### Process Outcomes

The proportion of participants who correctly recalled the test sensitivity provided to them was 72% in the intervention group (*n* = 54/75) and 80% in the usual care group (*n* = 61/76) (sensitivity provided was 52% for intervention and 97.1% for usual care groups; no information on test sensitivity was provided for the control group). The proportion of participants who indicated understanding of what this meant in terms of number of infected people who would test positive on the RAT was 64% in the intervention group (*n* = 48/75) and 50% in the usual care group (*n* = 38/76). The proportion of participants who correctly recalled the test specificity provided to them was 75% in the intervention (*n* = 56/75) and 78% in the usual care group (*n* = 59/76; specificity provided was 99% for intervention and 99.5% for usual care groups). The proportion of participants who indicated understanding of what this meant in terms of number of noninfected people who would test negative on the RAT was 57% in the intervention group (*n* = 43/75) and 24% in the usual care group (*n* = 18/76). The perceived reading ease and usefulness of the provided information was similar for the 2 groups, with most participants indicating that the information was easy/very easy to read and agreeing/strongly agreeing that the information helped them (Supplemental Table S1). The participants’ perceived probability of being infectious with COVID-19 was similar across all 3 randomized groups and was higher for scenario 1 than for the other scenarios (Supplemental Table S2).

### Primary Outcome

Table 2 and Figure 4 present the findings for the primary and secondary outcomes for the 5 scenarios. The proportions of participants who would self-isolate (primary outcome) were similar across the 3 randomized conditions for scenarios 1 to 3 (likely true positives). The proportions who would self-isolate were higher for those in the intervention condition than the usual care or control conditions for scenarios 4 and 5 (likely false negatives), although these differences were not statistically significant. The largest potential effect of the intervention was seen in scenario 5 (dinner party with confirmed cases, person has symptoms). Here, 62.7% of the intervention group indicated that they would self-isolate, compared with 49.3% of the control group (absolute difference 13.3%, 95% confidence interval [CI]: −2% to 30%, *P* = 0.10). When participants’ responses across scenarios were combined in the generalized estimated equation model, the odds ratio for self-isolation was 1.43 (95% CI: 0.91 to 2.23) for intervention versus control (*P* = 0.12; Figure 5).

### Secondary Outcomes

The proportion of participants who stated they would use a RAT after being presented with the scenario information was consistently high across the 3 randomized groups. The lowest proportions were seen for scenario 3 (day 6 after a positive RAT result, 2 d without symptoms, Figure 2 second row), in which 71% of the 226 participants indicated they would do a test. The proportion who would report the positive RAT result (only asked for scenario 1) was also high and similar across the 3 randomized groups, with 85% of the overall sample stating that they would do this (control group: 80% [95% CI 70% to 88%], usual care: 90% [95% CI 81% to 95%], intervention 87% [95% CI 78% to 93%]). Of those who indicated they would self-isolate, most would also isolate from other household members, and this was again similar across the 3 randomized groups. This was lowest for scenario 3, in which 80% of *n* = 150 participants who would self-isolate and who did not live alone indicated they would also isolate from others in their house.

Although the “further preventative actions” was intended to indicate actions taken after self-isolation, many participants appeared to not interpret it this way. In retrospect the wording for this question was unclear as it did not specify the timing for further actions (“What further actions would you take (select all that apply)?”; see full questionnaire in the Supplement). This resulted in a lower proportion stating they would avoid visiting people at higher risk of developing complications from COVID-19 (such as older people) in the Intervention group compared to the Control and Usual Care groups in all Scenarios (Table 2, Figure 2 row E). The differences were largest in in the negative RAT result Scenarios 4 and 5. The odds ratio for avoiding visiting higher risk people for the 5 scenarios combined in the generalized estimating equation model was 0.63 (0.38 to 1.05; *P* = 0.07; Figure 5). Post hoc analyses found that these differences were driven by people who said they would not avoid visiting people at higher risk because they would be self-isolating at home (Supplemental Table S3). After viewing the likely false-negative test results in scenario 4 (household contact of confirmed case, with symptoms) and scenario 5 (close contact of known cases at a dinner party, with symptoms), the proportion of each randomized group who said they would neither stay at home nor avoid people at higher risk was the same for both scenarios: 24.0% of the intervention group compared with 20.0% of the control group (absolute difference 4.0%, 95% CI: −10.6% to 18.6%, *P* = 0.69). In the overall sample, 42 people (18.6%) stated they would take neither preventative action.

The proportions who would get further tests, avoid crowds, keep 1.5 m away from others, wash hands more often, and wear a mask in crowded situations were similar across the 3 randomized groups. The proportion of the overall sample who would take each of these further actions ranged from 51% (wear a mask in crowded situations, scenario 5) to 82% (get further tests, scenario 1; Table 2, Figure 2 rows D–I).

## Discussion

We found strong evidence that health literacy–sensitive formatting supported participant understanding and recall of diagnostic accuracy information for a COVID-19 RAT. Most participants correctly recalled the diagnostic test accuracy estimates that were provided to them, and more of the intervention group correctly interpreted the meaning of these (*P* = 0.08 for understanding sensitivity, *P* < 0.001 for understanding specificity). More than double the participants in the intervention group indicated that they understood what the specificity meant relative to those in the usual care group.

Despite participants being more informed in the intervention group, this did not translate into statistically significant increases in intentions to isolate and prevent onward spread. Nevertheless, we found possible beneficial effects on intentions to isolate across all scenarios, strongest in scenario 5 (where the overall sample were least likely to isolate). In this scenario, there was an absolute difference of 13% more who would choose to self-isolate.

An apparent possible harm of the intervention, with a higher proportion of people in the intervention group expressing intent to visit high-risk individuals, appears to be an artifact of the questionnaire design. Further analysis revealed that the proportion who reported they would neither self-isolate nor avoid visiting higher risk people was similar across randomized groups. In the overall sample, most people would do and report the test, but fewer people would do additional actions in the likely false-negative scenarios such as wearing a mask in a crowded place (51% in scenario 5). Of concern, nearly 20% of the overall sample stated that they would neither self-isolate nor avoid visiting higher risk people after receiving a negative result in scenario 5, which we considered to have a moderately high posttest probability of infection.

Many studies have found that the sensitivity of RATs is lower in a real-world setting because of factors relating to the study population and the implementation of the test.^6,10,26,27^ Patient factors include the viral load for infected patients, whether the individual is asymptomatic, and the severity of the illness if symptomatic.^6^ Implementation factors include whether the testing is completed within 7 d from symptom onset and whether the sample is self-collected or collected by a health professional.^10,26,27^ However, knowledge of the poorer diagnostic accuracy of RATs when used by a self-test by consumers in real-world settings appears to low in the general community.^28^ At the time of our study (and still the case now), published information on the accuracy of RATs on the Australian Government’s Therapeutic Goods Association Web site (provided by test manufacturers) indicated substantially higher sensitivity than actual performance when used as intended—as a self-test by community members.^10^

Others have also undertaken research to address the need for better communication about RATs for self-testing use. A randomized online study of the interpretation of COVID-19 self-test results by 360 adults in the United States recruited in April 2021 found that after receiving likely false-negative results, participants receiving the intervention instructions (based on decision science principles) were more likely to self-isolate than participants receiving the standard authorized instructions or no instructions.^11^ That study focused on optimizing the instructions to support the person to undertake appropriate further actions, whereas our study focused on optimizing communicating the high risk of a false-negative result. Both studies found positive effects for the interventions that were similar size (absolute difference of 13% for our study and 14% for the US study). Whereas the US study found statistically significant benefits against the usual care condition but not the control, we found larger benefits against the control than usual care (and in our study, neither comparison was statistically significant).

The strengths of the study include its randomized design to investigate the effects of optimizing communication about COVID-19 RAT diagnostic accuracy on people’s intentions to self-isolate and take other preventative actions. This study contributes high-quality evidence to the body of research investigating how to best communicate test results to patients and other community members and to the communication about COVID-19 RATs in particular. This study sample included geographical, gender, and age diversity; however, it was not representative of the overall Australian population on many other characteristics (higher proportion of participants were born in Australia, spoke English at home, had a university diploma or degree, and had higher health literacy than the general population did).

As well as a sample with several indicators of more socioeconomic advantage than the general community, there were several other limitations. Although the sample size was chosen based on reasonable assumptions, it is likely that we did not have sufficient statistical power to detect effects of the intervention that did truly exist. Furthermore, the hypothetical nature of the online experimental design of the study means that our findings may not generalize to what participants would do in real life—although the high proportion who had past COVID-19 infection and the prominence of the pandemic in day-to-day life at the time of the study may mitigate against that. A follow-up study investigating what participants actually did after contracting COVID-19 infection, rather than their stated intentions, would be informative. Further research to explore the relative weighting that people place on symptoms versus test results may also be helpful. The likely misinterpretation of the questions regarding further preventative actions that indicated actions taken instead of self-isolation instead of after self-isolation indicates poor wording of this element of the questionnaire that was not detected on pilot testing. We did not provide information to participants about why the sensitivity of the test was suboptimal, and doing so might have helped them to conceptualize why results may be unreliable. This may have more effectively countered manufacturer information on the Australian Government’s Therapeutic Goods Authority Web page and in the test kits themselves that indicated high sensitivity for COVID-19 RATs. The study was conducted in December 2022 when mandatory self-isolation had only recently been uplifted. The proportion of people in the control group indicating they would take preventative behaviors was very high, suggesting likely ceiling effects. It is likely that fewer people in the control group would self-isolate now, given the longer period since the restrictions were lifted and changing societal expectations about preventative behaviors. It is possible that the intervention would have a larger effect now. In addition, some participants may have already been aware of the risk of a false-negative result from information outside of the study, including past experience with RATs; this would have lessened the contrast across randomized groups. Finally, our study design did not allow separate evaluation of the 2 components of the intervention (different diagnostic accuracy information and health literacy sensitive presentation).

Australia now relies on the public to choose to self-isolate and take other precautionary measures for COVID-19 prevention and control, and self-RATs play a pivotal role in this. The evidence base for the diagnostic accuracy of COVID-19 self-RAT when performed in a real-life setting and as intended (i.e., unsupervised consumer collection and interpretation) suggests a high risk of false-negative results.^10^ Concerted efforts are needed to increase community awareness of the issue and to prevent the false interpretation that 1 negative result can rule out infection when there is suspicion of COVID-19.

Although our intervention increased the understanding of both test sensitivity and specificity, we found a stronger effect for the latter. Future research (for COVID-19, or other conditions) could build on this finding and explore the impacts of providing health literacy–sensitive diagnostic accuracy information in scenarios where it is likely the individual does not have the target condition (i.e., likely true-negative and likely false-positive results). For COVID-19 RATs, these results would indicate scenarios where self-isolation is unnecessary.

## Conclusion

This study shines a light on the need for better communication on the limitations of self-tests. In this way, the public can make better informed decisions about preventative behaviors that limit the onward spread of infection.

## Supplemental Material

sj-pdf-1-mdm-10.1177_0272989X241242131 – Supplemental material for Communicating the Imperfect Diagnostic Accuracy of COVID-19 Rapid Antigen Self-Tests: An Online Randomized Experiment Supplemental material, sj-pdf-1-mdm-10.1177_0272989X241242131 for Communicating the Imperfect Diagnostic Accuracy of COVID-19 Rapid Antigen Self-Tests: An Online Randomized Experiment by Huijun Li, Megha Kalra, Lin Zhu, Deonna M. Ackermann, Melody Taba, Carissa Bonner and Katy J.L. Bell in Medical Decision Making

## References

[1] World Health Organization. Statement on the fifteenth meeting of the IHR (2005) Emergency Committee on the COVID-19 pandemic. 2023. Available from: https://www.who.int/news/item/05-05-2023-statement-on-the-fifteenth-meeting-of-the-international-health-regulations-(2005)-emergency-committee-regarding-the-coronavirus-disease-(covid-19)-pandemic

[2] Barbour V. COVID-19: no longer a global health emergency, now a long term challenge. Med J Aust. 2023;218(10):437. 37270790 10.5694/mja2.51976

[3] World Health Organization. Considerations for implementing and adjusting public health and social measures in the context of COVID-19. Interim guidance. March 30, 2023. Available from: https://www.who.int/publications/i/item/who-2019-ncov-adjusting-ph-measures-2023.1

[4] World Health Organization. WHO policy brief: COVID-19 testing. September 14, 2022. Available from: https://www.who.int/publications/i/item/WHO-2019-nCoV-Policy_Brief-Testing-2022.1

[5] Meumann EM Robson JMB . Testing for COVID-19: a 2023 update. Aust Prescr. 2023;46(1):13–7. 10.18773/austprescr.2023.007 PMC10664091 38053664

[6] Dinnes J Sharma P Berhane S , et al. Rapid, point-of-care antigen tests for diagnosis of SARS-CoV-2 infection. Cochrane Database Syst Rev. 2022;7(7):CD013705. 10.1002/14651858.CD013705.pub3 PMC9305720 35866452

[7] Polechová J Johnson KD Payne P , et al. SARS-CoV-2 rapid antigen tests provide benefits for epidemic control—observations from Austrian schools. J Clin Epidemiol. 2022;145:14–9. 10.1016/j.jclinepi.2022.01.002 PMC8760838 35041972

[8] Australian Therapeutic Goods Administration (TGA). COVID-19 rapid antigen self-tests that are approved in Australia. Last updated May 1, 2023. Available from: https://www.tga.gov.au/products/covid-19/covid-19-tests/covid-19-rapid-antigen-self-tests-home-use/covid-19-rapid-antigen-self-tests-are-approved-australia

[9] World Health Organization. SARS-CoV-2 Antigen-Detecting Rapid Diagnostic Tests: An Implementation Guide. Geneva (Switzerland): World Health Organization; 2020.

[10] Bell KJL Li Y Medcalf E Ackermann D . COVID-19 rapid antigen self-tests approved for use in Australia: a systematic review of published diagnostic test accuracy studies and comparison to manufacturer supplied information. Med J Aust. 2023;219(11):551–8. 10.5694/mja2.52151 PMC10952141 37903650

[11] Woloshin S Dewitt B Krishnamurti T Fischhoff B. Assessing how consumers interpret and act on results from at-home COVID-19 self-test kits: a randomized clinical trial. JAMA Intern Med. 2022;182(3):332–41. 10.1001/jamainternmed.2021.8075 PMC8804977 35099501

[12] Bonner C Batcup C Cvejic E , et al. Addressing behavioral barriers to COVID-19 testing with health literacy–sensitive eHealth interventions: results from 2 national surveys and 2 randomized experiments. JMIR Public Health Surveill. 2023;9:e40441. 10.2196/40441 PMC10337324 37172319

[13] Fulmer AA Abboud GA 2nd Wallace LS. Health literacy characteristics of over-the-counter rapid antigen COVID-19 test materials. Res Social Adm Pharm. 2022;18(12):4124–8. 10.1016/j.sapharm.2022.08.003 PMC9376145 35987673

[14] Schuit E Venekamp RP Hooft L , et al. Diagnostic accuracy of covid-19 rapid antigen tests with unsupervised self-sampling in people with symptoms in the omicron period: cross sectional study. BMJ. 2022;378:e071215. 36104069 10.1136/bmj-2022-071215 PMC9471225

[15] Li H Ackermann D Bonner C Kalra M Taba M Bell K . Assessing communication strategies for COVID-19 rapid antigen self-tests: a protocol for a randomised experiment. 2023. Available from: osf.io/jskh5

[16] Australian Government Department of Health and Aged Care C-EaST. COVID-19 Australia: epidemiology report 69 reporting period ending 18 December 2022. Commun Dis Intell (2018). 2023;47. 10.33321/cdi.2023.47.7 36775808

[17] Bell KJL Stanaway FF Irwig LM Horvath AR Teixeira-Pinto A Loy C . How to use imperfect tests for COVID-19 (SARS-CoV-2) to make clinical decisions. Med J Aust. 2021;214(2):69–73.e1. 10.5694/mja2.50907 33415725

[18] Flowflex. SARS-CoV-2 antigen rapid test (self-esting) package insert. 2022. Available from: https://www.tga.gov.au/sites/default/files/covid-19-rapid-antigen-self-tests-are-approved-australia-ifu-382031-02.pdf

[19] Agency for Healthcare Research and Quality (AHRQ). Health literacy universal precautions toolkit. Content last reviewed September 2020. Agency for Healthcare Research and Quality, Rockville, MD. Available from: https://www.ahrq.gov/health-literacy/improve/precautions/index.html

[20] Ayre J Bonner C Muscat D , et al. Multiple automated health literacy assessments of written health information: development of the SHeLL (Sydney Health Literacy Lab) Health Literacy Editor v1. JMIR Form Res. 2023;7:e40645. 10.2196/40645 PMC9975914 36787164

[21] Bonner C Trevena LJ Gaissmaier W , et al. Current best practice for presenting probabilities in patient decision aids: fundamental principles. Med Decis Making. 2021;41(7):821–33. 10.1177/0272989X21996328 33660551

[22] Muscat DM Morris GM Bell K , et al. Benefits and harms of hypertension and high-normal labels: a randomized experiment. Cir Cardiovasc Qual Outcomes. 2021;14(4):e007160. 10.1161/CIRCOUTCOMES.120.007160 33813855

[23] Morris NS MacLean CD Chew LD Littenberg B. The single item literacy screener: evaluation of a brief instrument to identify limited reading ability. BMC Family Pract. 2006;7(1):21. 10.1186/1471-2296-7-21 PMC1435902 16563164

[24] R Core Team RFfSC. R: A LANGUAGE and Environment for Statistical Computing. Vienna (Austria): R Core Team; 2023. Available from: https://www.R-project.org

[25] Schulz KF Altman DG Moher D. CONSORT 2010 statement: updated guidelines for reporting parallel group randomised trials. BMJ. 2010;340:c332. 10.1136/bmj.c332 PMC2844940 20332509

[26] Deeks JJ Singanayagam A Houston H , et al. SARS-CoV-2 antigen lateral flow tests for detecting infectious people: linked data analysis. BMJ. 2022;376:e066871. 10.1136/bmj-2021-066871 PMC8864475 35197270

[27] Parvu V Gary DS Mann J , et al. Factors that influence the reported sensitivity of rapid antigen testing for SARS-CoV-2. Front Microbiol. 2021;12:714242. 34675892 10.3389/fmicb.2021.714242 PMC8524138

[28] Shores EA Berry J. Diagnostic accuracy of thirteen COVID-19 (SARS-CoV-2) rapid antigen self-tests with very high sensitivity approved for home use in Australia. Aust N Z J Public Health. 2022;46(5):722–3. 10.1111/1753-6405.13285 PMC9538302 35924882

