# Supplementary material for: Communicating the Imperfect Diagnostic Accuracy of COVID-19 Rapid Antigen Self-Tests: An Online Randomized Experiment
Source: Med Decis Making. 2024 Apr 23;44(4):437–50. doi: 10.1177/0272989X241242131 (PMC11102651; doi:10.1177/0272989X241242131)
Supplement: sj-pdf-1-mdm-10.1177_0272989X241242131 – Supplemental material for Communicating the Imperfect Diagnostic Accuracy of COVID-19 Rapid Antigen Self-Tests: An Online Randomized Experiment [file sj-pdf-1-mdm-10.1177_0272989X241242131.pdf]

Supplement

Supplementary Table 1: Understanding of Diagnostic Accuracy information in Intervention and Usual Care

	Usual Care (n=76)	Intervention (n=75)	P value
**What is the sensitivity of the RAT?**	(Sensitivity=97.1%)	(Sensitivity=52%)	
median (Q1, Q3)	97.1 (97.1,97.1)	52 (52,52)	
Min, Max	0,100	0.25, 99	
N (%) with correct response	61 (80%)	54 (72%)	0.25
**Out of 100 people who have COVID-19, how many would test positive on the RAT?**			
Median (Q1, Q3)	98 (97,98.8)	52 (52,52)	
Min, Max	0,598	1,99	
N (%) with correct response[1]	38 (50%)	48 (64%)	0.08
**Confidence**			
Median (Q1, Q3)	75 (48.5,89.2)	73 (50.5,88.5)	0.379
Min, Max	0,100	8,100	
**What is the specificity of the RAT?**	(Specificity=99.5%)	(Specificity=99%)	
Median (Q1, Q3)	99.5 (99.4,99.5)	99 (94.5,99)	0.03
Min, Max	0,100	1,99	
N (%) with correct response	59 (78%)	56 (75%)	0.66
**Out of 100 people who do not have COVID-19, how many would test negative on the RAT?**			
Median (IQR)	90 (2,99)	99 (48, 99)	
Min, Max	0,433	1,99	
N (%) with correct response[2]	18 (24%)	43 (57%)	< 0.001
**Confidence**			
Median (Q1, Q3)	66 (45,84)	76 (51,93)	
Min, Max	0,100	4,100	
**How easy or difficult was it to read the information about interpreting RAT results?**			
Very Easy	23 (30.3)	14 (18.7)	0.30
Easy	20 (26.3)	31 (41.3)	
Neutral	23 (30.3)	22 (29.3)	
Difficult	7 (9.2)	5 (6.7)	
Very difficult	3 (3.9)	3 (4)	
**The information helped me know what to do if test result is positive.**			
Strongly agree	24 (31.6)	16 (21.3)	0.33
Agree	32 (42.1)	37 (49.3)	
Neutral	17 (22.4)	15 (20)	
Disagree	1 (1.3)	5 (6.7)	
Strongly disagree	2 (2.6)	2 (2.7)	
**The information helped me know what to do if test result is negative.**			
Strongly agree	22 (28.9)	15 (20)	0.65
Agree	38 (50)	45 (60)	
Neutral	13 (17.1)	11 (14.7)	
Disagree	1 (1.3)	2 (2.7)	
Strongly disagree	2 (2.6)	2 (2.7)	

Notes 1. 97, 97.1, 98 accepted as correct responses.; 2. 99, 99.5 accepted as correct responses.

Supplementary Table 2: Participants' estimations of how likely it is that they (or household member) are infectious in Scenarios 2-5

Measure	Scenario 2			Scenario 3			Scenario 4			Scenario 5		
Randomised Group	Control	Usual Care	Interven-tion	Control	Usual Care	Interven-tion	Control	Usual Care	Interven-tion	Control	Usual Care	Interven-tion
Think it is likely or very likely that they are infectious, n (%)	61 (81)	65 (86)	61 (81)	24 (32)	23 (30)	31 (41)	21 (28)	16 (21)	19 (25)	23 (31)	18 (24)	15 (20)
Estimated probability that they are infectious, median (IQR)	74 (50,87)	78.5 (58.2,96)	70 (51,87)	50 (24.5,69)	51.5 (34,75)	50 (29,71.5)	50 (24.2,75)	50 (25,64.2)	50 (24,55)	49 (16,62.5)	50 (23.8,59)	50 (24.5,59.5)
Confidence in their response, median (IQR)	76 (57,96)	75 (52,92.2)	71 (51,83.5)	75 (50,89.5)	59 (51,79)	66.5 (50,86.8)	75 (50,90.5)	62.5 (50,81)	61 (50,80.5)	62 (50,90)	56 (50,79)	65 (50,78)

Notes

Scenario 2: True positive, Day 4 - no symptoms, Scenario 3: True positive, Day 6 (faint line) – no symptoms, Scenario 4: False negative: Household contact of known case, with symptoms, Scenario 5: False negative, close contact of known cases (dinner party) with symptoms

Supplementary Table 3: Analysis of post-hoc secondary outcome: neither stay at home nor avoid visiting people at higher risk

Scenario 4: False negative: Household contact of known case, with symptoms

Control group

		Avoid visiting people at higher risk		
		Yes	No	Total
Stay at home	Yes	42	5	47
	No	13	**15**	28
	Total	55	30	75

Usual care

		Avoid visiting people at higher risk		
		Yes	No	Total
Stay at home	Yes	35	12	47
	No	20	**9**	29
	Total	55	21	76

Intervention

		Avoid visiting people at higher risk		
		Yes	No	Total
Stay at home	Yes	36	17	53
	No	4	**18**	22
	Total	40	35	75

Proportions of each group would both not stay at home and not avoid visiting people at higher risk
Control = 15/75= 20%
Usual care = 9/76 = 12%
Intervention 18/75 = 24% P value = 0.69 (vs Control)

Scenario 5: False negative: Close contact of known cases (dinner party) with symptoms

Control group

		Avoid visiting people at higher risk		
		Yes	No	Total
Stay at home	Yes	34	3	37
	No	23	**15**	38
	Total	57	18	75

Usual care

		Avoid visiting people at higher risk		
		Yes	No	Total
Stay at home	Yes	31	14	45
	No	20	**11**	31
	Total	51	25	76

Intervention

		Avoid visiting people at higher risk		
		Yes	No	Total
Stay at home	Yes	30	17	47
	No	10	**18**	28
	Total	40	35	75

Proportions of each group would both not stay at home and not avoid visiting people at higher risk
Control = 15/75= 20%
Usual care = 11/76 = 14%
Intervention 18/75 = 24% P value = 0.69 (vs Control)

Supplement 4: Survey questions

Introduction and Consent

By taking part in this study, you'll be helping researchers at the University of Sydney create resources to stop the spread of COVID-19 in the community.

What's this study about?
In this study, we are investigating how people in the Australian community are using COVID 19 rapid antigen self-tests (RATs), and the effects of different ways of communicating about these tests.

What will the study involve for me?
If you agree to take part in this study, you will be asked to complete an online survey. You will be asked questions about yourself (e.g., your age, gender, and the postcode for where you live). After this you will be shown different scenarios and asked about what you would do next after receiving a particular RAT result.

The survey will take about 10-20 minutes to finish.

What else do I need to know?
Please note the survey is voluntary and you don’t have to take part in this survey if you don’t want to. You have the choice to leave the survey at any time. We won’t store your name or any contact information.

To take part, you need to be over 18 years old and be able to read and understand English. For more information, please download read the participant information sheet here .

This study has received Ethics approval from The University of Sydney Ethics Committee.

I agree to take part in this research study.
In giving my consent I state that:

1. I understand the purpose of the study, what I will be asked to do, and any risks/benefits involved.
2. I have read the Participant Information Statement and have been able to discuss my involvement in the study with the researchers if I wished to do so.

3. The researchers have answered any questions that I had about the study, and I am happy with the answers.
4. I understand that being in this study is completely voluntary and I do not have to take part. My decision whether to be in the study will not affect my relationship with the researchers or anyone else at The University of Sydney now or in the future.
5. I understand that I can withdraw from the study at any time.
6. I understand that my questionnaire responses cannot be withdrawn once they are submitted, as they are anonymous and therefore the researchers will not be able to tell which one is mine.
7. I understand that personal information about me that is collected over the course of this project will be stored securely and will only be used for purposes that I have agreed to. I understand that information about me will only be told to others with my permission, except as required by law.
8. I understand that the information collected for this study may be used in future projects. I understand that by providing my consent I am allowing use of my information in future projects (subject to further ethical approval).
9. I understand that the results of this study may be published, and that publications will not contain my name or any identifiable information about me.

I consent:

- ◯ Yes
- ◯ No

Do you commit to providing thoughtful answers to the questions in this survey?

- ◯ Yes, I will
- ◯ No, I will not
- ◯ I can't promise either way

Demographic Questions 1

How old are you (in years)?

- ◯ 18-29 years
- ◯ 30-39 years
- ◯ 40-49 years
- ◯ 50-59 years
- ◯ 60-69 years
- ◯ 70 years or above

Which of the following best describes your current gender identity?

- ◯ Male
- ◯ Female
- ◯ Non-binary/gender fluid/gender non-conforming
- ◯ [ ] Different identity
- ◯ Choose not to disclose

What is your highest level of education?

- ◯ Less than high school
- ◯ High school graduate
- ◯ Certificate I or II
- ◯ Certificate III or IV
- ◯ Diploma, Bachelor degree, or equivalent
- ◯ Masters or Doctoral degree, or equivalent

Health Lit Intervention

A Rapid Antigen Test (RAT) is a quick home test to check if you may have COVID-19 and provides a result within 15 to 20 minutes.
The following information explains what to do if the RAT result is positive or negative and information about how accurate the test is. Please read this information and then answer the following questions.

INVALID RESULT (Test did not work):
A control line may not appear for two reasons:
1. You have not used enough oral fluid.
2. You made a mistake when doing the test.

Please re-check the instructions and repeat the test using a new test kit. You can also contact our COVID-19 test centre.

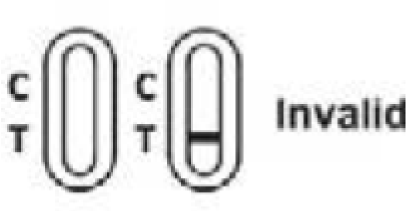

POSITIVE RESULT:

- A positive result has two coloured lines: one in the control region (C) and one coloured line in the test region (T).
- The amount of colour in the Test region (T) may vary. **Even a very faint line is positive and shows that there is infectious virus present.**
- **A positive result means you are highly likely to have COVID and to be infectious – you are still infectious even if you have already isolated for several days and no longer have symptoms.**
- Further RATs may be done to confirm the positive result. You may do a PCR test to confirm your result.
- You should self-isolate now and follow guidelines for a positive case. Contact your doctor as needed.

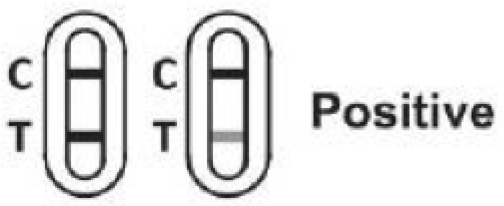

NEGATIVE RESULT:

- A negative result has one coloured line: one in the control region (C) and none in the test region (T).

- You are less likely to have COVID-19, but it is possible negative result can be wrong sometimes. This means you may still have COVID-19 when the test result is negative.
- Symptoms may include sore throat, runny nose, headaches, fever, and loss of sense of smell or taste. If you have symptoms, but the test is negative, you can repeat the RAT after 1-2 days or do a PCR.
- Sometimes the virus is not detected at the start of the infection. If you are unwell, contact your doctor. Even with a negative test result you should still protect yourself and others from COVID-19. Follow your doctor's advice.

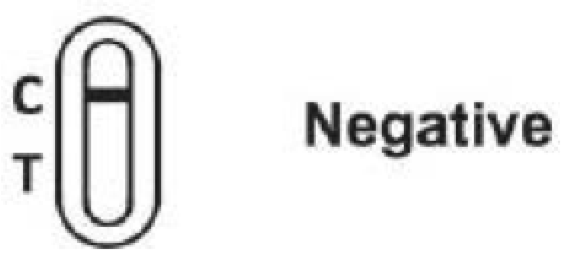

SENSITIVITY = 52%

The **sensitivity** of a COVID-19 Rapid Antigen Test (RAT) is **52%.** This means out of 100 people who have COVID-19, 52 will be positive on the RAT. But 48 people will be falsely negative.

The "sensitivity" tells you how well the test can detect COVID-19. A high number

means it picks up COVID-19 most of the time. A low number means a lot of COVID-19 cases will be missed.

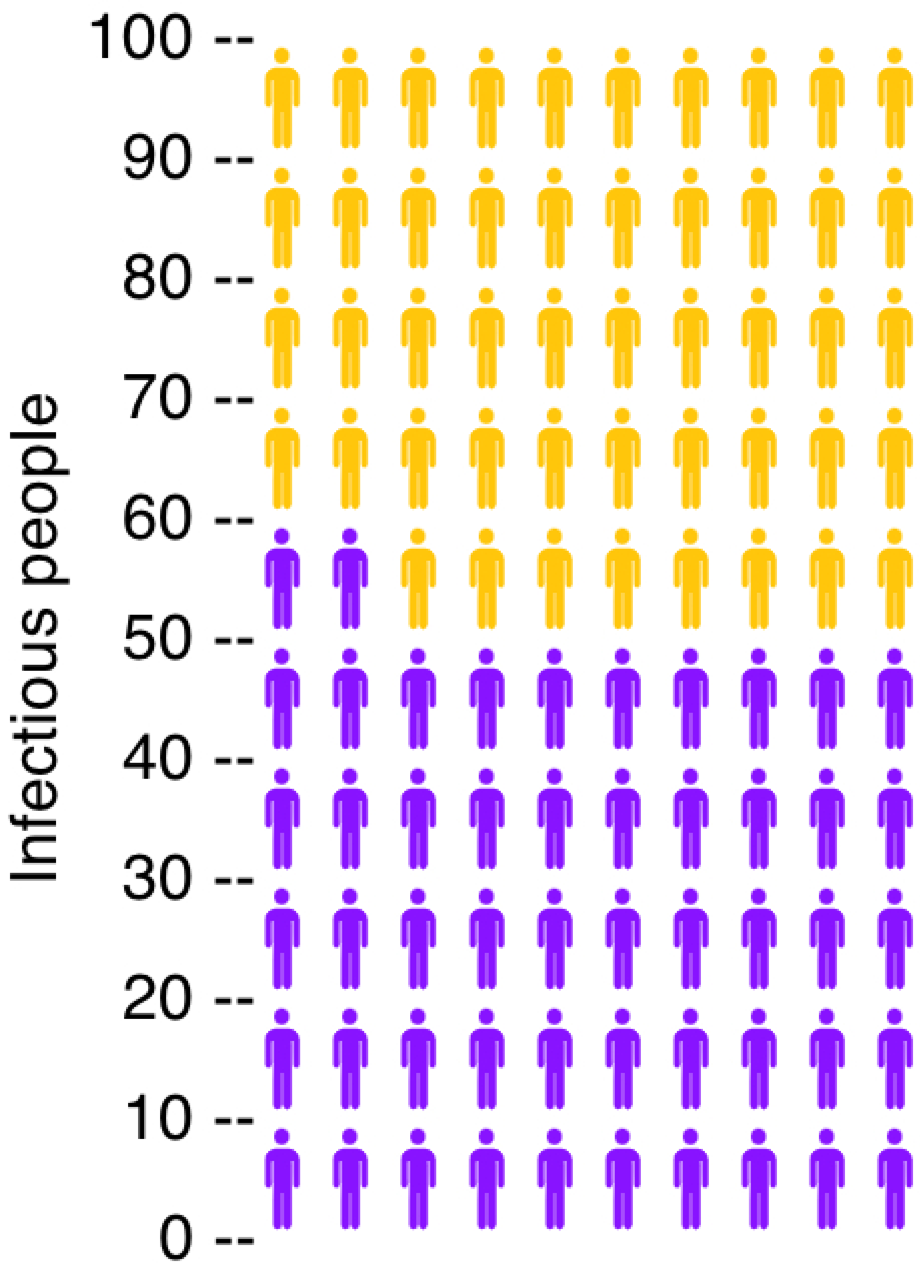
Infectious people
100 --
90 --
80 --
70 --
60 --
50 --
40 --
30 --
20 --
10 --
0 --

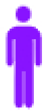

52 out of 100 people who have COVID-19 will be positive on the RAT

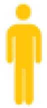

48 out of 100 people who have COVID-19 will NOT be positive on the RAT

SPECIFICITY = 99%

The **specificity** of a COVID-19 Rapid Antigen Test (RAT) is **99%.** This means out of 100 people who do not have COVID-19, 99 will be negative on the RAT. But 1 person will be falsely positive.

The "specificity" tells you how well the test can confirm the absence of COVID-19. A high number means the test will correctly identify people who do not have COVID-19. This means there will be less false positive results.

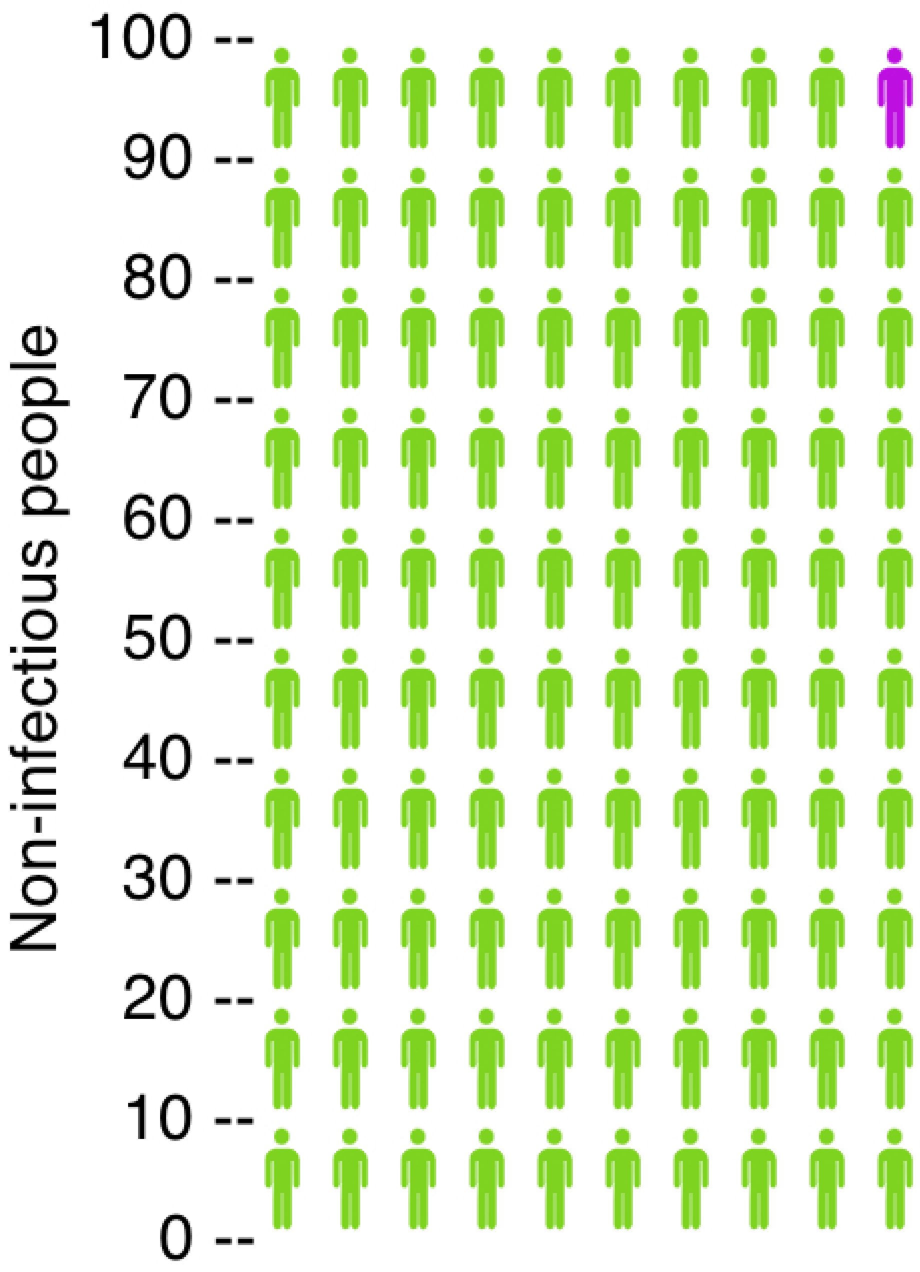
Non-infectious people
100 --
90 --
80 --
70 --
60 --
50 --
40 --
30 --
20 --
10 --
0 --

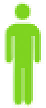

99 out of 100 people who do NOT have COVID-19 will be negative on the RAT

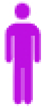

1 out of 100 people who do NOT have COVID-19 will be falsely positive on the RAT

Now that you have read the RAT information, please answer the following questions.

What is the sensitivity of the RAT as a %?

Out of 100 people who have COVID-19, how many would test positive on the

RAT?

How confident are you in your response above?

	Not confident at all		Medium level of confidence		Absolutely confident
	0	25	50	75	100
Confidence Level					

What is the specificity of the RAT as a %?

Out of 100 people who do not have COVID-19, how many would test negative on the RAT?

How confident are you in your response above?

	Not confident at all	Medium level of confidence		Absolutely confident
	0	25	50	75	100
Confidence Level					

How easy or difficult was it to read the information about interpreting RAT results?

- Very Easy
- Easy
- Neutral
- Difficult
- Very difficult

The information helped me know what to do if test result is positive.

- Strongly agree
- Agree
- Neutral
- Disagree
- Strongly disagree

The information helped me know what to do if test result is negative.

- Strongly agree
- Agree
- Neutral
- Disagree
- Strongly disagree

We will now present five different scenarios relating to COVID-19. Based on the scenarios, we will require you to answer how likely it is that you (or a member of your household) has a COVID-19 infection, and about specific actions you would take to prevent onward spread of infection.

Scenario 1/5:
Imagine you have been unwell with symptoms including headache, sore throat, fever, runny nose, and loss of taste and smell. Would you do a RAT?

- Yes
- No

You do a RAT and the result appears as a strong positive line. Based on the information provided, please answer the following questions.

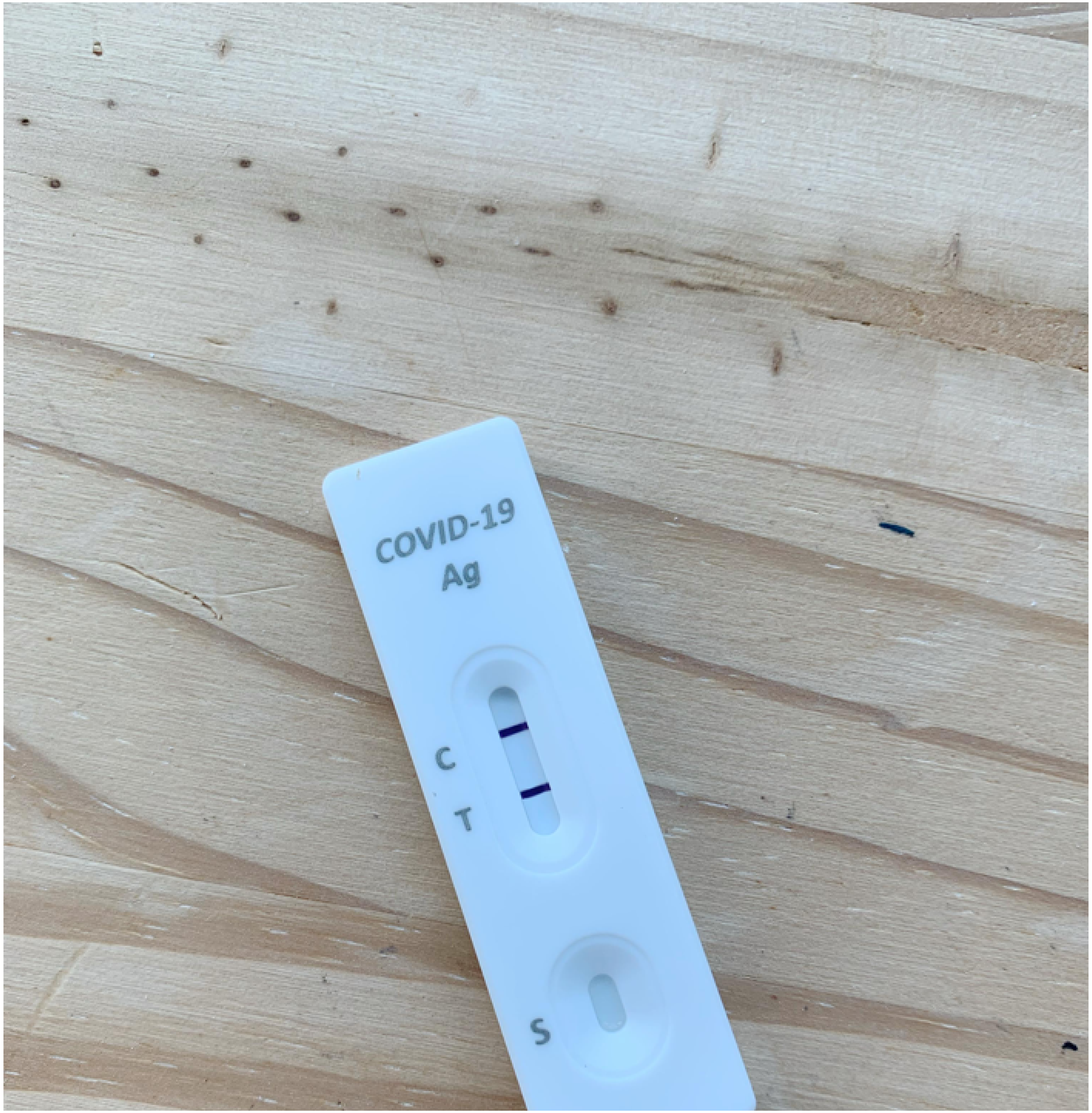
COVID-19
Ag
C
T
S

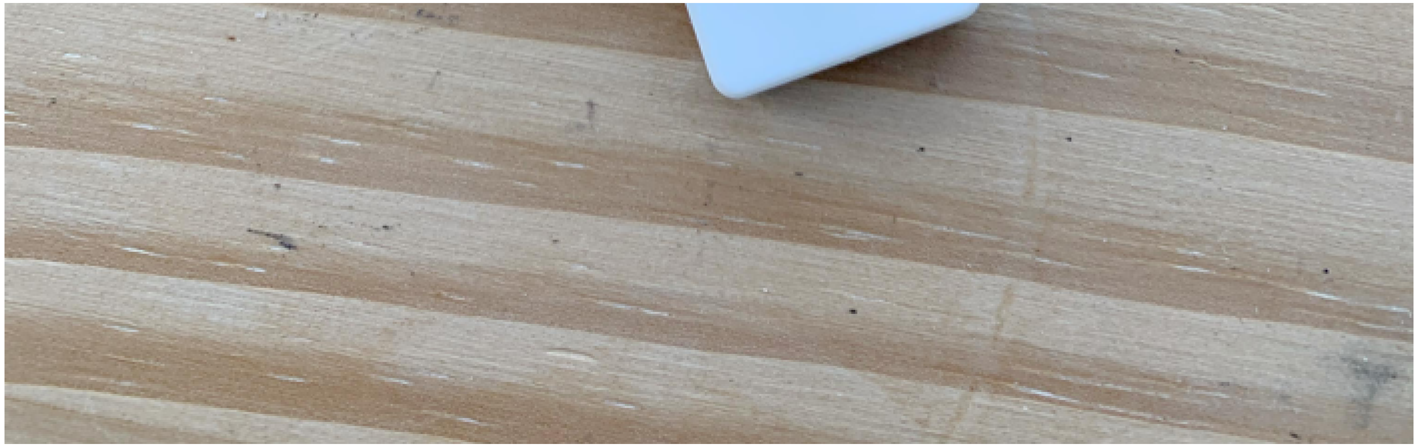

Say you did decide to do a RAT and the result appears as a strong positive line. Based on the information provided, please answer the following questions.

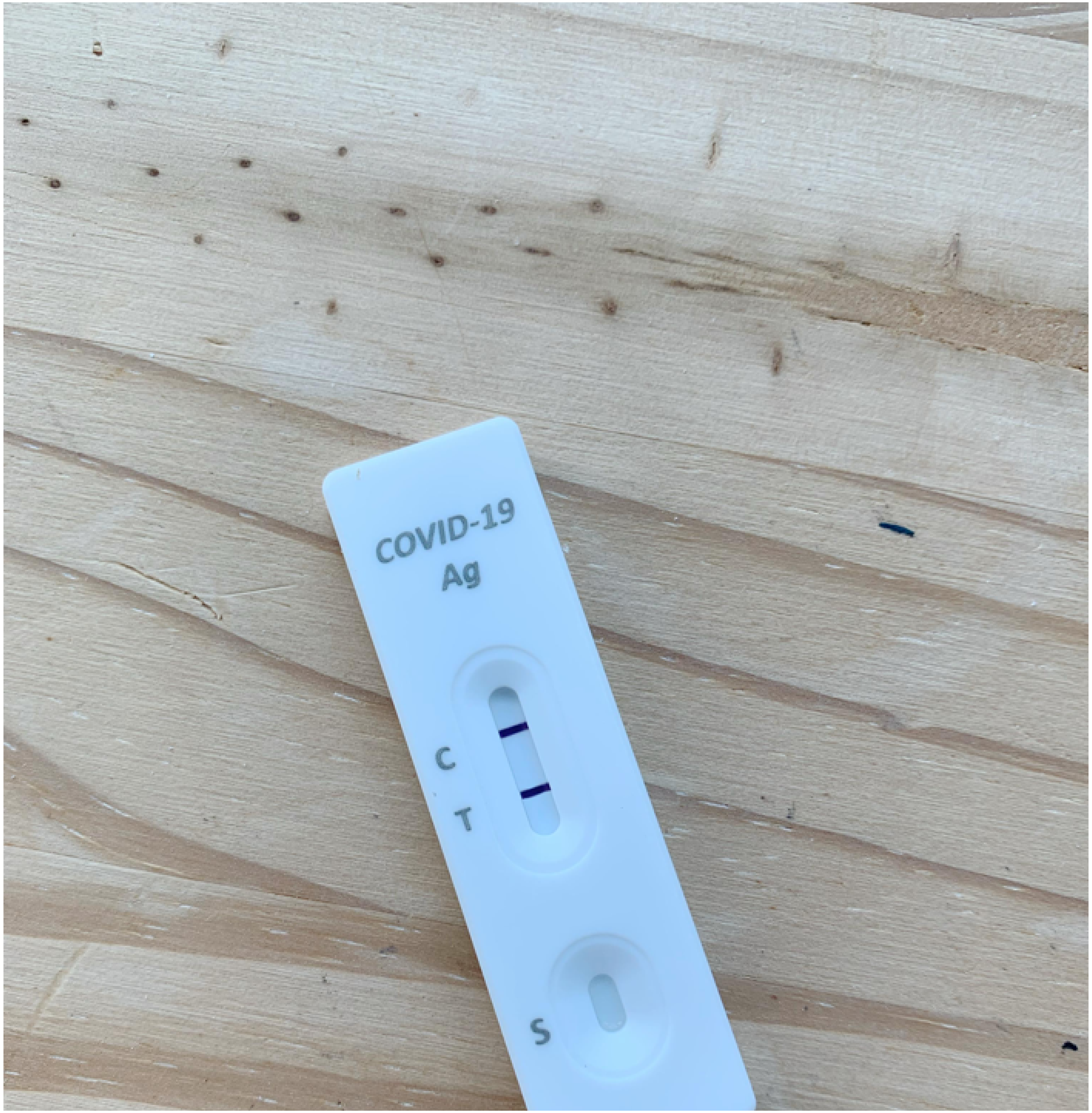
COVID-19
Ag
C
T
S

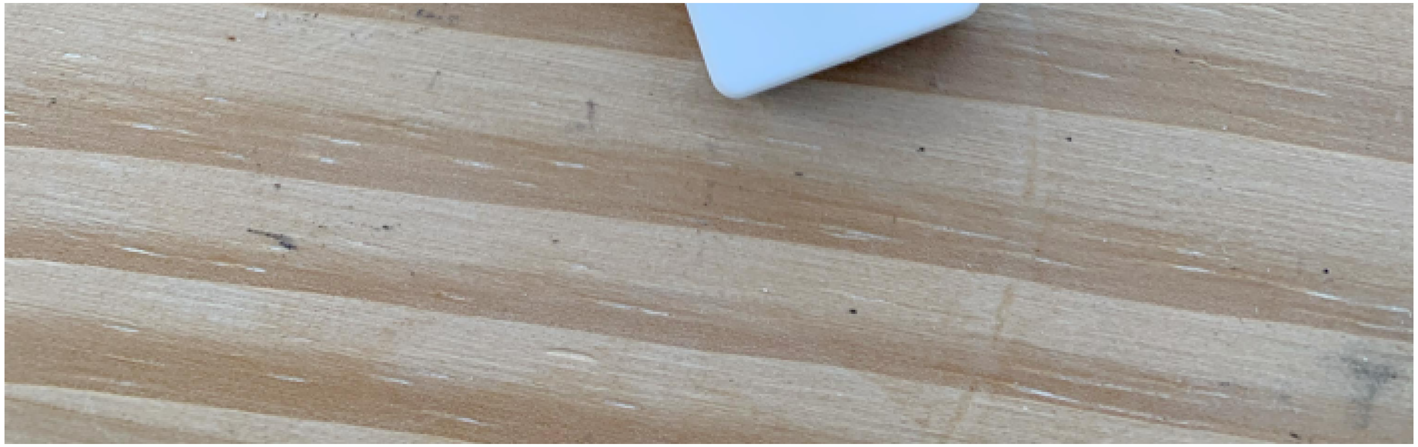

Would you report the results of your positive test?

- ◯ Yes
- ◯ No

What would you do next, in terms of staying at home?

- ◯ Stay at home without exception
- ◯ Stay at home except for shopping
- ◯ Stay at home except for work
- ◯ Stay at home except for shopping and work
- ◯ Continue to leave the house as normal

If you live with other people, would you:

- ◯ Isolate from other household members
- ◯ Not isolate from other household members
- ◯ [ ] Other
- ◯ Not Applicable (I live alone)

What other tests would you do in the next few days?

- ☐ Have 1 or more PCR tests
- ☐ Do further RAT(s)
- ☐ No further tests

What further actions would you take (select all that apply)?

- ☐ Avoid visiting people at higher risk of developing complications from COVID-19 (e.g. older people)
- ☐ Avoid crowds
- ☐ Keep 1.5m away from others
- ☐ Wash hands more often
- ☐ Wear a mask indoors when around others

Scenario 2/5:

You have COVID-19 and have been isolating at home. It is now day 4 since you first developed symptoms, and you have felt well since waking up today and no longer have symptoms. Would you do a RAT?

- ◯ Yes
- ◯ No

You do a RAT and the result appears as a strong positive line. Based on the information provided, please answer the following questions.

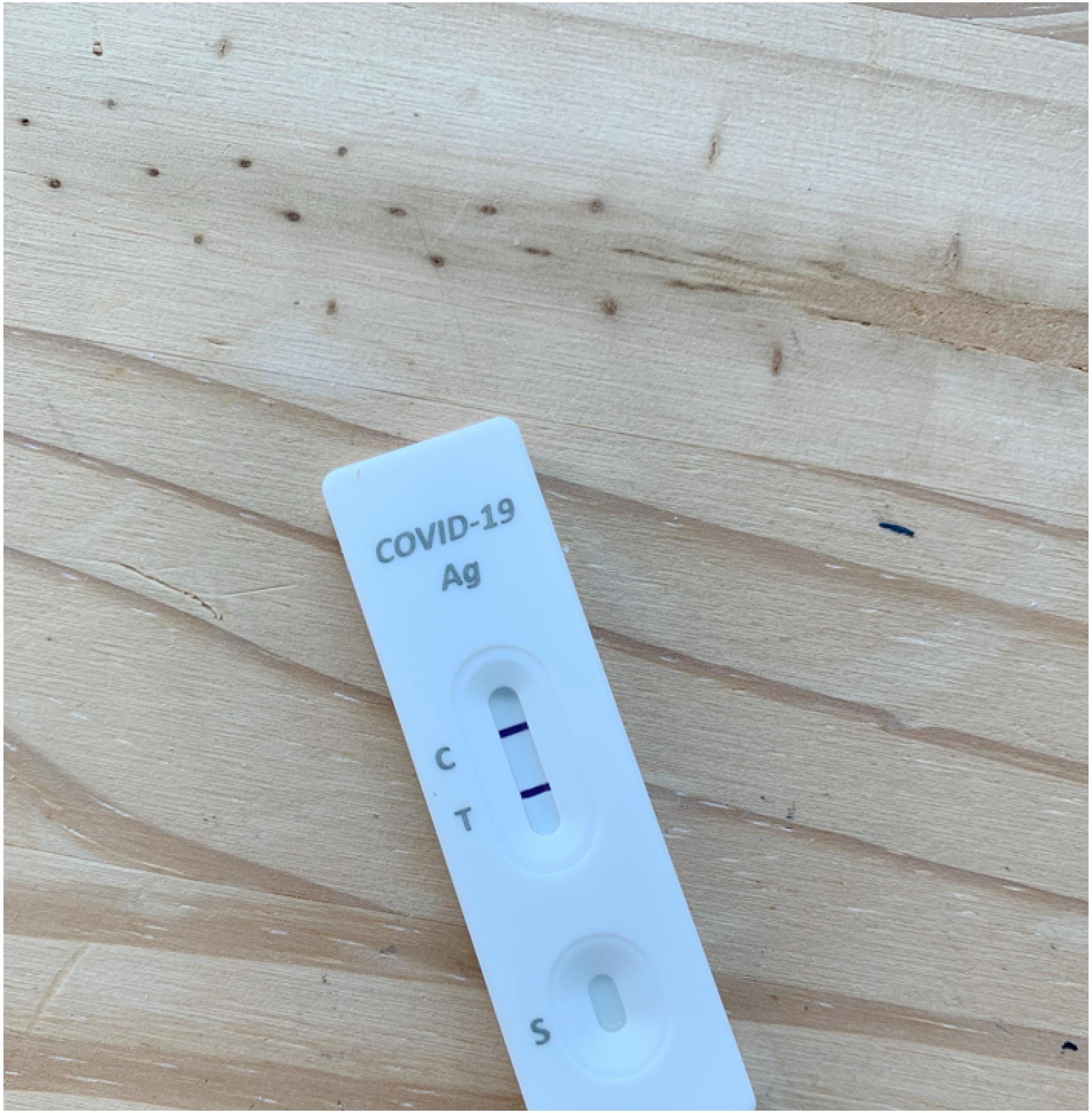
COVID-19
Ag
C
T
S

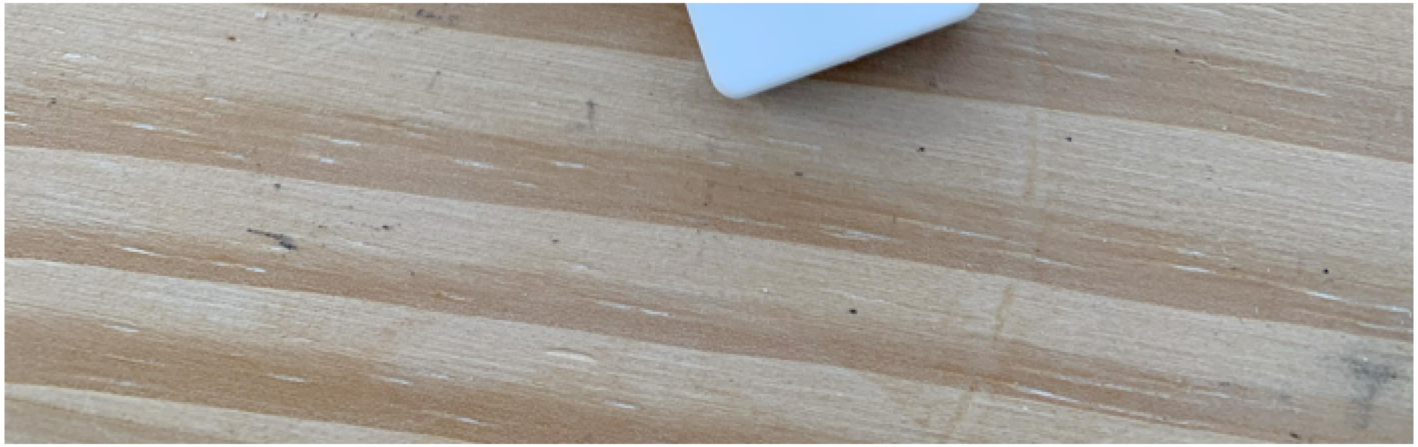

Say you did decide to do a RAT and the result appears as a strong positive line. Based on the information provided, please answer the following questions.

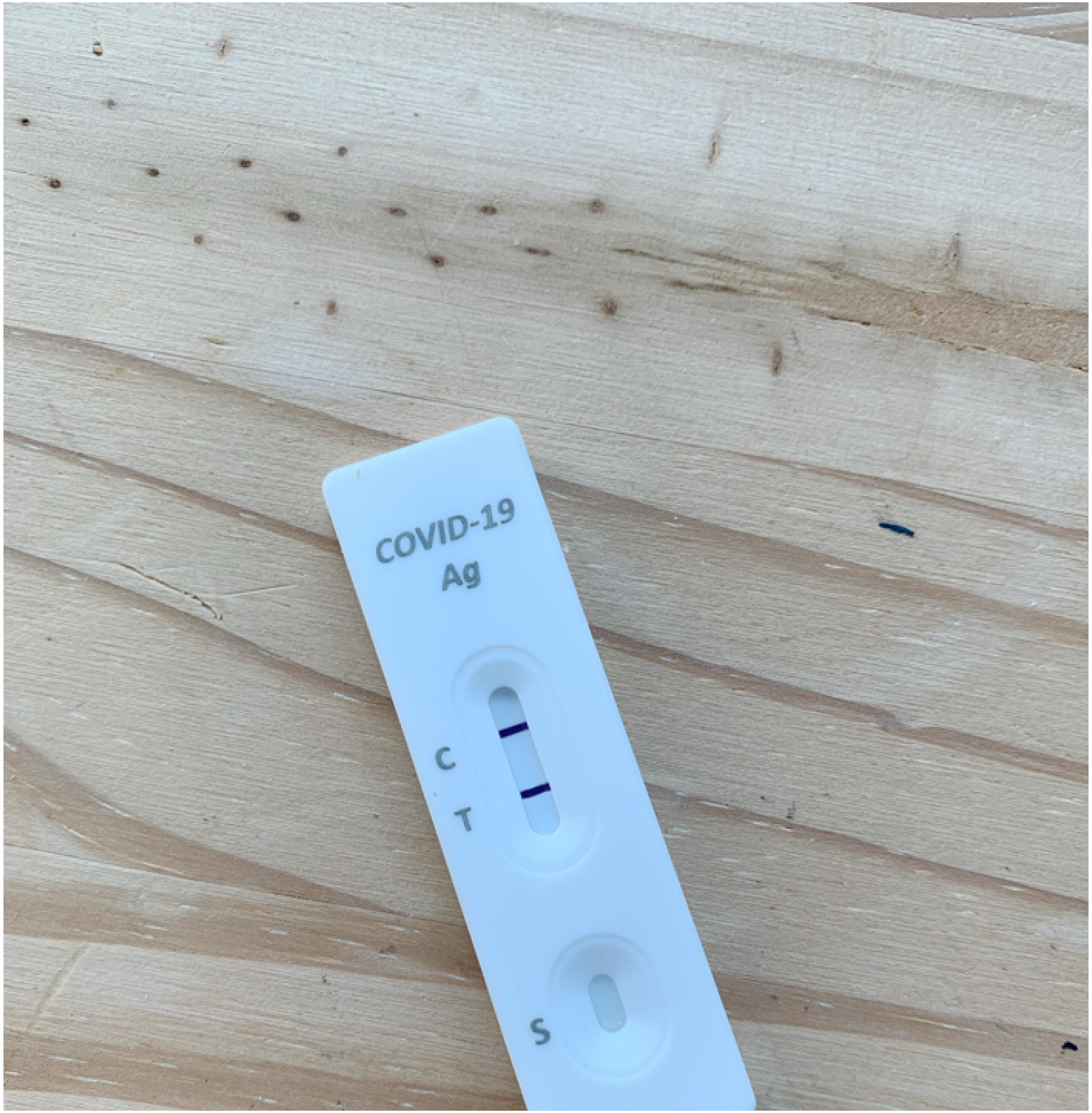
COVID-19
Ag
C
T
S

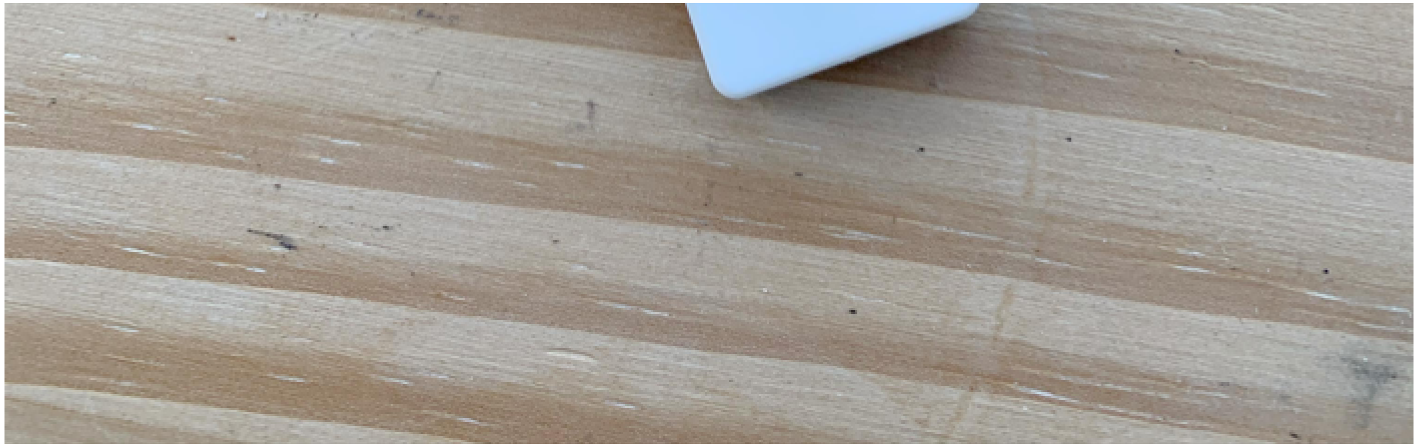

How likely do you think it is that you are still infectious?

- ◯ Very likely I am still infectious
- ◯ Likely I am still infectious
- ◯ I don’t know whether I am still infectious (neither likely nor unlikely)
- ◯ Unlikely I am still infectious
- ◯ Very unlikely I am still infectious

Now we would like you to use numbers to say how likely it is that you are still infectious. How likely do you think it is that you are still infectious? Please drag the slider to a number from 0% (no chance that I am infectious) to 100% (I am definitely infectious).

	No chance that I am infectious		Equal chance of being infectious or not being infectious		I am definitely infectious
	0	25	50	75	100
Chance of being infectious					

How confident are you in your response above?

	Not confident at all		Medium level of confidence		Absolutely confident
	0	25	50	75	100
Confidence Level					

What would you do next, in terms of staying at home for the next 24 hours?

- ◯ Stay at home without exception
- ◯ Stay at home except for shopping
- ◯ Stay at home except for work
- ◯ Stay at home except for shopping and work
- ◯ Stop self-isolating and leave your house

If you live with other people, would you:

- ◯ Isolate from other household members
- ◯ Not isolate from other household members
- ◯ [ ] Other
- ◯ Not Applicable (I live alone)

What other tests would you do in the next few days?

- ☐ Have 1 or more PCR tests
- ☐ Do further RAT(s) until negative
- ☐ No further tests

What further actions would you take (select all that apply)?

- ☐ Avoid visiting people at higher risk of developing complications from COVID-19 (e.g. older people)
- ☐ Avoid crowds
- ☐ Keep 1.5m away from others
- ☐ Wash hands more often
- ☐ Wear a mask indoors when around others

Scenario 3/5:
It is now Day 6 since you first developed symptoms. You have felt well since Day 4, with no symptoms. Would you do a RAT?

- ◯ Yes
- ◯ No

You do a RAT and the result appears as a faint line. Based on the information provided, please answer the following questions.

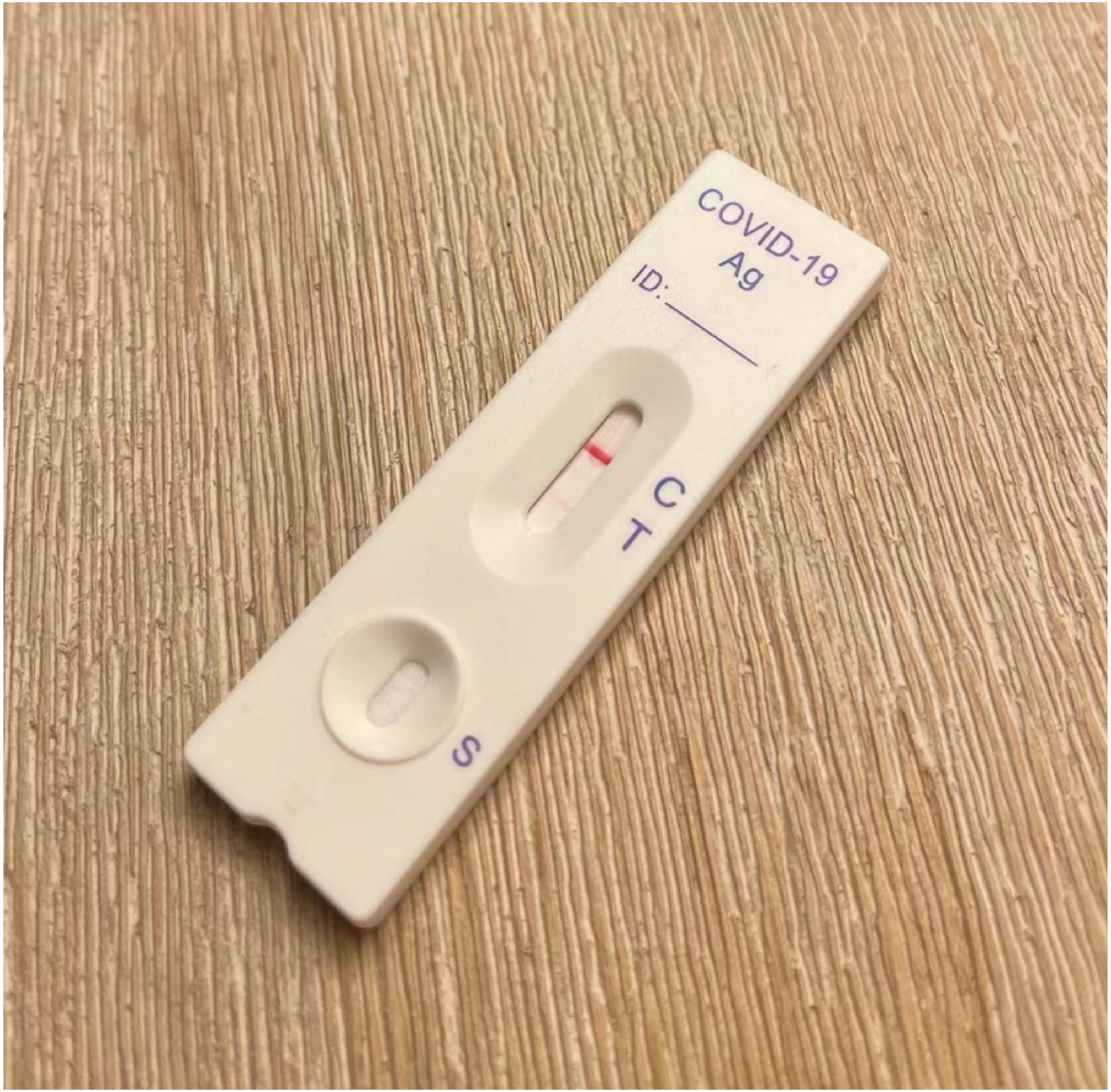
COVID-19
Ag
ID:
C
T
S

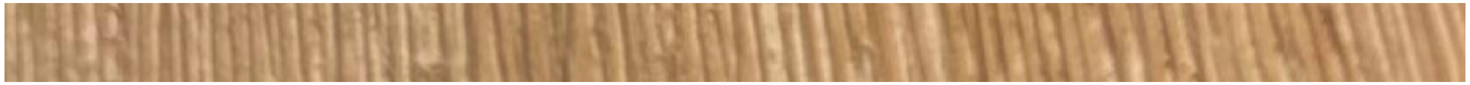

Say you did decide to do a RAT and the result appears as a faint line. Based on the information provided, please answer the following questions.

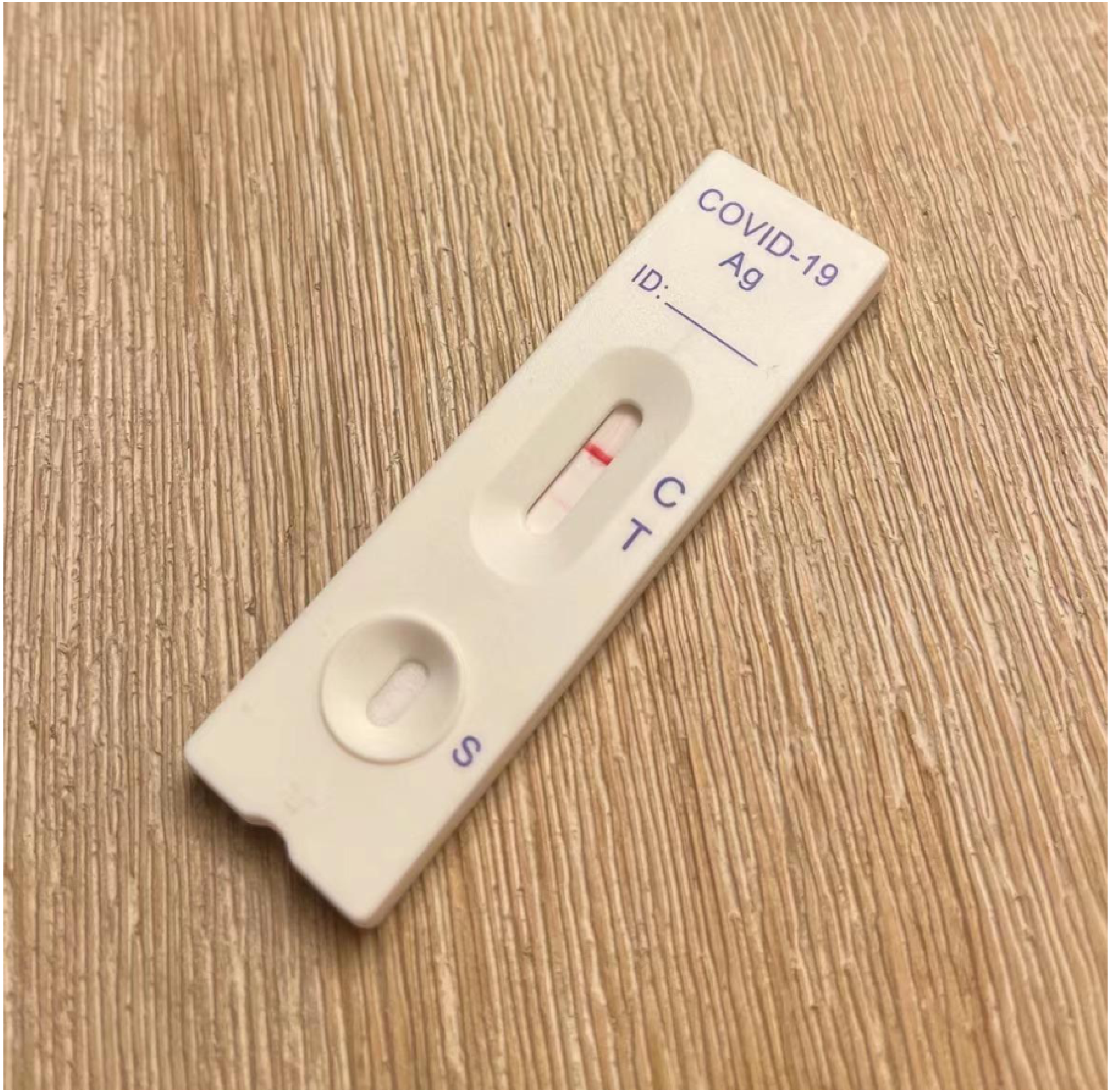
COVID-19
Ag
ID:
C
T
S

How likely do you think it is that you are still infectious?

- ◯ Very likely I am still infectious
- ◯ Likely I am still infectious
- ◯ I don’t know whether I am still infectious (neither likely nor unlikely)
- ◯ Unlikely I am still infectious
- ◯ Very unlikely I am still infectious

Now we would like you to use numbers to say how likely it is that you are still infectious. How likely do you think it is that you are still infectious? Please drag the slider to a number from 0% (no chance that I am infectious) to 100% (I am definitely infectious).

	No chance that I am infectious		Equal chance of being infectious or not being infectious		I am definitely infectious
	0	25	50	75	100
Chance of being infectious					

How confident are you in your response above?

	Not confident at all		Medium level of confidence		Absolutely confident
	0	25	50	75	100
Confidence Level					

What would you do next, in terms of staying at home?

- ◯ Stay at home without exception
- ◯ Stay at home except for shopping
- ◯ Stay at home except for work
- ◯ Stay at home except for shopping and work
- ◯ Continue to leave the house as normal

If you live with other people, would you:

- ◯ Isolate from other household members
- ◯ Not isolate from other household members
- ◯ ____ Other
- ◯ Not Applicable (I live alone)

What other tests would you do in the next few days?

- ☐ Have 1 or more PCR tests
- ☐ Do further RAT(s) until negative
- ☐ No further tests

What further actions would you take (select all that apply)?

- ☐ Avoid visiting people at higher risk of developing complications from COVID-19 (e.g. older people)
- ☐ Avoid crowds
- ☐ Keep 1.5m away from others
- ☐ Wash hands more often
- ☐ Wear a mask indoors when around others

Scenario 4/5:
Now imagine someone was staying at your home while you were sick with COVID-19. On Day 7 since you first developed symptoms, they wake up with a sore throat and runny nose. Would you advise them to do a RAT?

- ◯ Yes
- ◯ No

They follow your advice, do a RAT and the result is negative. Based on the information provided, please answer the following questions.

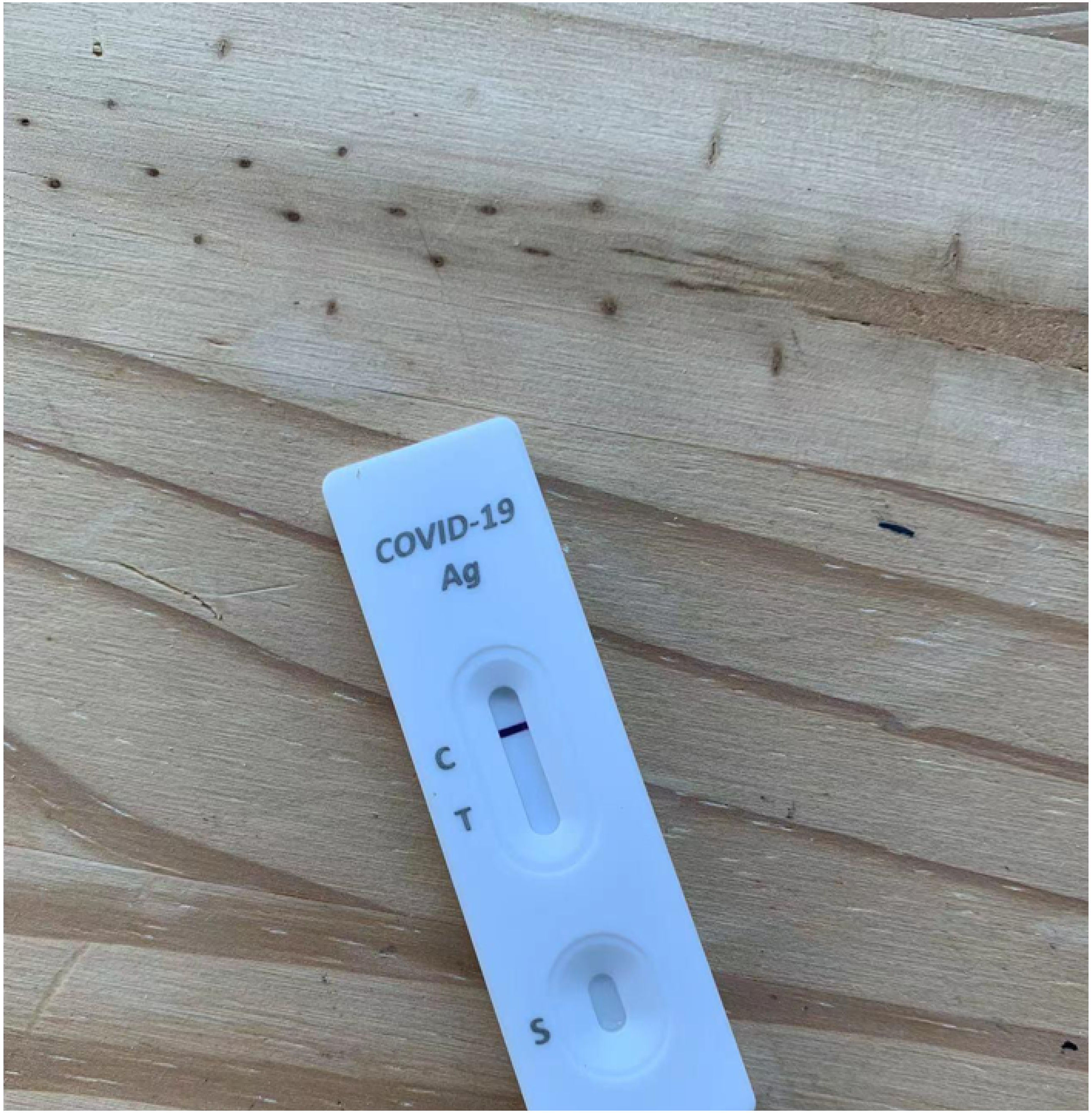
COVID-19
Ag
C
T
S

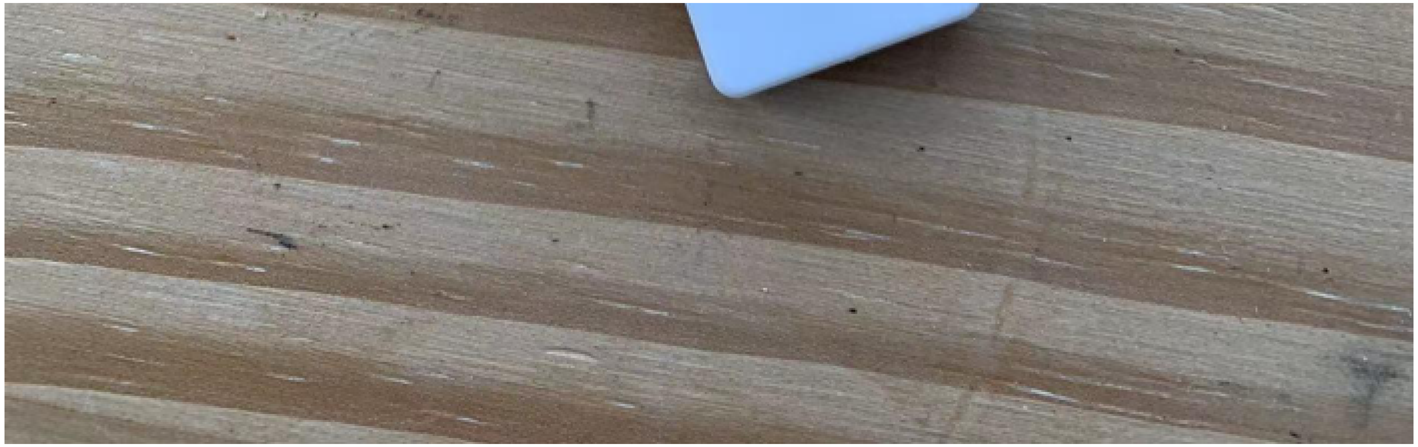

Say they did decide to do a RAT and the result is negative. Based on the information provided, please answer the following questions.

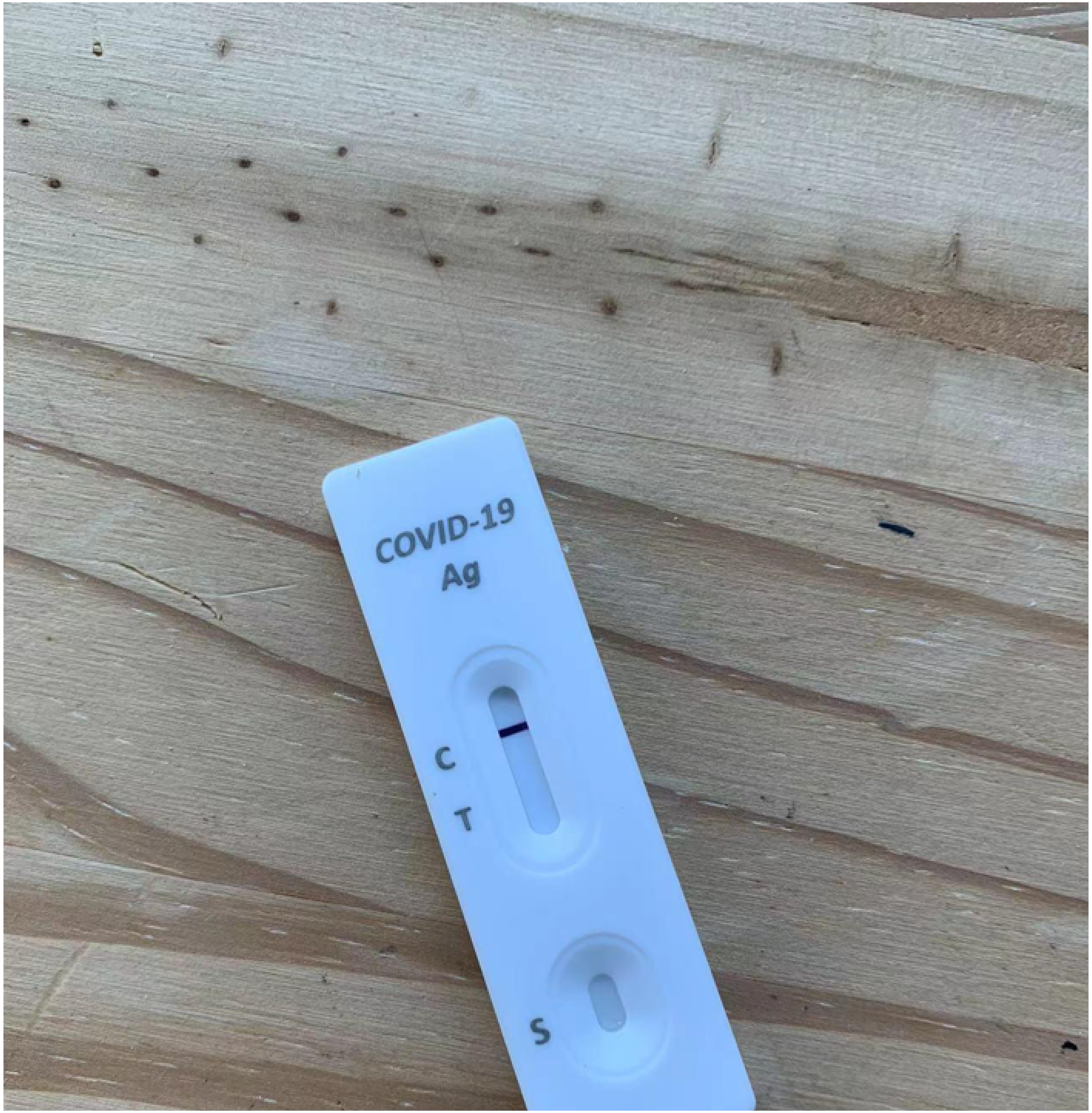
COVID-19
Ag
C
T
S

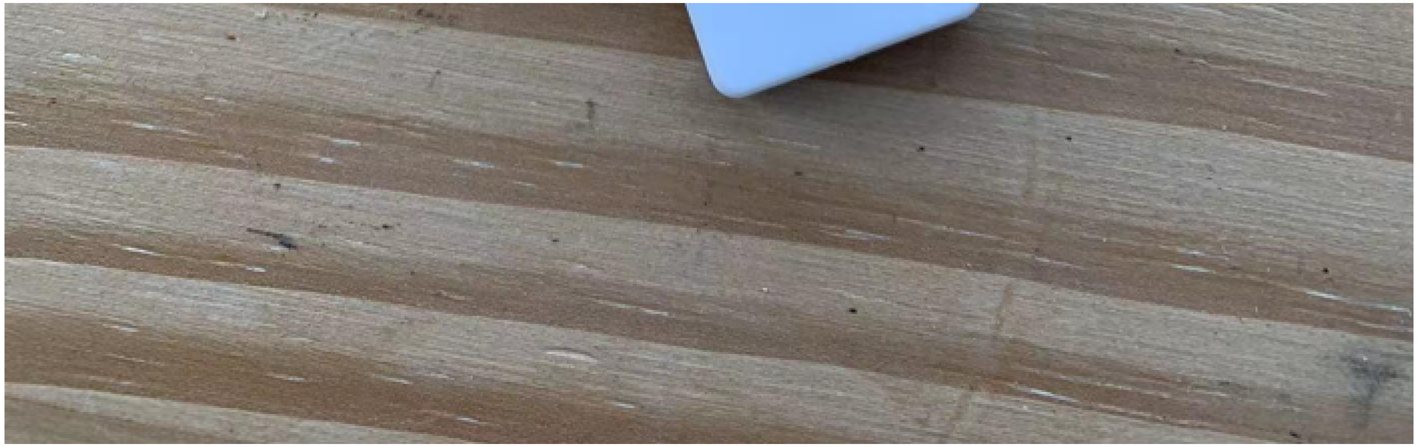

How likely do you think it is that they have COVID-19?

- Very likely
- Likely
- I don’t know
- Unlikely
- Very unlikely

Now we would like you to use numbers to say how likely it is that they have COVID-19 infection.

How likely do you think it is that they have COVID-19 infection? Please drag the slider to a number from 0% (no chance that they are infected) to 100% (they are definitely infected).

	No chance that they are infected		Equal chance of them being infected or not being infected		They are definitely infected
	0	25	50	75	100
Chance of being infectious					

How confident are you in your response above?

	Not confident at all		Medium level of confidence		Absolutely confident
	0	25	50	75	100
Confidence Level					

What advice would you give them to do next, in terms of staying at home?

- ◯ Stay at home without exception
- ◯ Stay at home except for shopping
- ◯ Stay at home except for work
- ◯ Stay at home except for shopping and work
- ◯ Continue to leave the house as normal

What other tests would you advise them to do in the next few days?

- ☐ Have 1 or more PCR tests
- ☐ Do further RAT(s)
- ☐ No further tests

What further actions would you advise them to take (select all that apply)?

- ☐ Avoid visiting people at higher risk of developing complications from COVID-19 (e.g. older people)
- ☐ Avoid crowds
- ☐ Keep 1.5m away from others
- ☐ Wash hands more often
- ☐ Wear a mask indoors when around others

Scenario 5/5:
Imagine it is now six months later and you have dinner at your friend's house with 9 other people. The dinner lasted about 3 hours. 2 days later your friend told you that they and 2 other people at the dinner party have tested positive for COVID-19. You are experiencing a sore throat and runny nose. Would you do a RAT?

- ◯ Yes

◯ No

You take a RAT but the result is negative. Based on the information provided, please answer the following questions.

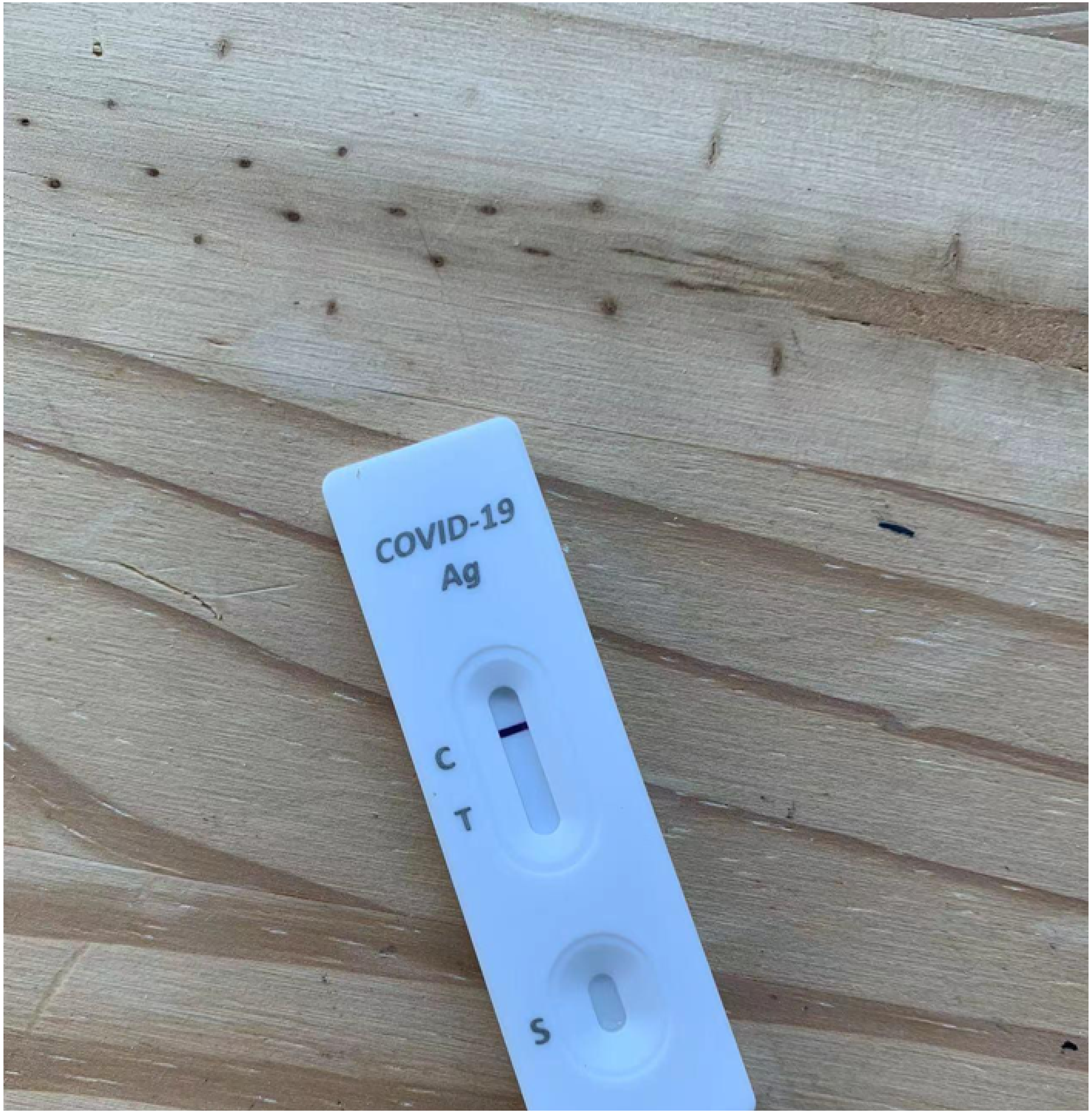
COVID-19
Ag
C
T
S

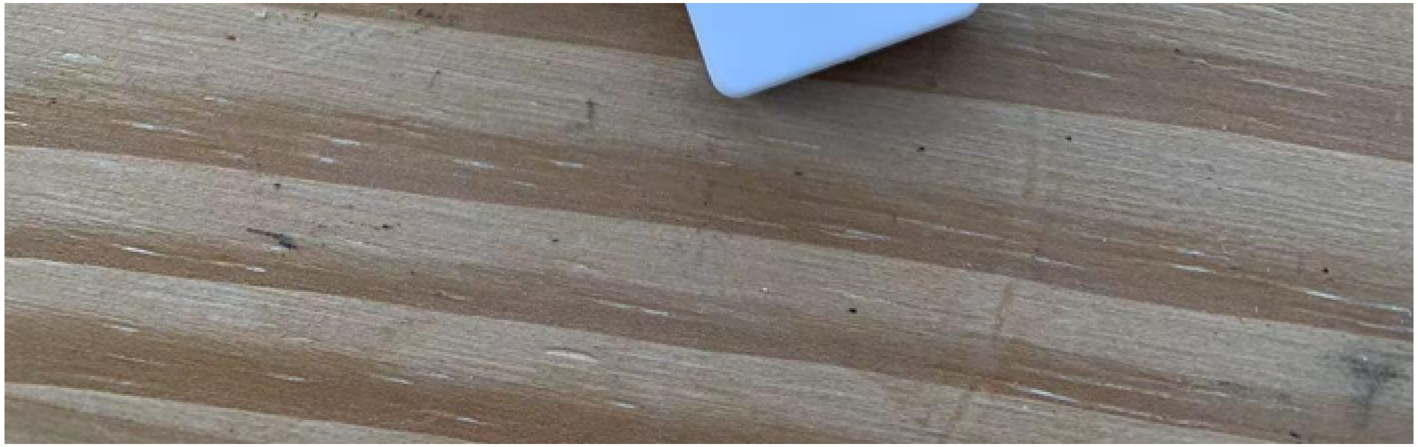

Say you did decide to do a RAT but the result is negative. Based on the information provided, please answer the following questions.

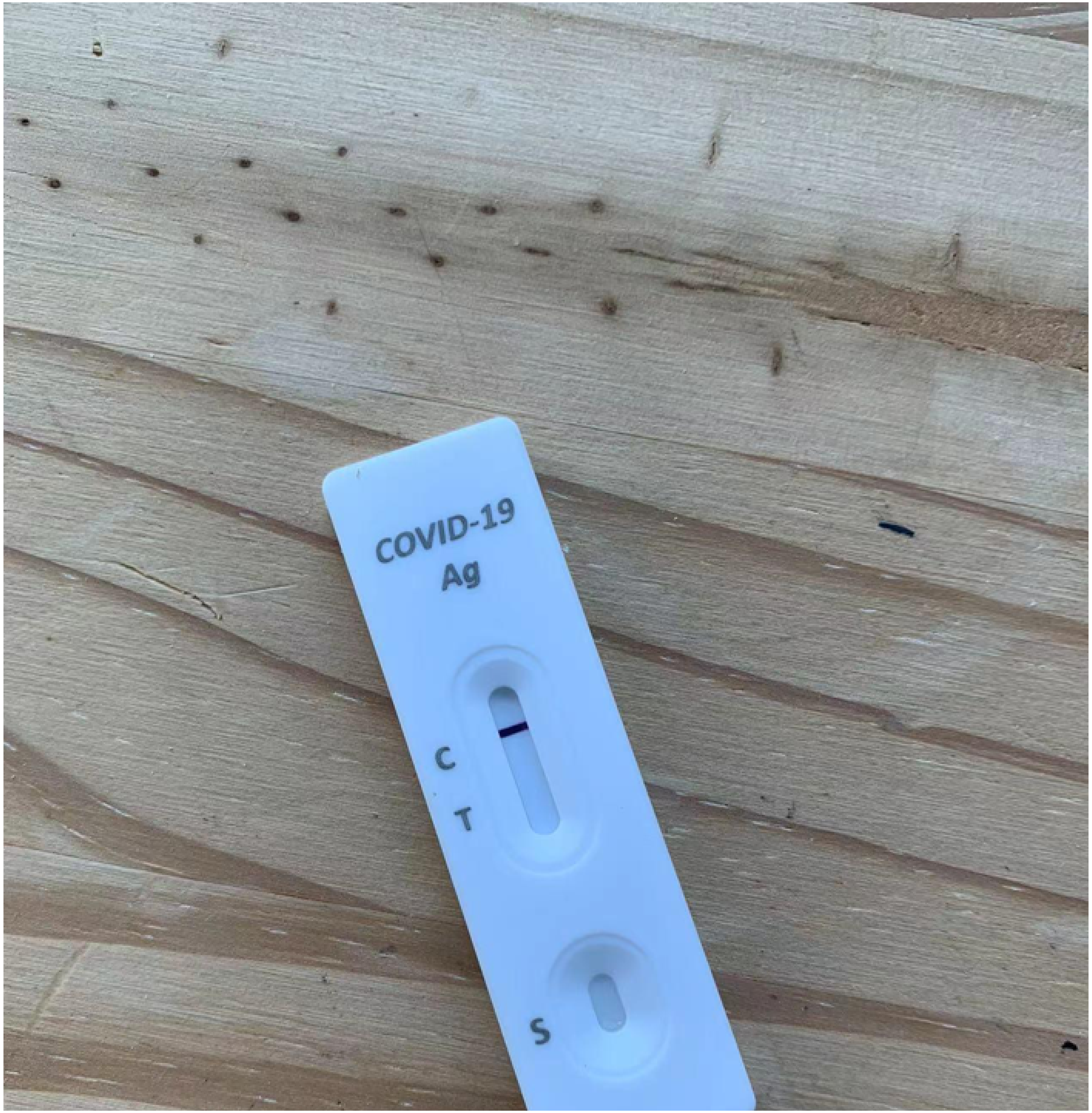
COVID-19
Ag
C
T
S

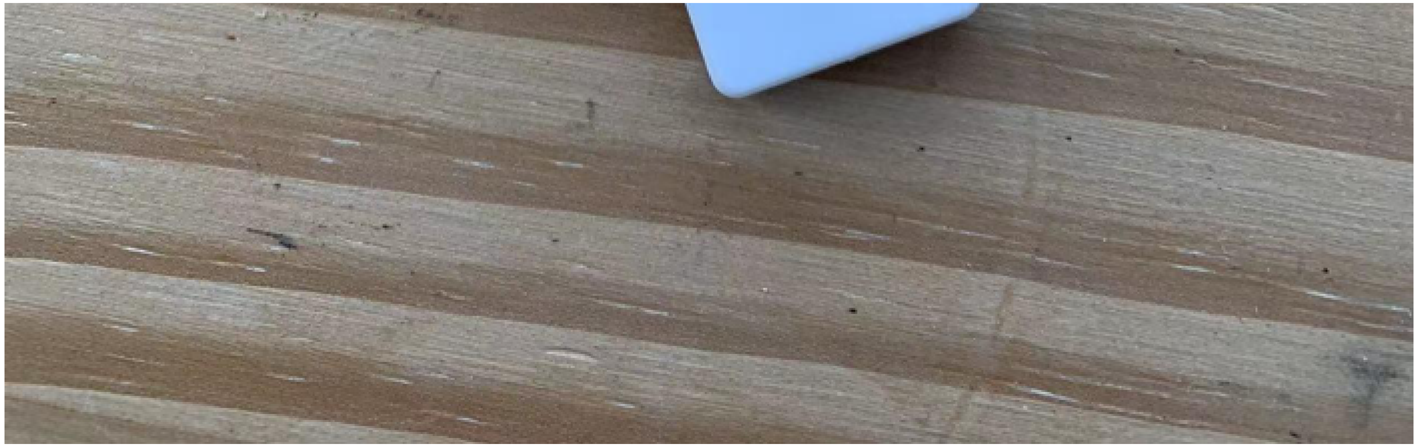

How likely do you think it is that you have COVID-19?

- Very likely
- Likely
- I don’t know
- Unlikely
- Very unlikely

Now we would like you to use numbers to say how likely it is that you have COVID-19 infection.
How likely do you think it is that you have COVID-19 infection? Please drag the slider to a number from 0% (no chance that I am infected) to 100% (I am definitely infected).

	No chance that I am infected		Equal chance of being infected or not being infected		I am definitely infected
	0	25	50	75	100
Chance of being infectious					

How confident are you in your response above?

	Not confident at all		Medium level of confidence		Absolutely confident
	0	25	50	75	100
Confidence Level					

What would you do next, in terms of staying at home?

- ◯ Stay at home without exception
- ◯ Stay at home except for shopping
- ◯ Stay at home except for work
- ◯ Stay at home except for shopping and work
- ◯ Continue to leave the house as normal

If you live with other people, would you:

- ◯ Isolate from other household members
- ◯ Not isolate from other household members
- ◯ [ ] Other
- ◯ Not Applicable (I live alone)

What other tests would you do in the next few days?

- ☐ Have 1 or more PCR tests
- ☐ Do further RAT(s)
- ☐ No further tests

What further actions would you take (select all that apply)?

- ☐ Avoid visiting people at higher risk of developing complications from COVID-19 (e.g. older people)
- ☐ Avoid crowds
- ☐ Keep 1.5m away from others
- ☐ Wash hands more often
- ☐ Wear a mask indoors when around others

Usual Care

A Rapid Antigen Test (RAT) is a quick home test to check if you may have COVID-19 and provides a result within 15 to 20 minutes.
The following information explains what to do if the RAT result is positive or negative and information about how accurate the test is. Please read this information and then answer the following questions.

.

RESULT INTERPRETATION

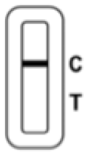

Negative

Only the control line (C) and no test line (T) appears. This means that no SARS-CoV-2 antigen was detected.
A negative test result indicates that you are unlikely to currently have COVID-19 disease. Continue to follow all applicable rules and protective measures when contacting with others. There may be an infection even if the test is negative. **If it is suspected, repeat the test after 1 - 2 days, as the coronavirus cannot be precisely detected in all phases of an infection.**

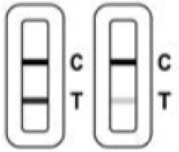

Positive

Both the control line (C) and test line (T) appears. This means that SARS-CoV-2 antigen was detected. ***NOTE: Any faint line in the test line region (T) should be considered positive.***
A positive test result means it is very likely you currently have COVID-19 disease. **Contact your State or Territory Coronavirus testing services for advice immediately.** Follow the local guidelines for self-isolation.

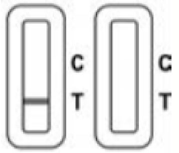

Invalid

Control line (C) fails to appear. Not enough specimen volume or incorrect operation are the likely reasons for an invalid result. Review the instructions again and repeat the test with a new cassette. If the test results remain invalid, contact your State or Territory Coronavirus testing services.

Clinical Sensitivity, Specificity and Accuracy

Performance of the SARS-CoV-2 Antigen Rapid Test was established with 605 nasal swabs collected from individuals who were suspected of COVID-19. The results show that the Sensitivity is 97.1% (165/170), Specificity is 99.5% (433/435) and an Overall Accuracy is 98.8% (598/605).

Now that you have read the RAT information, please answer the following questions.

What is the sensitivity of the RAT as a %?

Out of 100 people who have COVID-19, how many would test positive on the RAT?

How confident are you in your response above?

	Not confident at all		Medium level of confidence		Absolutely confident
	0	25	50	75	100
Confidence Level					

What is the specificity of the RAT as a %?

Out of 100 people who do not have COVID-19, how many would test negative on the RAT?

How confident are you in your response above?

	Not confident at all		Medium level of confidence		Absolutely confident
	0	25	50	75	100
Confidence Level					

How easy or difficult was it to read the information about interpreting RAT results?

- ◯ Very Easy
- ◯ Easy
- ◯ Neutral

- Difficult
- Very difficult

The information helped me know what to do if test result is positive.

- Strongly agree
- Agree
- Neutral
- Disagree
- Strongly disagree

The information helped me know what to do if test result is negative.

- Strongly agree
- Agree
- Neutral
- Disagree
- Strongly disagree

We will now present five different scenarios relating to COVID-19. Based on the scenarios, we will require you to answer how likely it is that you (or in one scenario

member of your household) has COVID-19 infection, and about specific actions you would take to prevent onward spread of infection.

Scenario 1/5:
Imagine you have been unwell with symptoms including headache, sore throat, fever, runny nose, and loss of taste and smell. Would you do a RAT?

- ◯ Yes
- ◯ No

You do a RAT and the result appears as a strong positive line. Based on the information provided, please answer the following questions.

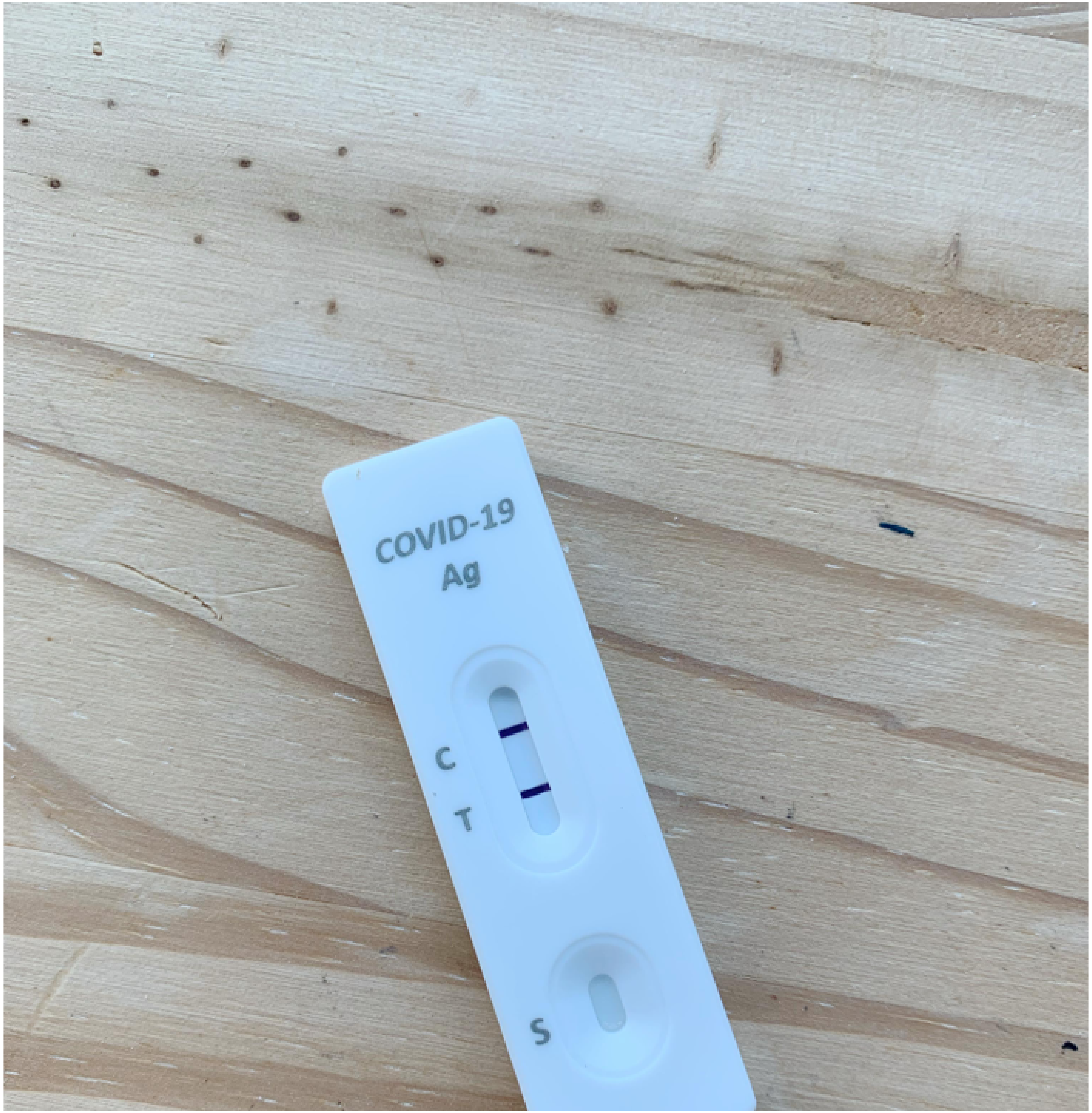
COVID-19
Ag
C
T
S

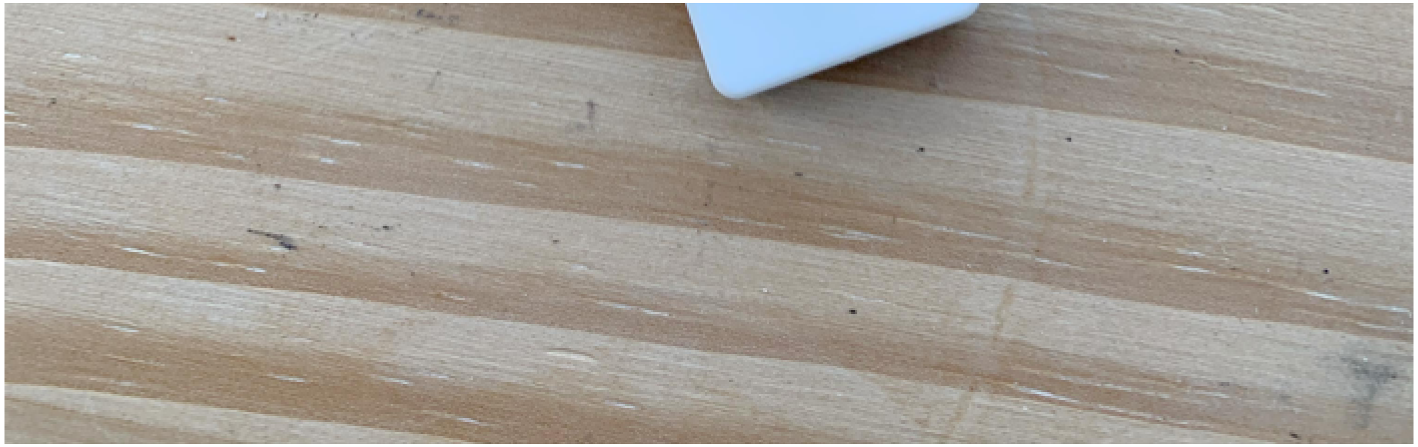

Say you did decide to do a RAT and the result appears as a strong positive line. Based on the information provided, please answer the following questions.

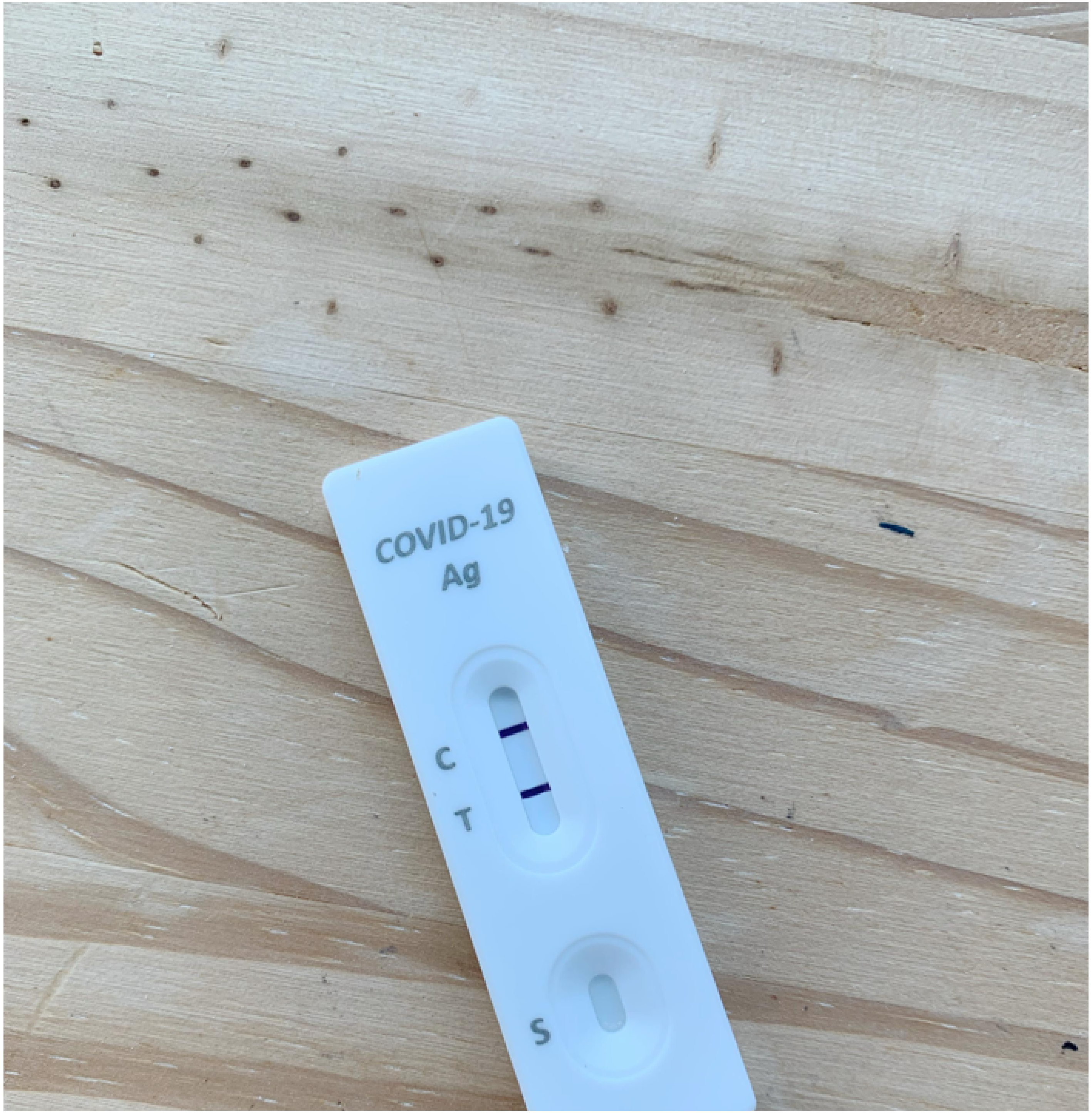
COVID-19
Ag
C
T
S

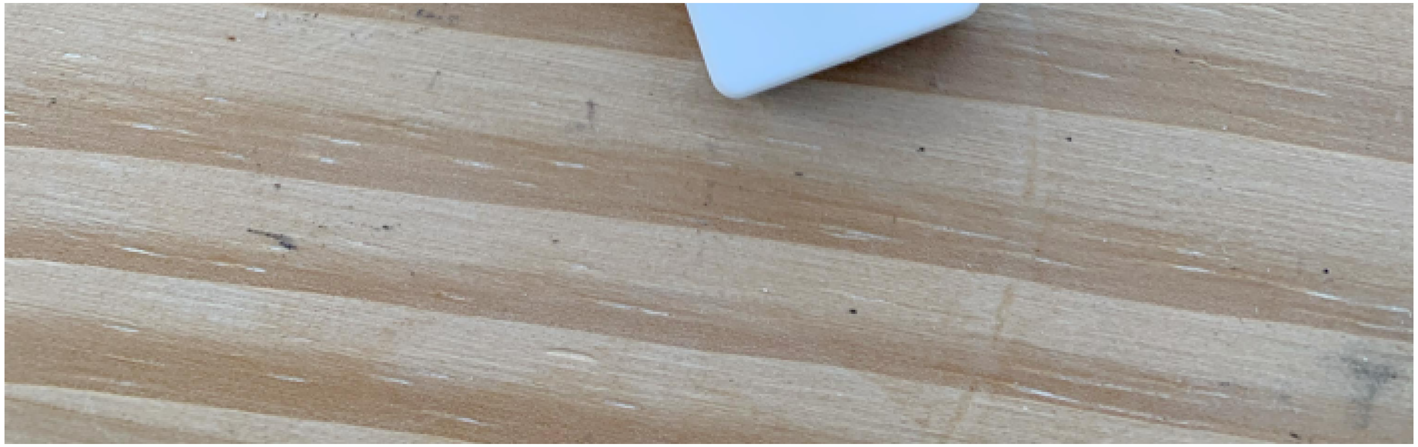

Would you report the results of your positive test?

- ◯ Yes
- ◯ No

What would you do next, in terms of staying at home?

- ◯ Stay at home without exception
- ◯ Stay at home except for shopping
- ◯ Stay at home except for work
- ◯ Stay at home except for shopping and work
- ◯ Continue to leave the house as normal

If you live with other people, would you:

- ◯ Isolate from other household members
- ◯ Not isolate from other household members
- ◯ [ ] Other
- ◯ Not Applicable (I live alone)

What other tests would you do in the next few days?

- ☐ Have 1 or more PCR tests
- ☐ Do further RAT(s)
- ☐ No further tests

What further actions would you take (select all that apply)?

- ☐ Avoid visiting people at higher risk of developing complications from COVID-19 (e.g. older people)
- ☐ Avoid crowds
- ☐ Keep 1.5m away from others
- ☐ Wash hands more often
- ☐ Wear a mask indoors when around others

Scenario 2/5:
You have COVID-19 and have been isolating at home. It is now day 4 since you first developed symptoms, and you have felt well since waking up today and no longer have symptoms. Would you do a RAT?

- Yes
- No

You do a RAT and the result appears as a strong positive line. Based on the information provided, please answer the following questions.

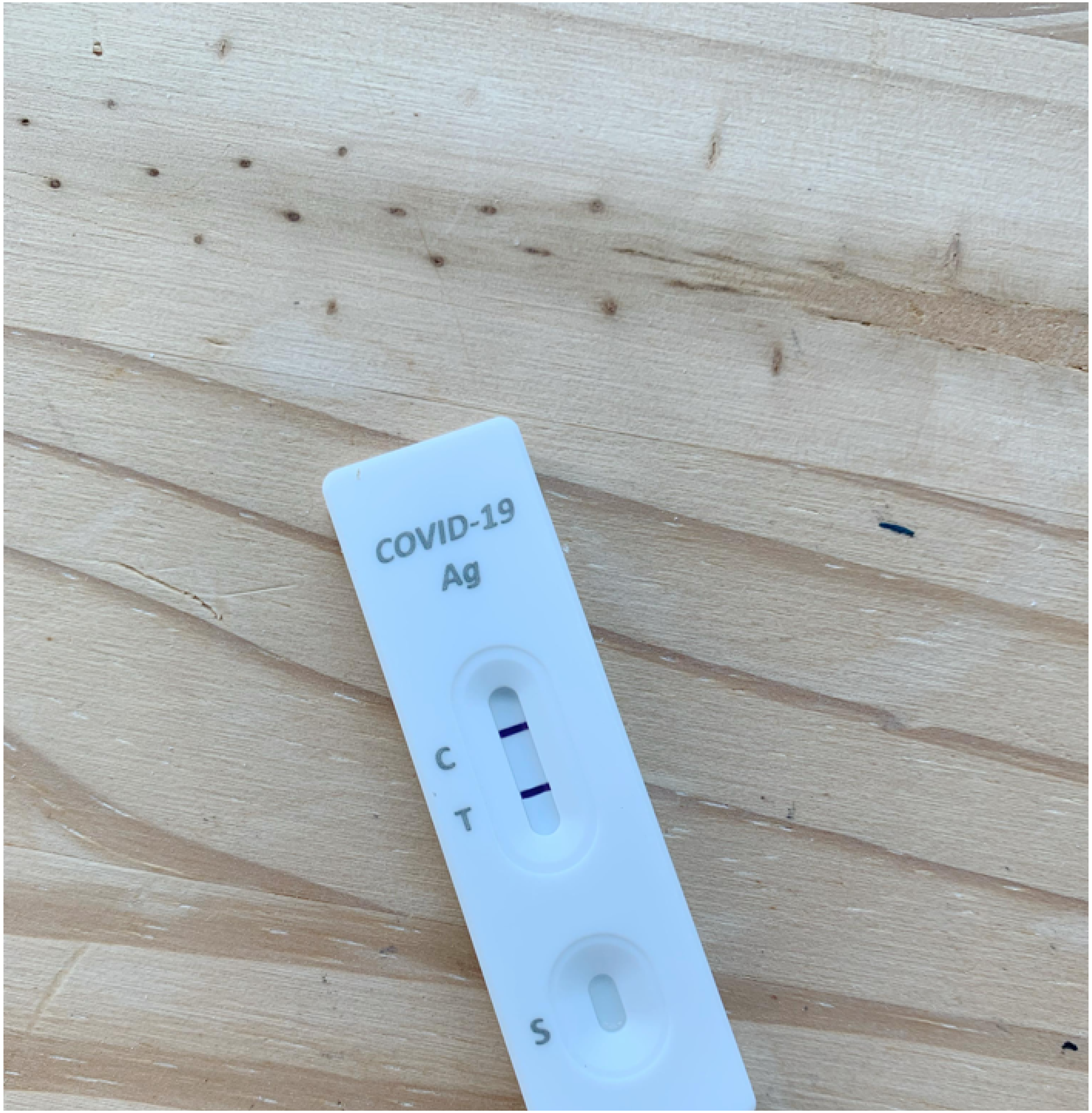
COVID-19
Ag
C
T
S

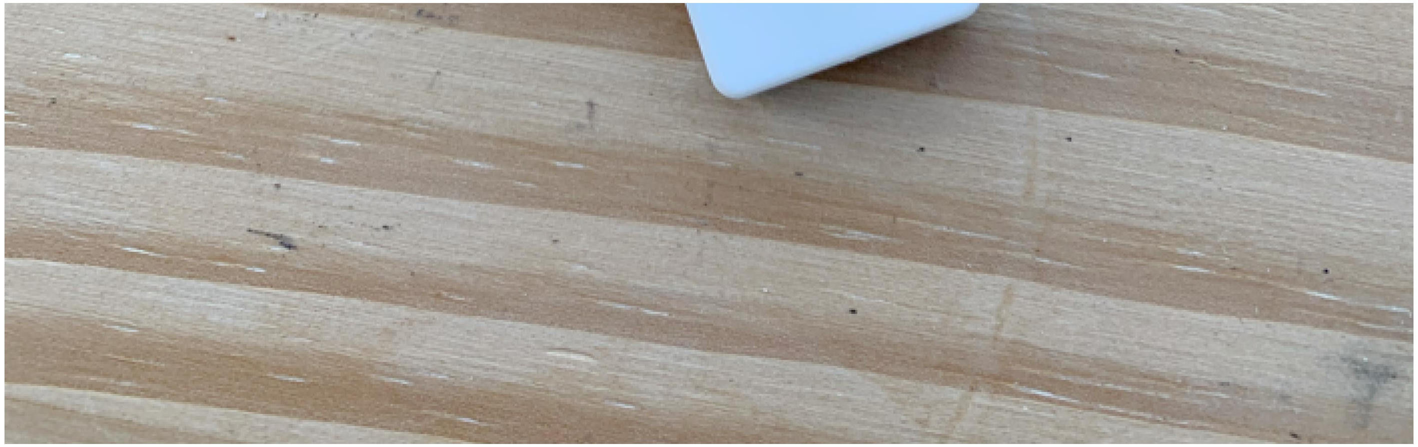

Say you did decide to do a RAT and the result appears as a strong positive line. Based on the information provided, please answer the following questions.

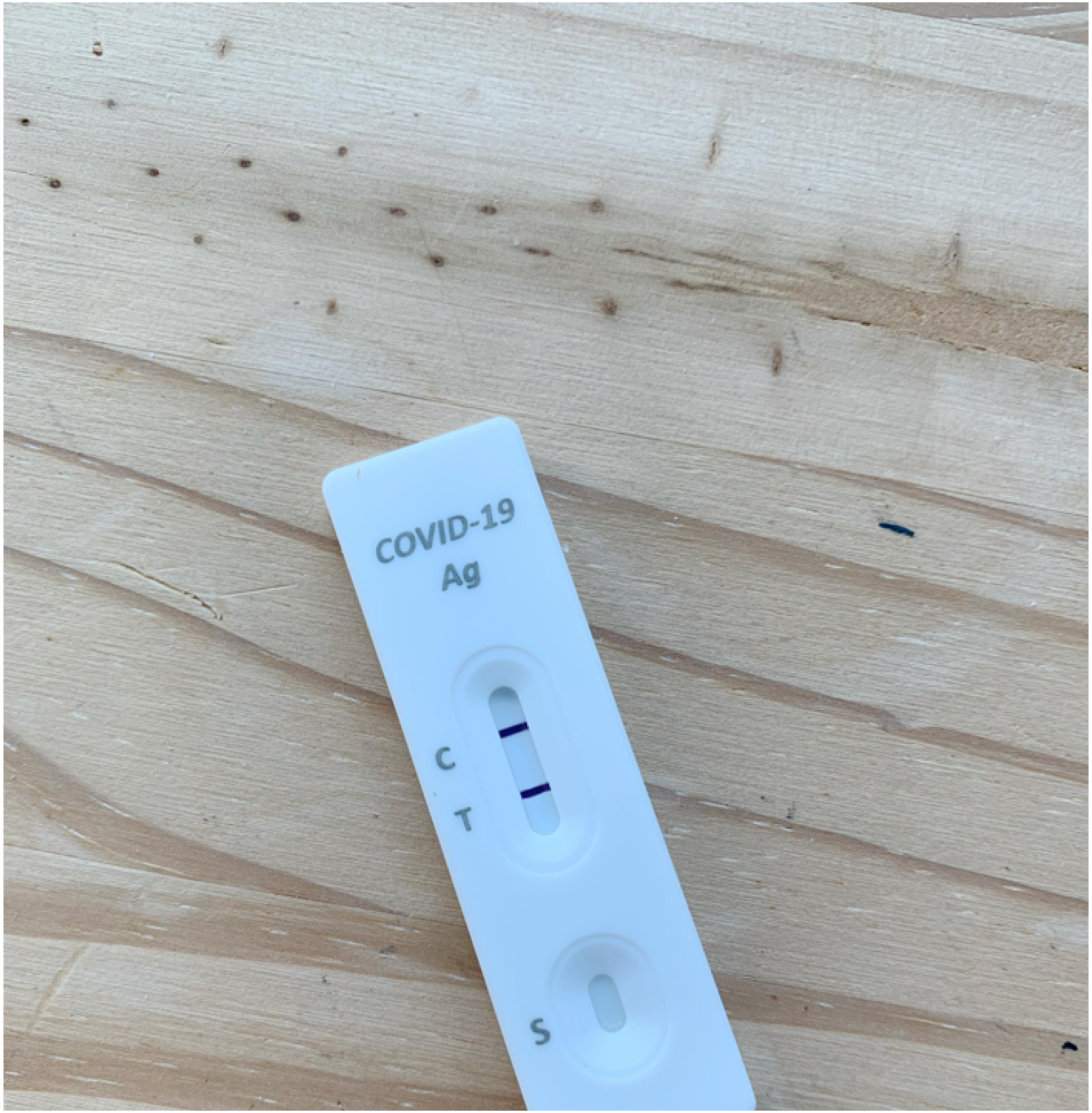
COVID-19
Ag
C
T
S

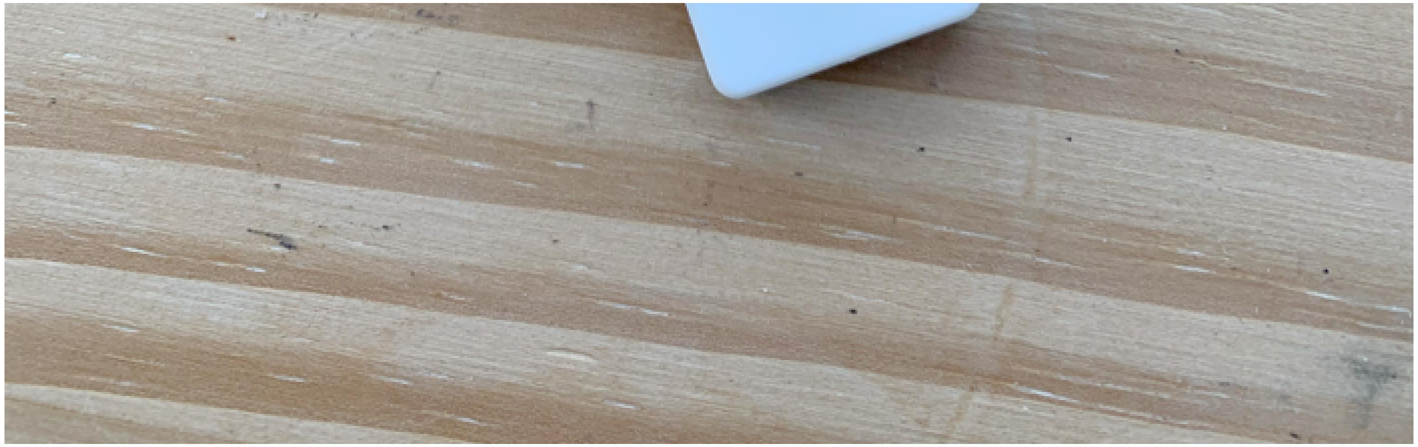

How likely do you think it is that you are still infectious?

- Very likely I am still infectious
- Likely I am still infectious
- I don’t know whether I am still infectious (neither likely nor unlikely)
- Unlikely I am still infectious
- Very unlikely I am still infectious

Now we would like you to use numbers to say how likely it is that you are still infectious. How likely do you think it is that you are still infectious? Please drag the slider to a number from 0% (no chance that I am infectious) to 100% (I am definitely infectious).

	No chance that I am infectious		Equal chance of being infectious or not being infectious		I am definitely infectious
	0	25	50	75	100
Chance of being infectious					

How confident are you in your response above?

	Not confident at all		Medium level of confidence		Absolutely confident
	0	25	50	75	100
Confidence Level					

What would you do next, in terms of staying at home for the next 24 hours?

- ◯ Stay at home without exception
- ◯ Stay at home except for shopping
- ◯ Stay at home except for work
- ◯ Stay at home except for shopping and work
- ◯ Stop self-isolating and leave your house

If you live with other people, would you:

- ◯ Isolate from other household members
- ◯ Not isolate from other household members
- ◯ [ ] Other
- ◯ Not Applicable (I live alone)

What other tests would you do in the next few days?

- ☐ Have 1 or more PCR tests
- ☐ Do further RAT(s) until negative
- ☐ No further tests

What further actions would you take (select all that apply)?

- ☐ Avoid visiting people at higher risk of developing complications from COVID-19 (e.g. older people)
- ☐ Avoid crowds
- ☐ Keep 1.5m away from others
- ☐ Wash hands more often
- ☐ Wear a mask indoors when around others

Scenario 3/5:
It is now Day 6 since you first developed symptoms. You have felt well since Day 4, with no symptoms. Would you do a RAT?

- ◯ Yes
- ◯ No

You do a RAT and the result appears as a faint line. Based on the information provided, please answer the following questions.

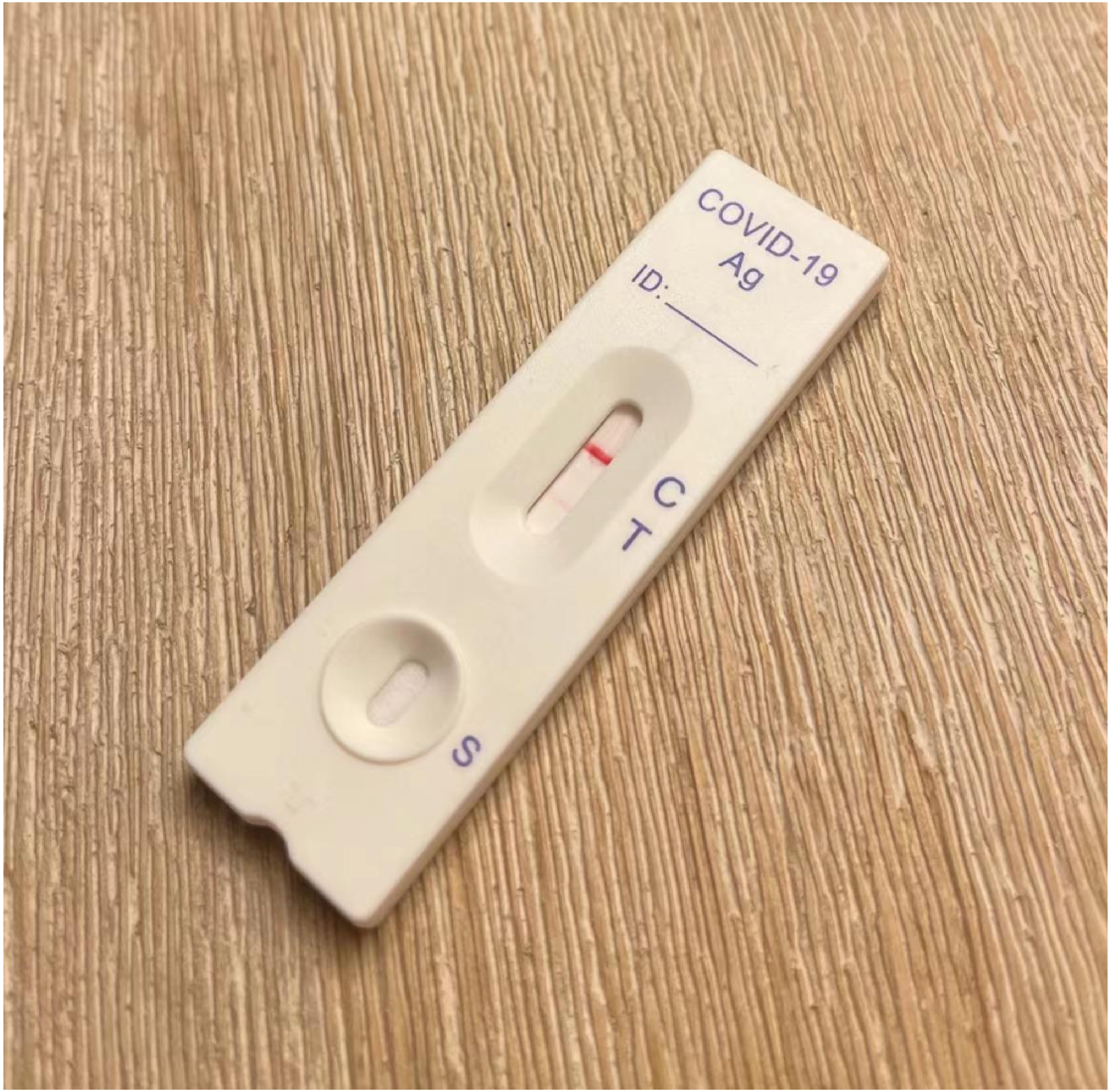
COVID-19
Ag
ID:
C
T
S

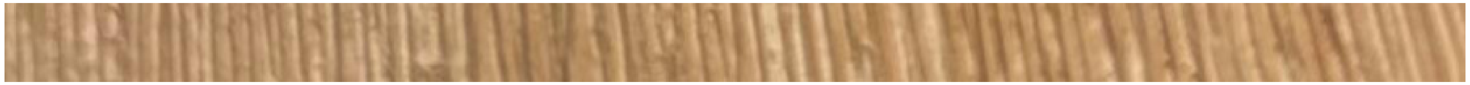

Say you did decide to do a RAT and the result appears as a faint line. Based on the information provided, please answer the following questions.

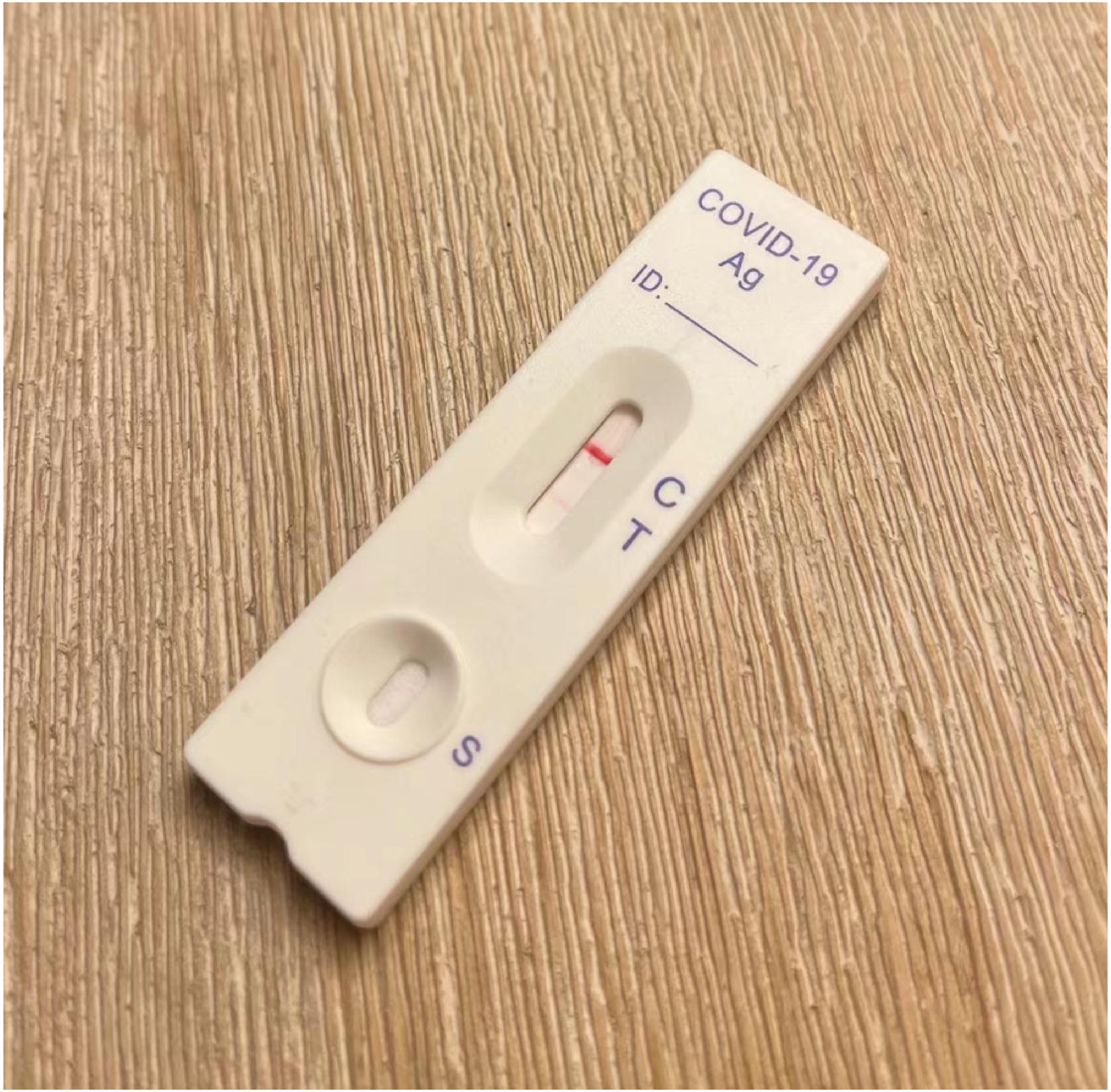
COVID-19
Ag
ID:
C
T
S

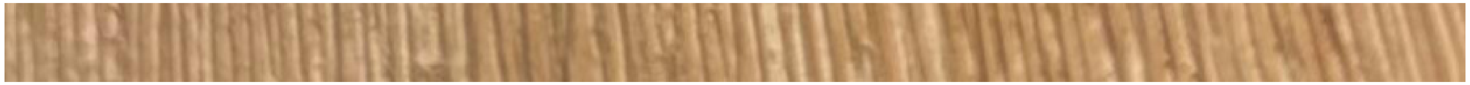

How likely do you think it is that you are still infectious?

- ◯ Very likely I am still infectious
- ◯ Likely I am still infectious
- ◯ I don’t know whether I am still infectious (neither likely nor unlikely)
- ◯ Unlikely I am still infectious
- ◯ Very unlikely I am still infectious

Now we would like you to use numbers to say how likely it is that you are still infectious. How likely do you think it is that you are still infectious? Please drag the slider to a number from 0% (no chance that I am infectious) to 100% (I am definitely infectious).

	No chance that I am infectious		Equal chance of being infectious or not being infectious		I am definitely infectious
	0	25	50	75	100
Chance of being infectious					

How confident are you in your response above?

	Not confident at all		Medium level of confidence		Absolutely confident
	0	25	50	75	100
Confidence Level					

What would you do next, in terms of staying at home?

- ◯ Stay at home without exception
- ◯ Stay at home except for shopping
- ◯ Stay at home except for work
- ◯ Stay at home except for shopping and work
- ◯ Continue to leave the house as normal

If you live with other people, would you:

- ◯ Isolate from other household members
- ◯ Not isolate from other household members
- ◯ [ ] Other
- ◯ Not Applicable (I live alone)

What other tests would you do in the next few days?

- [ ] Have 1 or more PCR tests
- [ ] Do further RAT(s) until negative
- [ ] No further tests

What further actions would you take (select all that apply)?

- [ ] Avoid visiting people at higher risk of developing complications from COVID-19 (e.g. older people)
- [ ] Avoid crowds
- [ ] Keep 1.5m away from others
- [ ] Wash hands more often
- [ ] Wear a mask indoors when around others

Scenario 4/5:
Now imagine someone was staying at your home while you were sick with COVID-19. On Day 7 since you first developed symptoms, they wake up with a sore throat and runny nose. Would you advise them to do a RAT?

- ○ Yes
- ○ No

They follow your advice, do a RAT and the result is negative. Based on the information provided, please answer the following questions.

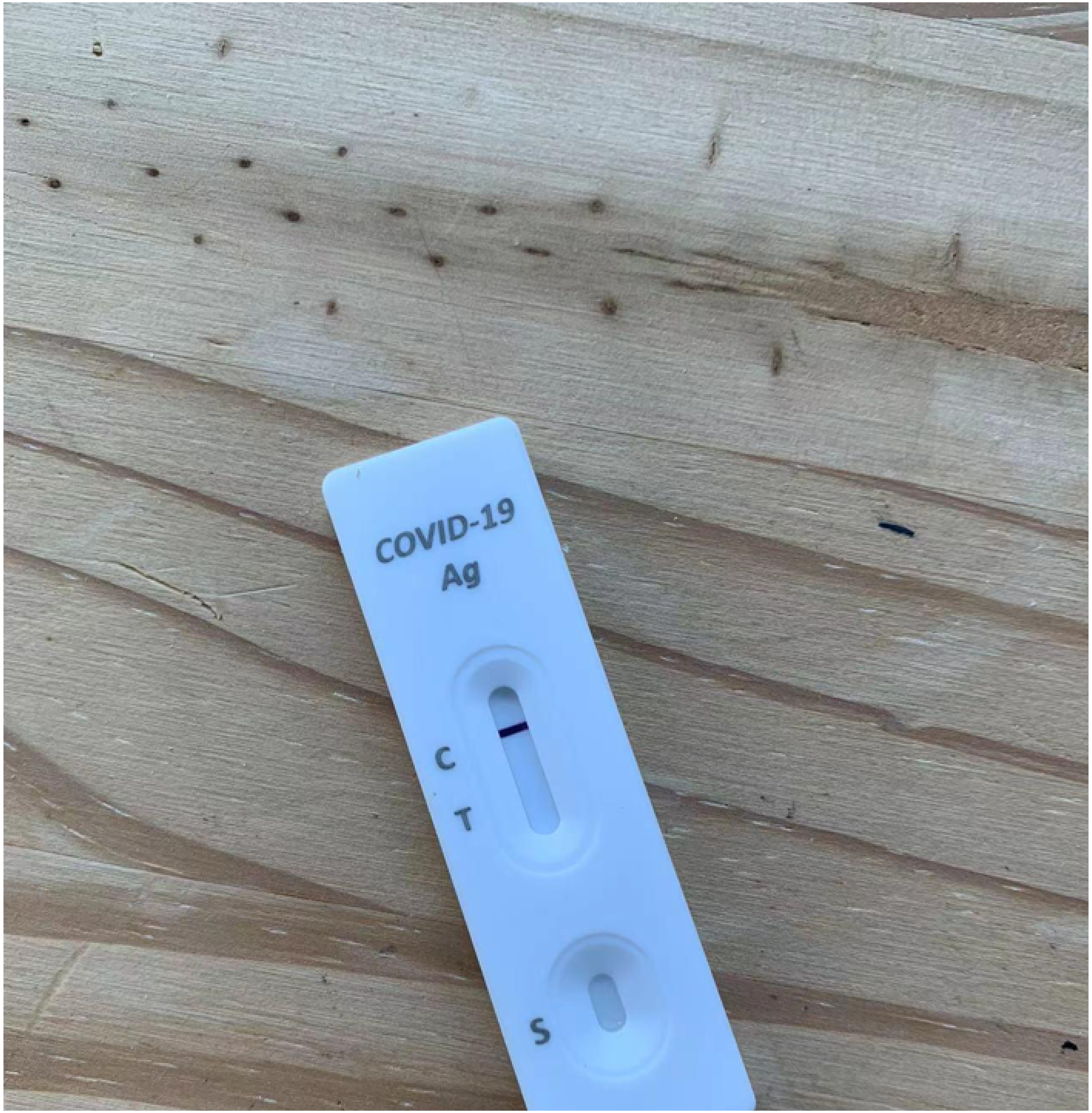
COVID-19
Ag
C
T
S

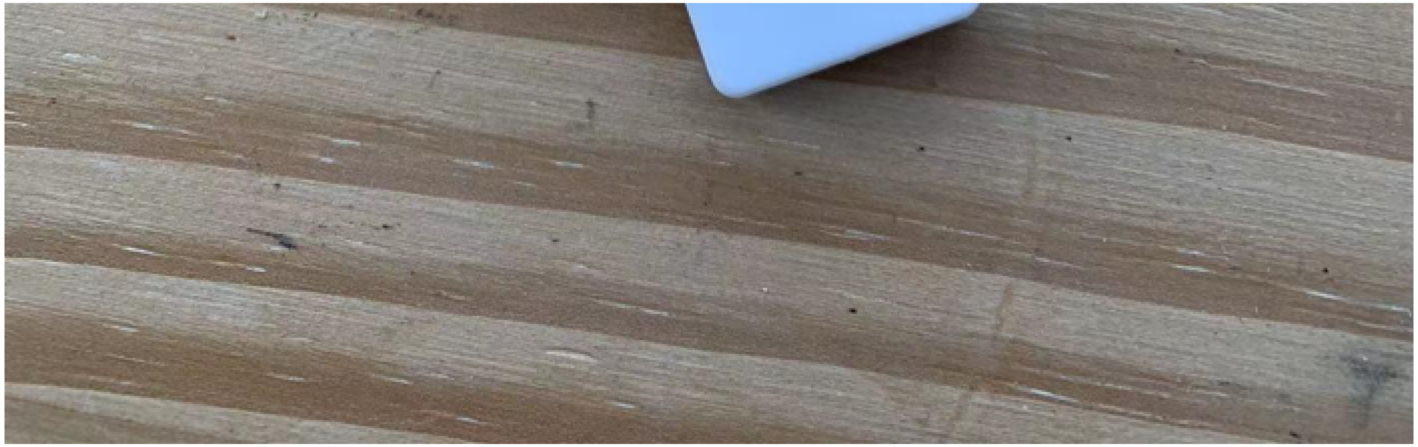

Say they did decide to do a RAT and the result is negative. Based on the information provided, please answer the following questions.

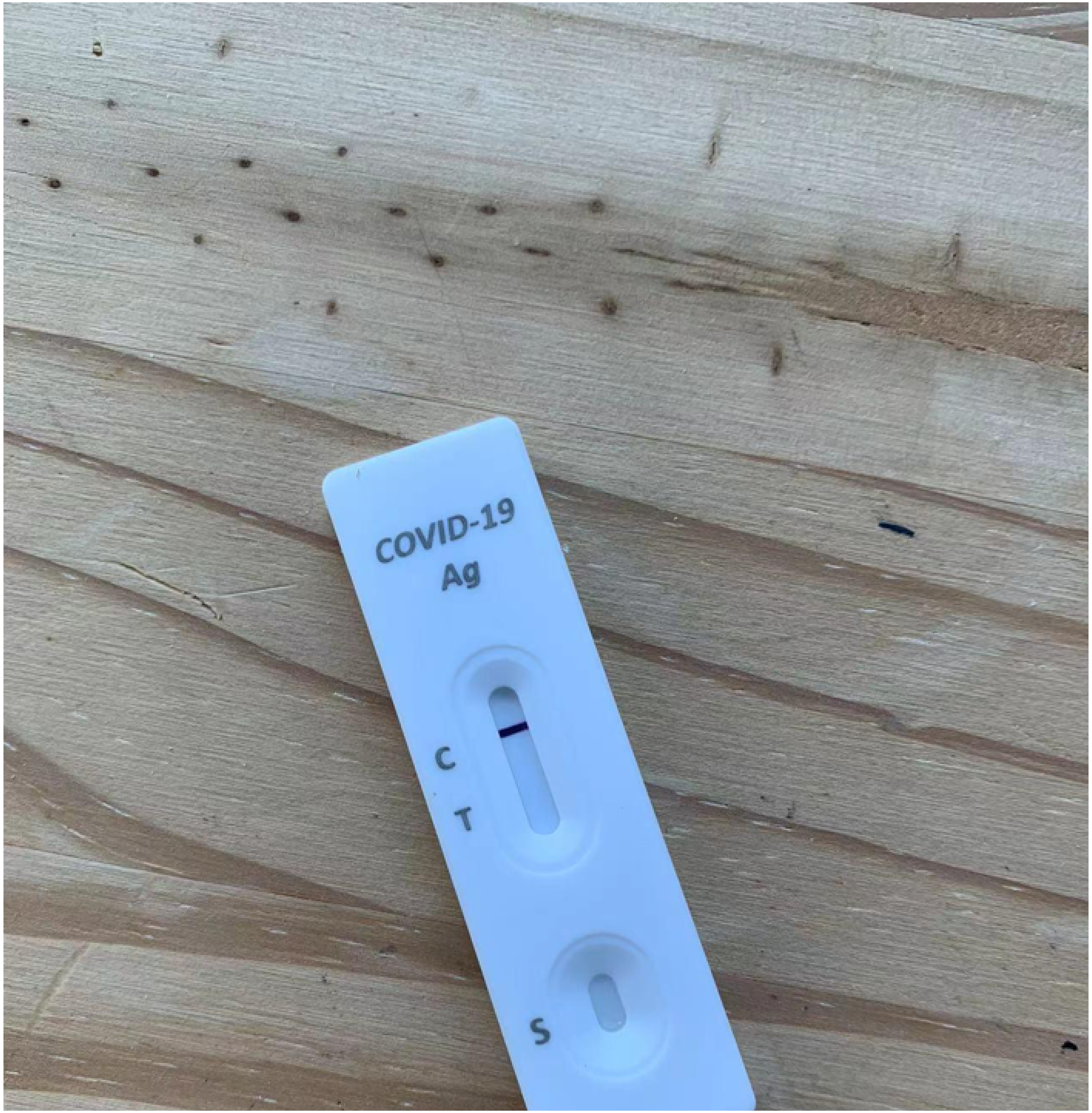
COVID-19
Ag
C
T
S

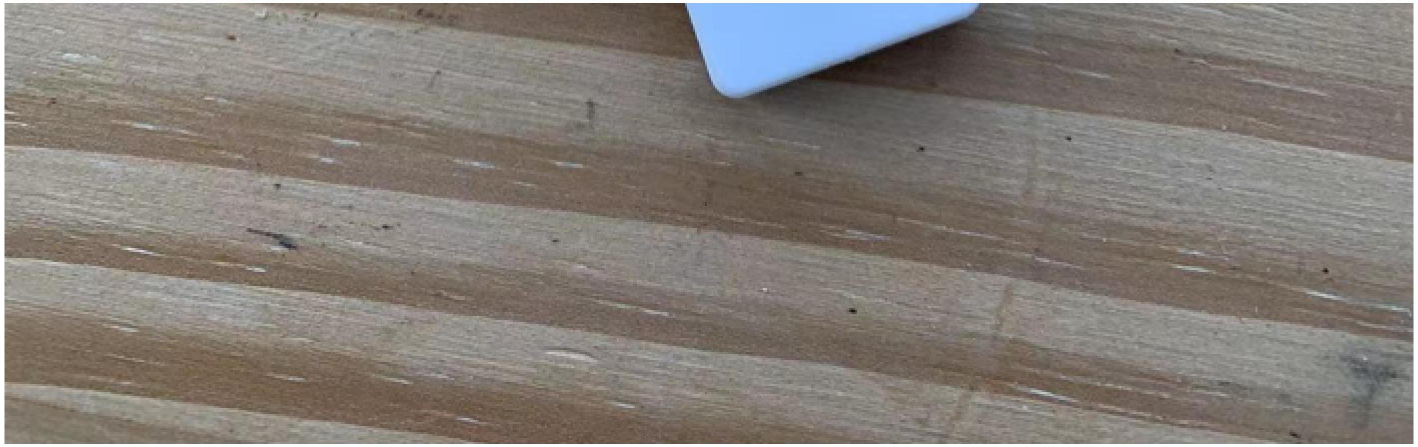

How likely do you think it is that they have COVID-19?

- Very likely
- Likely
- I don’t know
- Unlikely
- Very unlikely

Now we would like you to use numbers to say how likely it is that they have COVID-19 infection.
How likely do you think it is that they have COVID-19 infection? Please drag the slider to a number from 0% (no chance that they are infected) to 100% (they are definitely infected).

	No chance that they are infected		Equal chance of them being infected or not being infected		They are definitely infected
	0	25	50	75	100
Chance of being infectious					

How confident are you in your response above?

	Not confident at all		Medium level of confidence		Absolutely confident
	0	25	50	75	100
Confidence Level					

What advice would you give them to do next, in terms of staying at home?

- ◯ Stay at home without exception
- ◯ Stay at home except for shopping
- ◯ Stay at home except for work
- ◯ Stay at home except for shopping and work
- ◯ Continue to leave the house as normal

What other tests would you advise them to do in the next few days?

- ☐ Have 1 or more PCR tests
- ☐ Do further RAT(s)
- ☐ No further tests

What further actions would you advise them to take (select all that apply)?

- ☐ Avoid visiting people at higher risk of developing complications from COVID-19 (e.g. older people)
- ☐ Avoid crowds
- ☐ Keep 1.5m away from others
- ☐ Wash hands more often
- ☐ Wear a mask indoors when around others

Scenario 5/5:
Imagine it is now six months later and you have dinner at your friend's house with 9 other people. The dinner lasted about 3 hours. 2 days later your friend told you that they and 2 other people at the dinner party have tested positive for COVID-19. You are experiencing a sore throat and runny nose. Would you do a RAT?

- ◯ Yes

◯ No

You take a RAT but the result is negative. Based on the information provided, please answer the following questions.

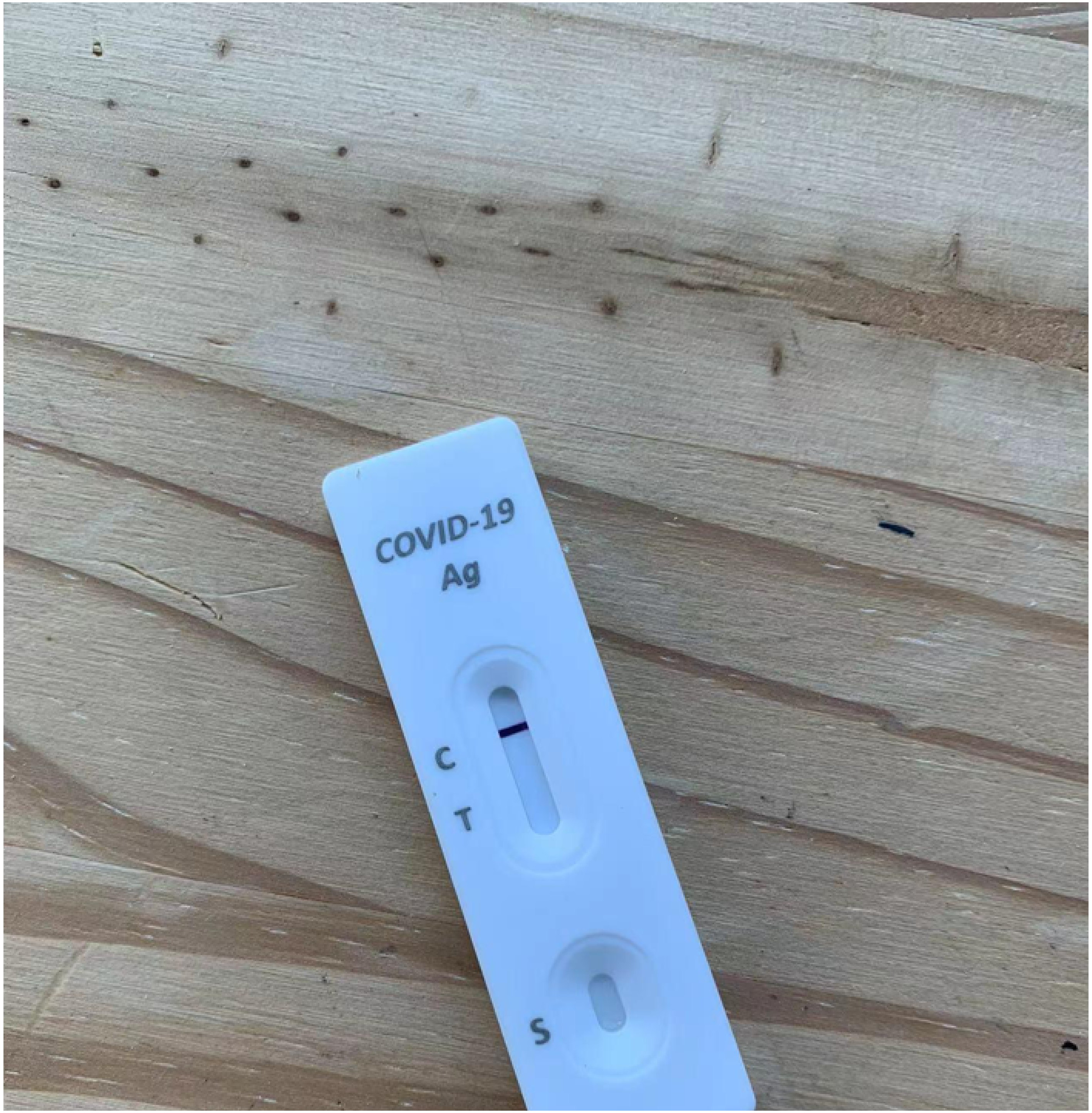
COVID-19
Ag
C
T
S

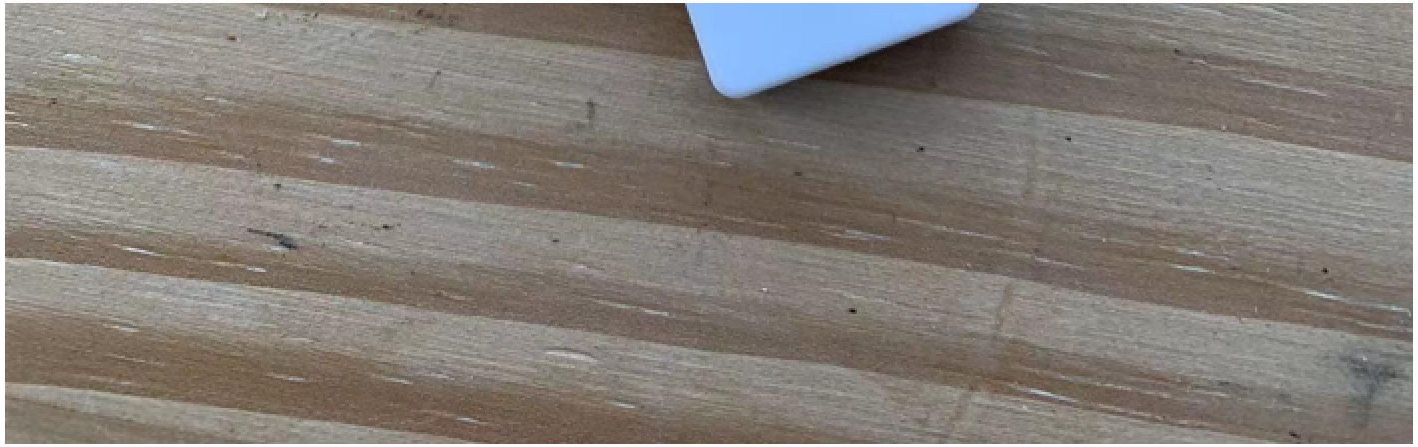

Say you did decide to do a RAT but the result is negative. Based on the information provided, please answer the following questions.

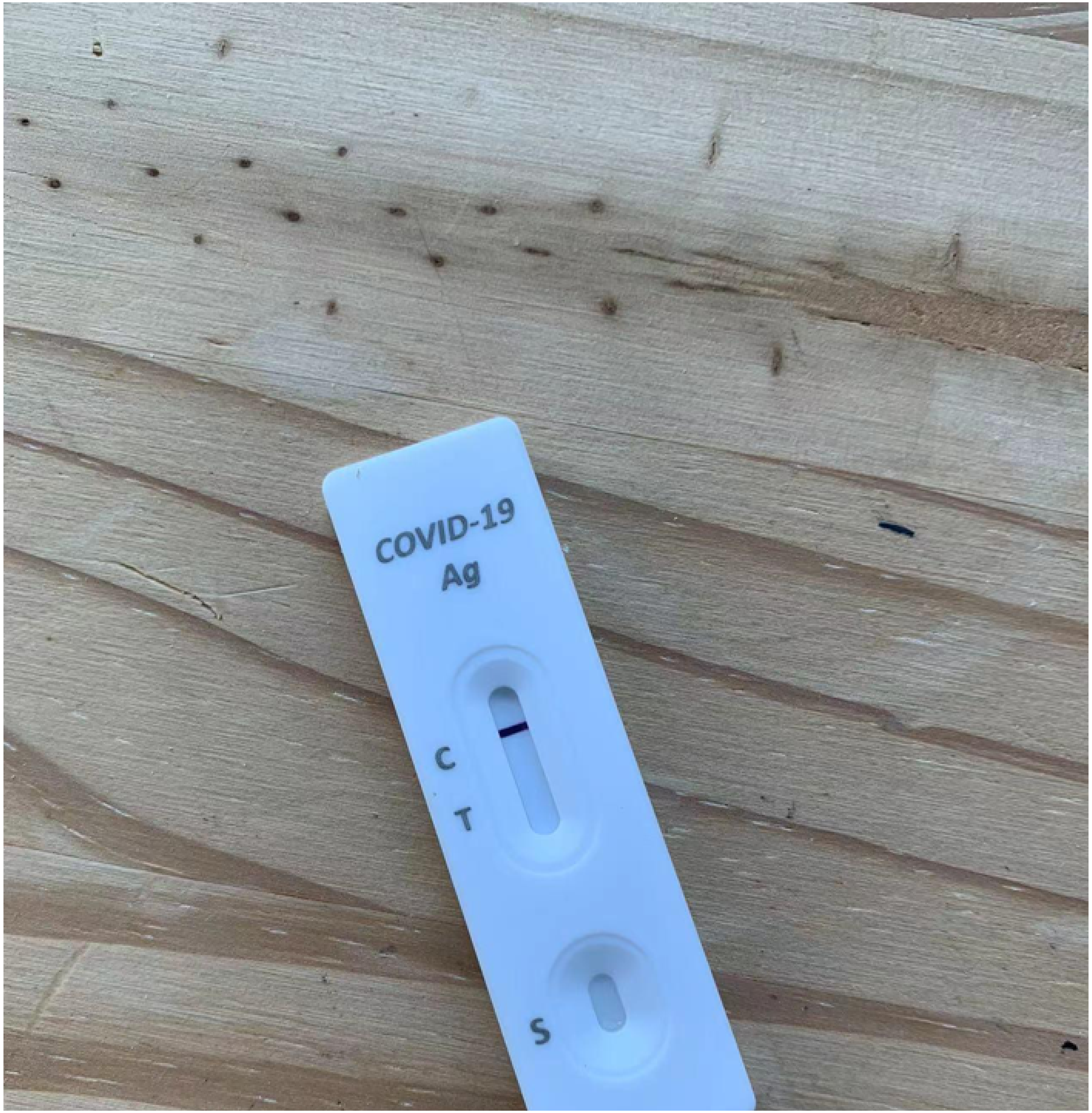
COVID-19
Ag
C
T
S

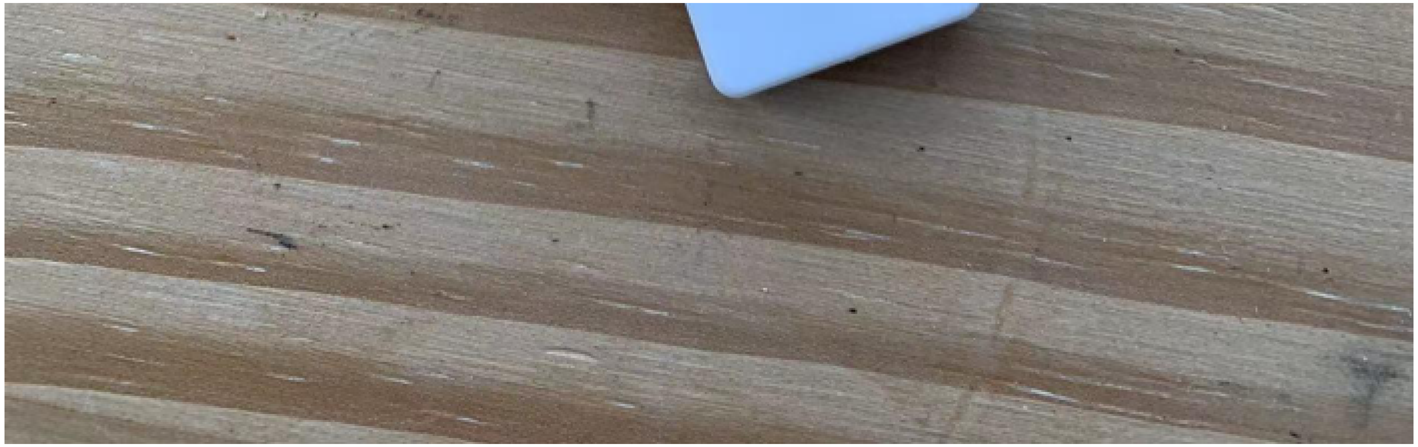

How likely do you think it is that you have COVID-19?

- Very likely
- Likely
- I don’t know
- Unlikely
- Very unlikely

Now we would like you to use numbers to say how likely it is that you have COVID-19 infection.
How likely do you think it is that you have COVID-19 infection? Please drag the slider to a number from 0% (no chance that I am infected) to 100% (I am definitely infected).

	No chance that I am infected		Equal chance of being infected or not being infected		I am definitely infected
	0	25	50	75	100
Chance of being infectious					

How confident are you in your response above?

	Not confident at all		Medium level of confidence		Absolutely confident
	0	25	50	75	100
Confidence Level					

What would you do next, in terms of staying at home?

- ◯ Stay at home without exception
- ◯ Stay at home except for shopping
- ◯ Stay at home except for work
- ◯ Stay at home except for shopping and work
- ◯ Continue to leave the house as normal

If you live with other people, would you:

- ◯ Isolate from other household members
- ◯ Not isolate from other household members
- ◯ ____________ Other
- ◯ Not Applicable (I live alone)

What other tests would you do in the next few days?

- ☐ Have 1 or more PCR tests
- ☐ Do further RAT(s)
- ☐ No further tests

What further actions would you take (select all that apply)?

- ☐ Avoid visiting people at higher risk of developing complications from COVID-19 (e.g. older people)
- ☐ Avoid crowds
- ☐ Keep 1.5m away from others
- ☐ Wash hands more often
- ☐ Wear a mask indoors when around others

Control

A Rapid Antigen Test (RAT) is a quick home test to check if you may have COVID-19 and provides a result within 15 to 20 minutes.

We will present five different scenarios relating to COVID-19. Based on the scenarios, we will require you to answer how likely it is that you (or in one scenario member of your household) has COVID-19 infection, and about specific actions you would take to prevent onward spread of infection.

Scenario 1/5:
Imagine you have been unwell with symptoms including headache, sore throat, fever, runny nose, and loss of taste and smell. Would you do a RAT?

- ◯ Yes
- ◯ No

You do a RAT and the result appears as a strong positive line. Based on the information provided, please answer the following questions.

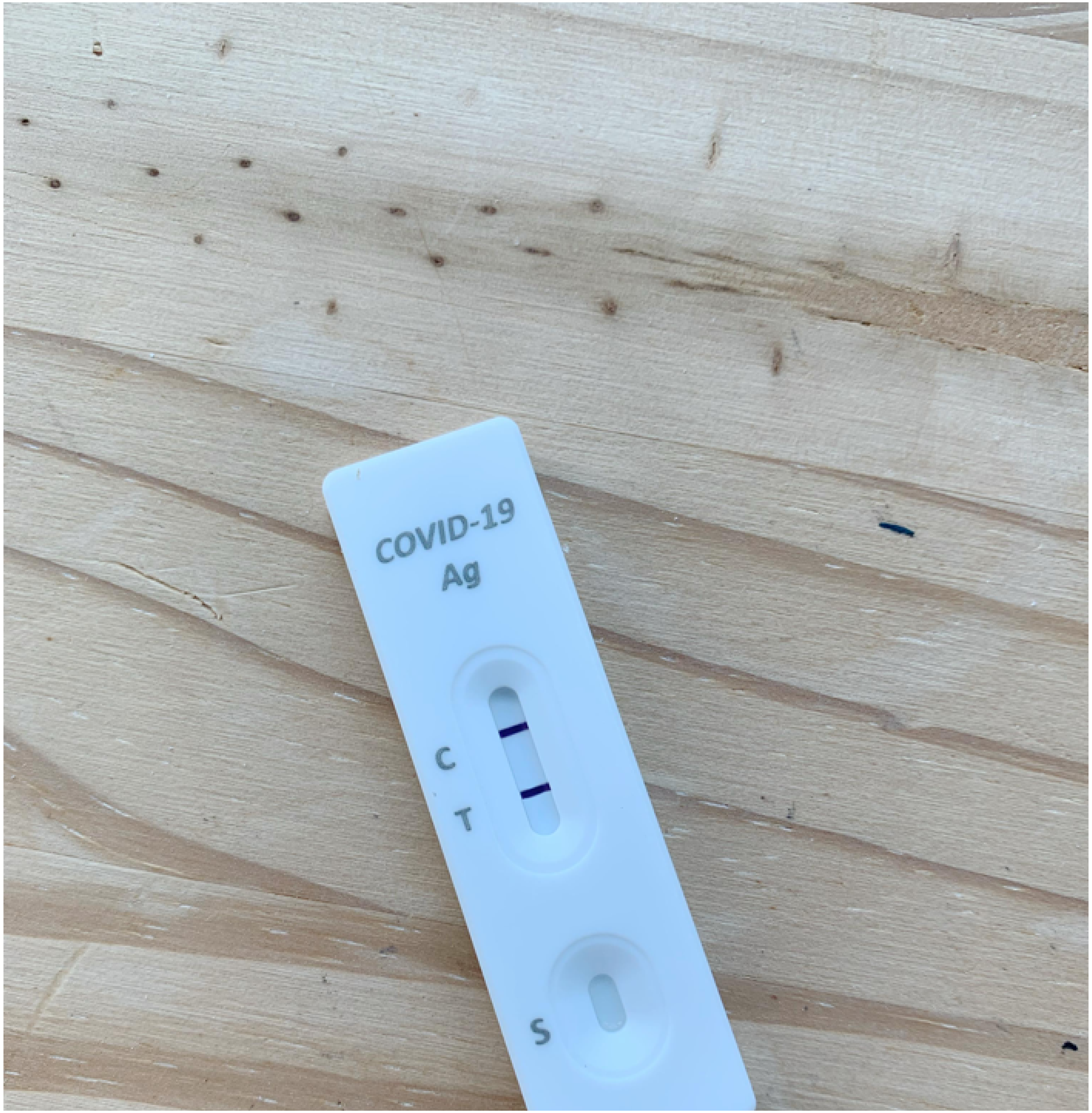
COVID-19
Ag
C
T
S

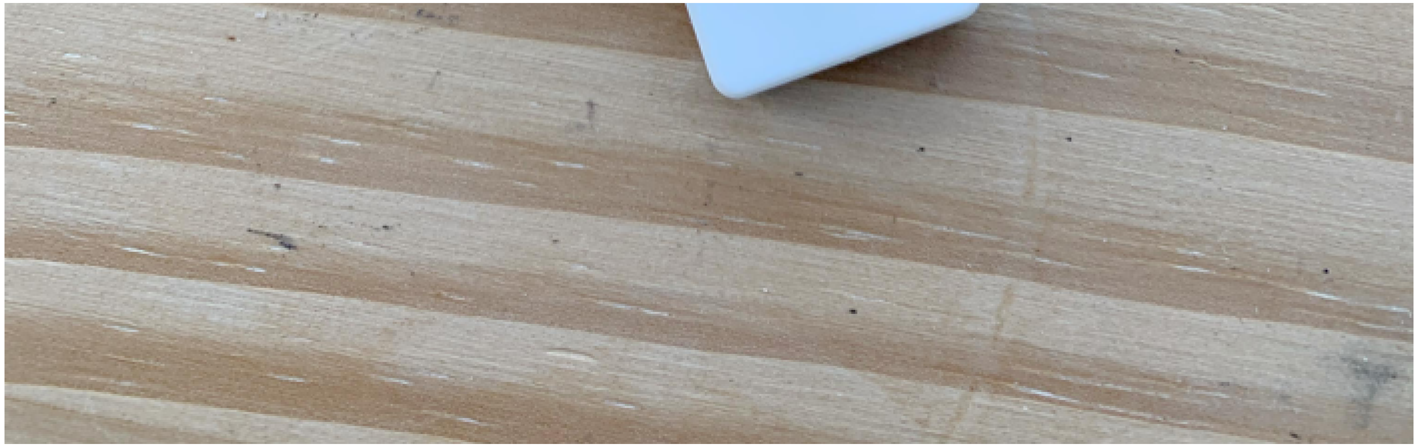

Say you did decide to do a RAT and the result appears as a strong positive line. Based on the information provided, please answer the following questions.

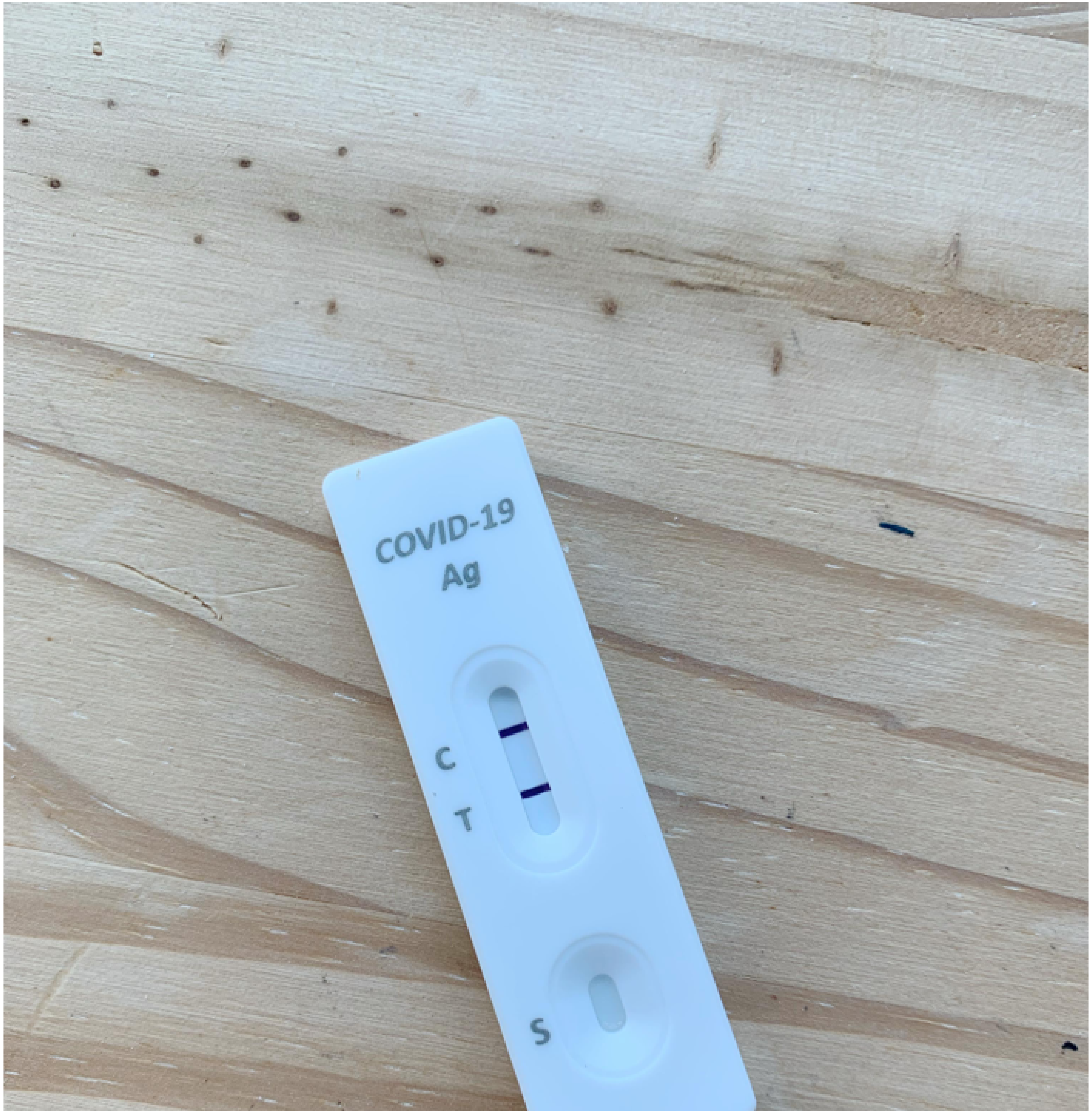
COVID-19
Ag
C
T
S

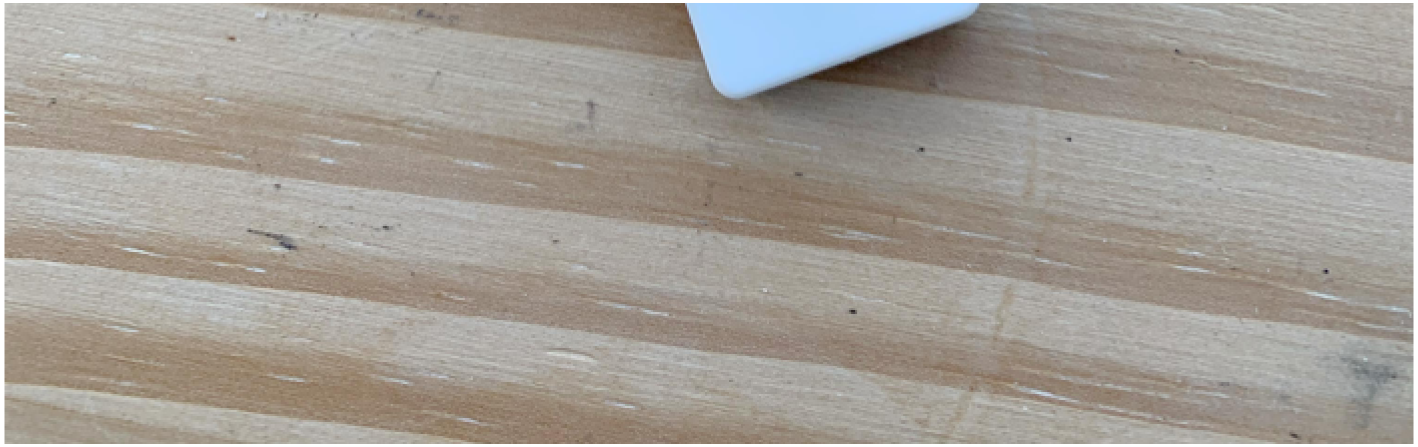

Would you report the results of your positive test?

- ◯ Yes
- ◯ No

What would you do next, in terms of staying at home?

- ◯ Stay at home without exception
- ◯ Stay at home except for shopping
- ◯ Stay at home except for work
- ◯ Stay at home except for shopping and work
- ◯ Continue to leave the house as normal

If you live with other people, would you:

- ◯ Isolate from other household members
- ◯ Not isolate from other household members
- ◯ ____ Other
- ◯ Not Applicable (I live alone)

What other tests would you do in the next few days?

- ☐ Have 1 or more PCR tests
- ☐ Do further RAT(s)
- ☐ No further tests

What further actions would you take (select all that apply)?

- ☐ Avoid visiting people at higher risk of developing complications from COVID-19 (e.g. older people)
- ☐ Avoid crowds
- ☐ Keep 1.5m away from others
- ☐ Wash hands more often
- ☐ Wear a mask indoors when around others

Scenario 2/5:
You have COVID-19 and have been isolating at home. It is now day 4 since you first developed symptoms, and you have felt well since waking up today and no longer have symptoms. Would you do a RAT?

- ◯ Yes
- ◯ No

You do a RAT and the result appears as a strong positive line. Based on the information provided, please answer the following questions.

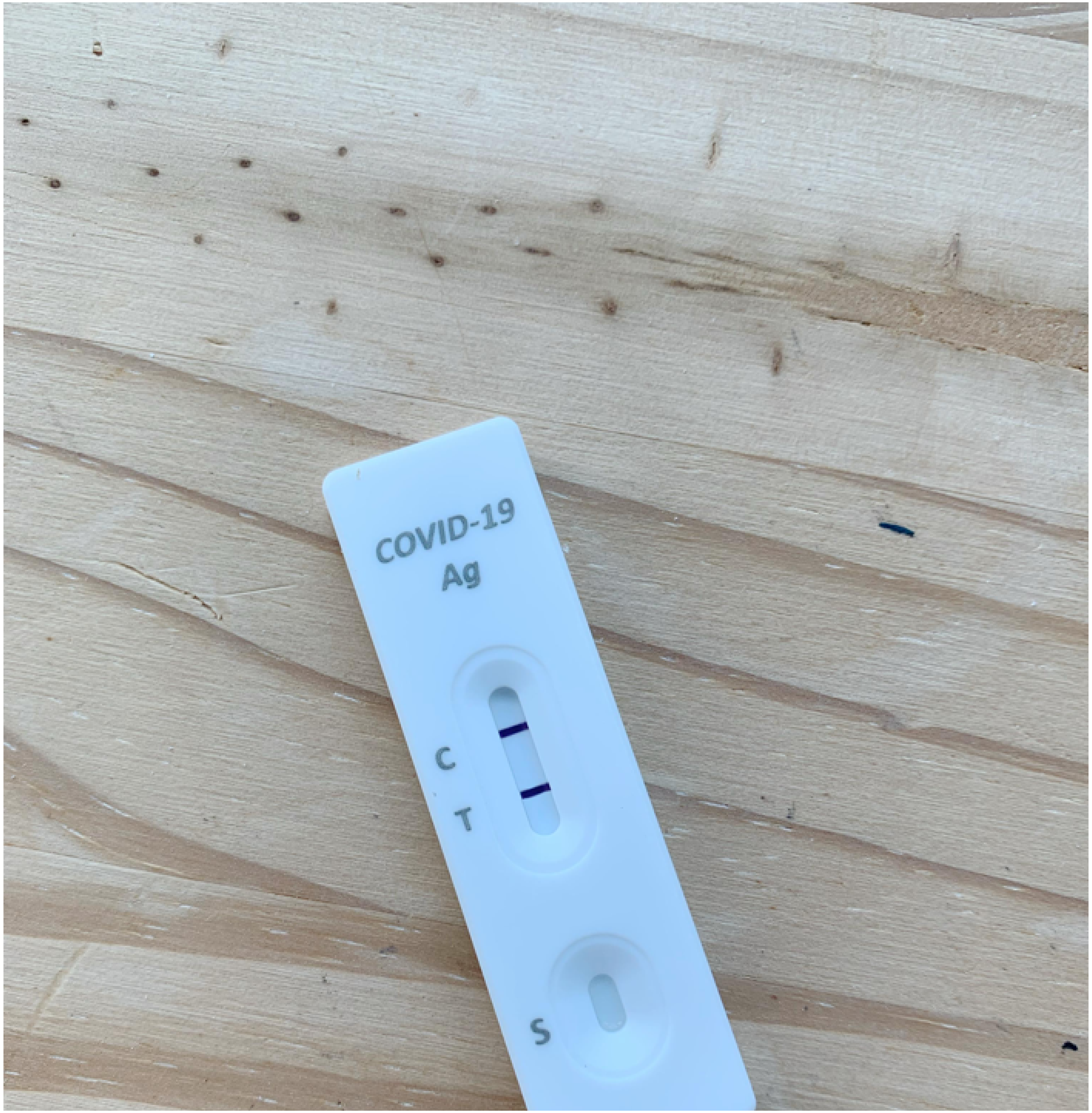
COVID-19
Ag
C
T
S

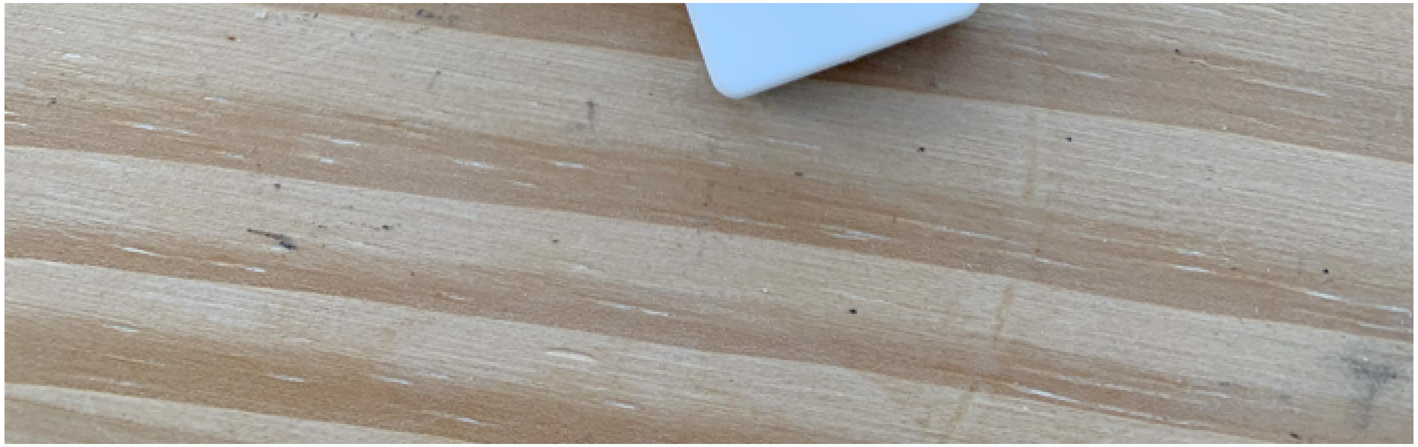

Say you did decide to do a RAT and the result appears as a strong positive line. Based on the information provided, please answer the following questions.

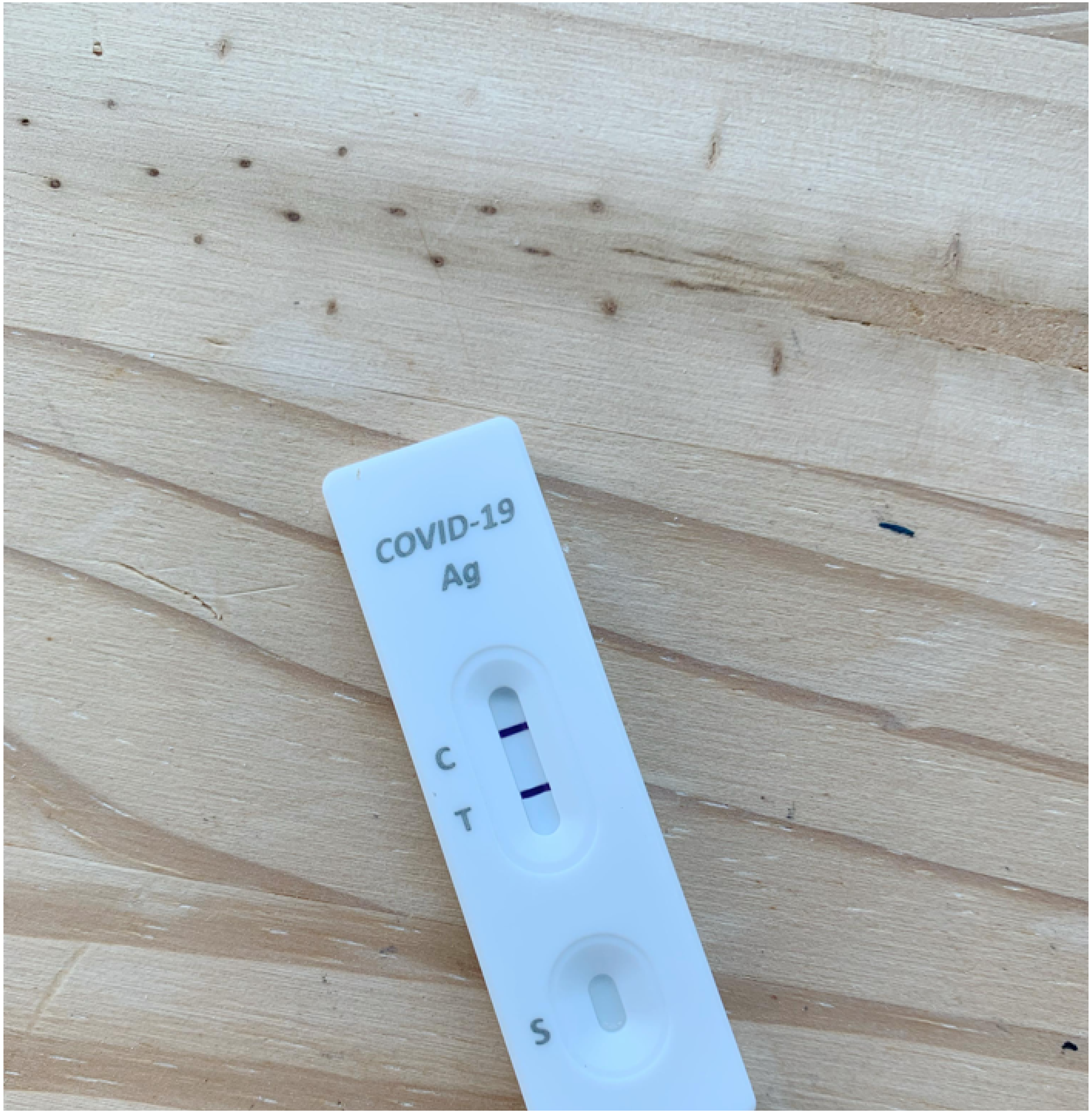
COVID-19
Ag
C
T
S

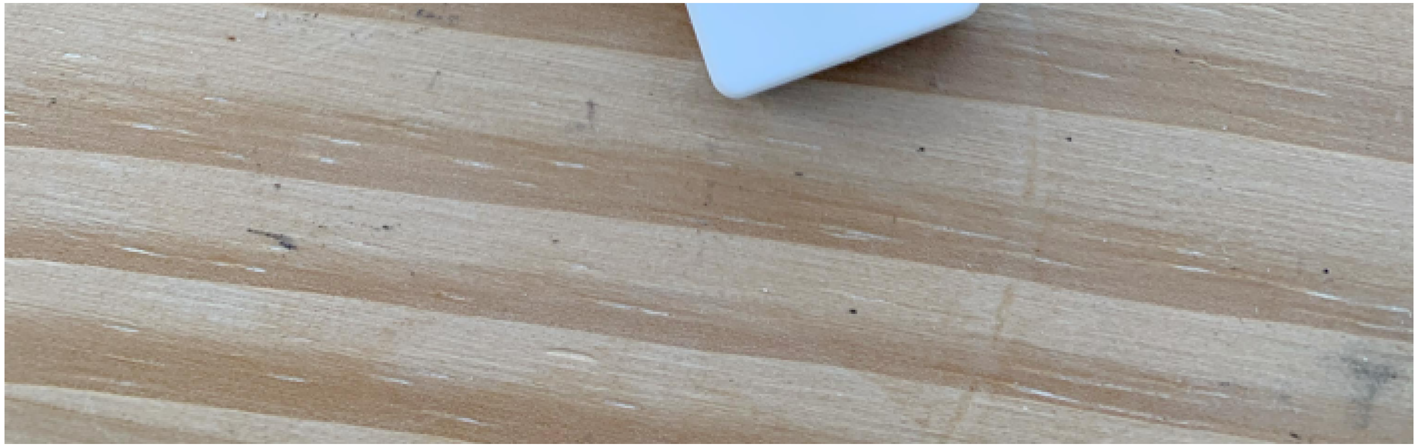

How likely do you think it is that you are still infectious?

- ◯ Very likely I am still infectious
- ◯ Likely I am still infectious
- ◯ I don’t know whether I am still infectious (neither likely nor unlikely)
- ◯ Unlikely I am still infectious
- ◯ Very unlikely I am still infectious

Now we would like you to use numbers to say how likely it is that you are still infectious. How likely do you think it is that you are still infectious? Please drag the slider to a number from 0% (no chance that I am infectious) to 100% (I am definitely infectious).

	No chance that I am infectious		Equal chance of being infectious or not being infectious		I am definitely infectious
	0	25	50	75	100
Chance of being infectious					

How confident are you in your response above?

	Not confident at all		Medium level of confidence		Absolutely confident
	0	25	50	75	100
Confidence Level					

What would you do next, in terms of staying at home for the next 24 hours?

- Stay at home without exception
- Stay at home except for shopping
- Stay at home except for work
- Stay at home except for shopping and work
- Stop self-isolating and leave your house

If you live with other people, would you:

- ◯ Isolate from other household members
- ◯ Not isolate from other household members
- ◯ ______ Other
- ◯ Not Applicable (I live alone)

What other tests would you do in the next few days?

- ☐ Have 1 or more PCR tests
- ☐ Do further RAT(s) until negative
- ☐ No further tests

What further actions would you take (select all that apply)?

- ☐ Avoid visiting people at higher risk of developing complications from COVID-19 (e.g. older people)
- ☐ Avoid crowds
- ☐ Keep 1.5m away from others
- ☐ Wash hands more often
- ☐ Wear a mask indoors when around others

Scenario 3/5:
It is now Day 6 since you first developed symptoms. You have felt well since Day 4, with no symptoms. Would you do a RAT?

- ◯ Yes
- ◯ No

You do a RAT and the result appears as a faint line. Based on the information provided, please answer the following questions.

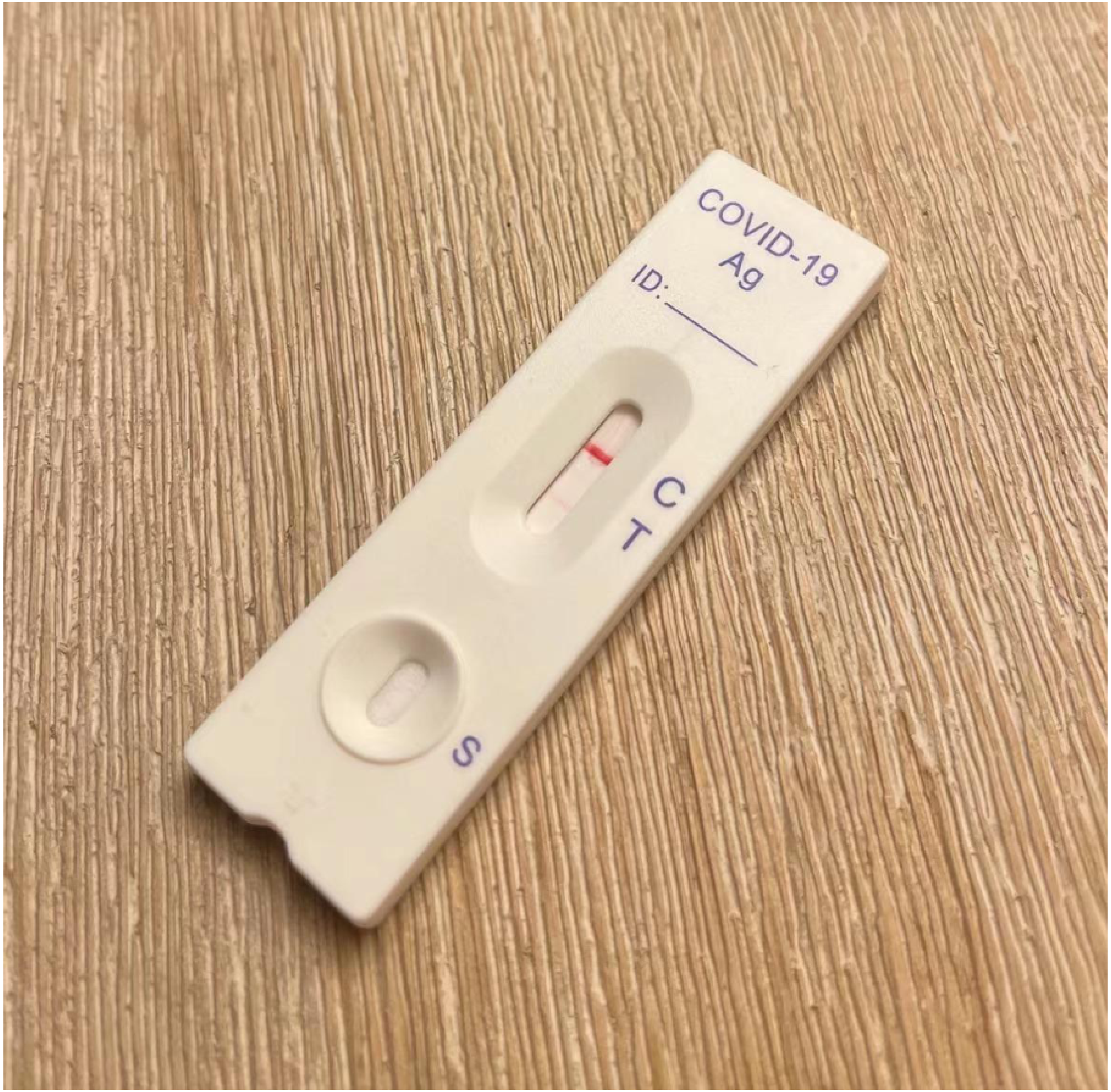
COVID-19
Ag
ID:
C
T
S

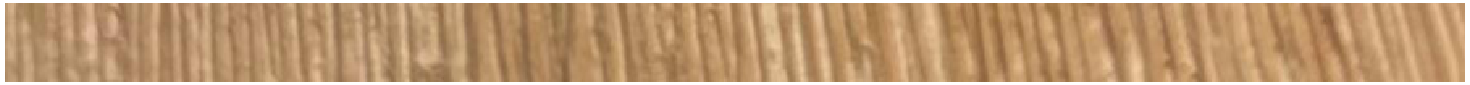

Say you did decide to do a RAT and the result appears as a faint line. Based on the information provided, please answer the following questions.

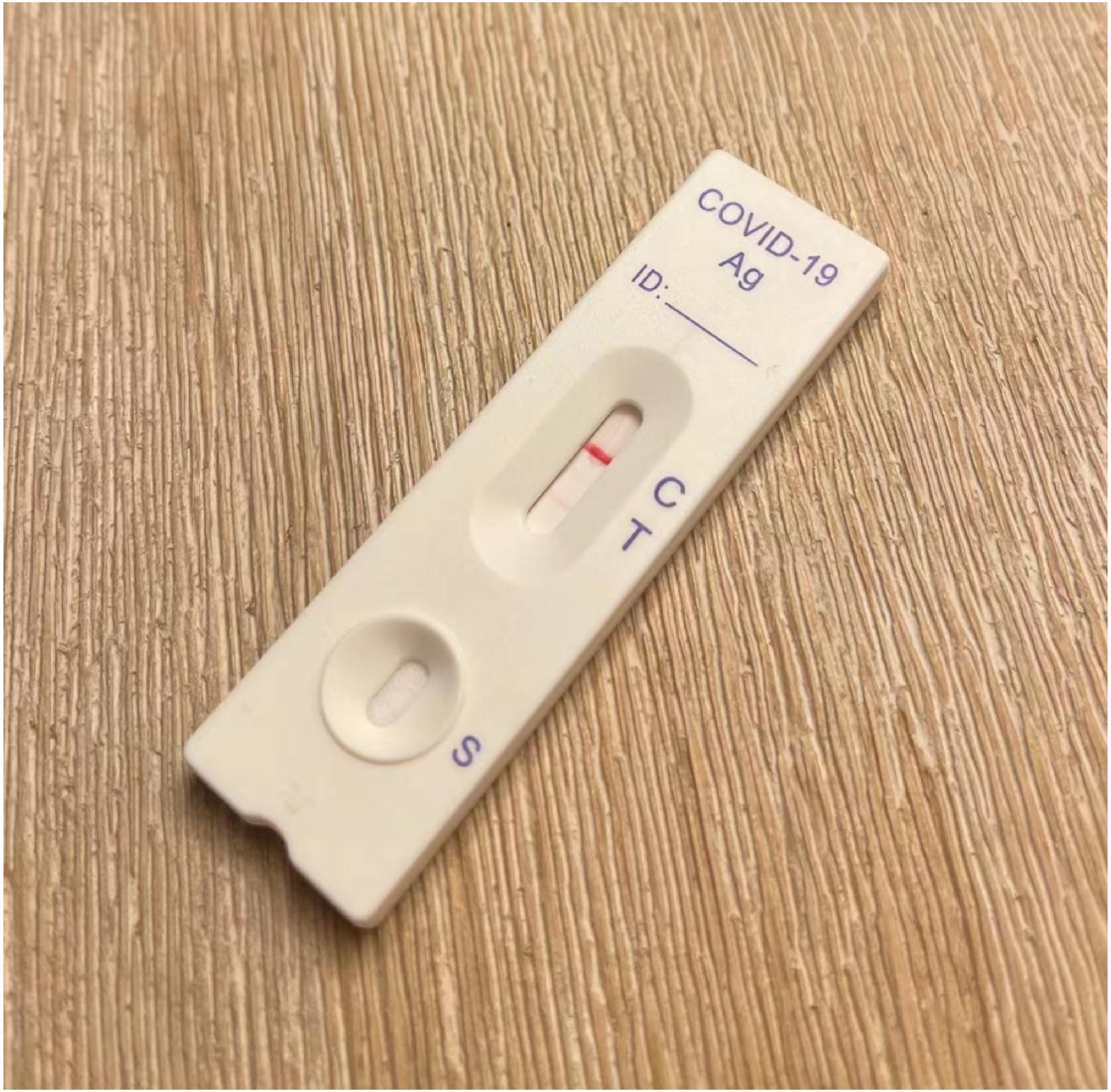
COVID-19
Ag
ID:
C
T
S

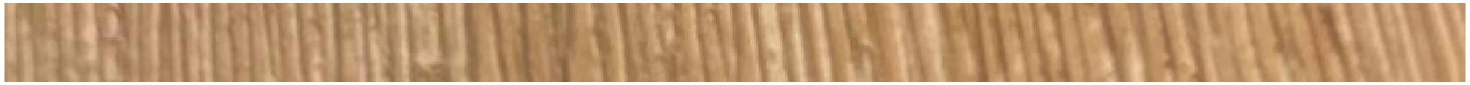

How likely do you think it is that you are still infectious?

- ◯ Very likely I am still infectious
- ◯ Likely I am still infectious
- ◯ I don’t know whether I am still infectious (neither likely nor unlikely)
- ◯ Unlikely I am still infectious
- ◯ Very unlikely I am still infectious

Now we would like you to use numbers to say how likely it is that you are still infectious. How likely do you think it is that you are still infectious? Please drag the slider to a number from 0% (no chance that I am infectious) to 100% (I am definitely infectious).

	No chance that I am infectious		Equal chance of being infectious or not being infectious		I am definitely infectious
	0	25	50	75	100
Chance of being infectious					

How confident are you in your response above?

	Not confident at all		Medium level of confidence		Absolutely confident
	0	25	50	75	100
Confidence Level					

What would you do next, in terms of staying at home?

- ◯ Stay at home without exception
- ◯ Stay at home except for shopping
- ◯ Stay at home except for work
- ◯ Stay at home except for shopping and work
- ◯ Continue to leave the house as normal

If you live with other people, would you:

- ◯ Isolate from other household members
- ◯ Not isolate from other household members
- ◯ [ ] Other
- ◯ Not Applicable (I live alone)

What other tests would you do in the next few days?

- ☐ Have 1 or more PCR tests
- ☐ Do further RAT(s) until negative
- ☐ No further tests

What further actions would you take (select all that apply)?

- ☐ Avoid visiting people at higher risk of developing complications from COVID-19 (e.g. older people)
- ☐ Avoid crowds
- ☐ Keep 1.5m away from others
- ☐ Wash hands more often
- ☐ Wear a mask indoors when around others

Scenario 4/5:
Now imagine someone was staying at your home while you were sick with COVID-19. On Day 7 since you first developed symptoms, they wake up with a sore throat and runny nose. Would you advise them to do a RAT?

- ◯ Yes
- ◯ No

They follow your advice, do a RAT and the result is negative. Based on the information provided, please answer the following questions.

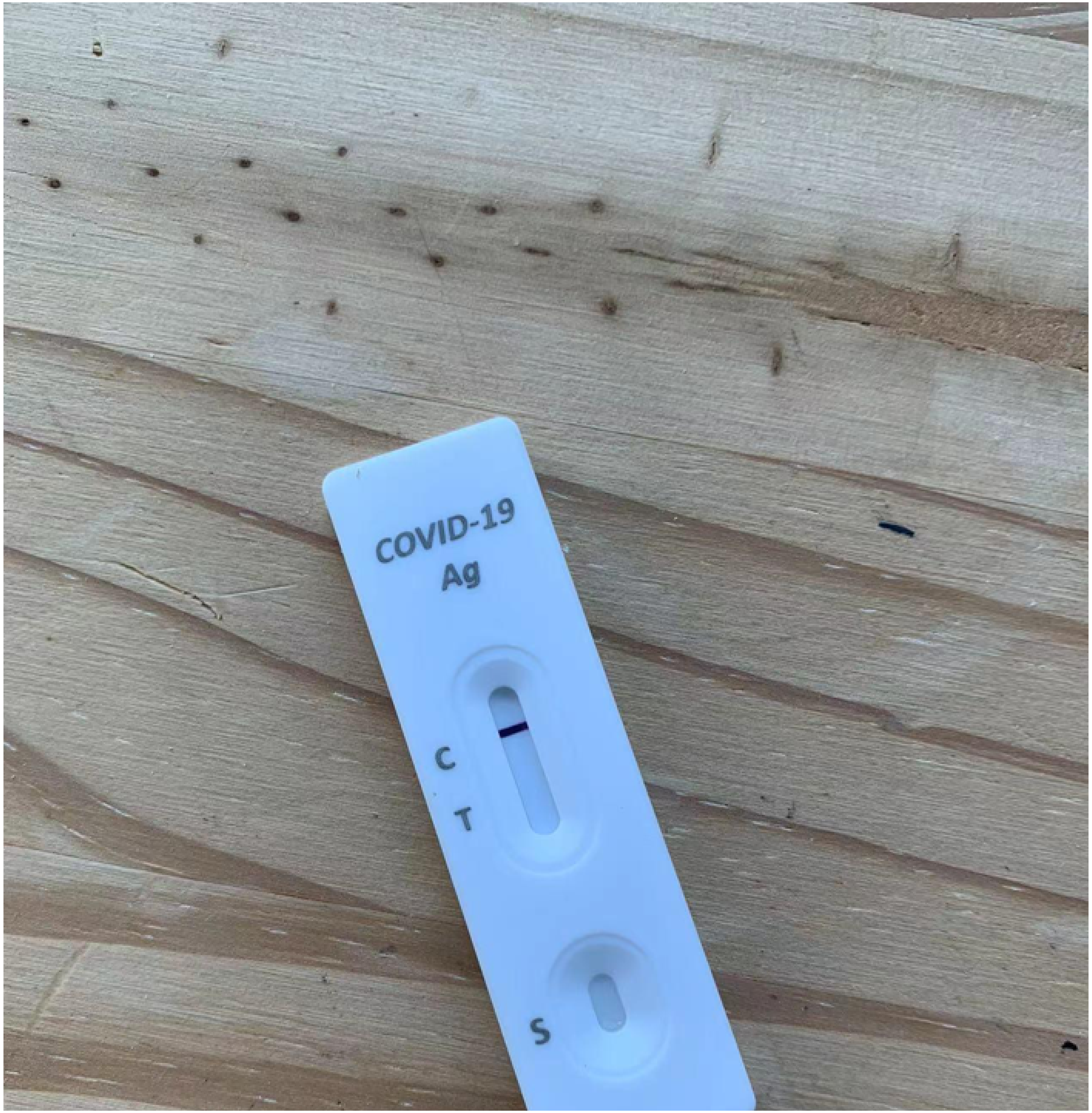
COVID-19
Ag
C
T
S

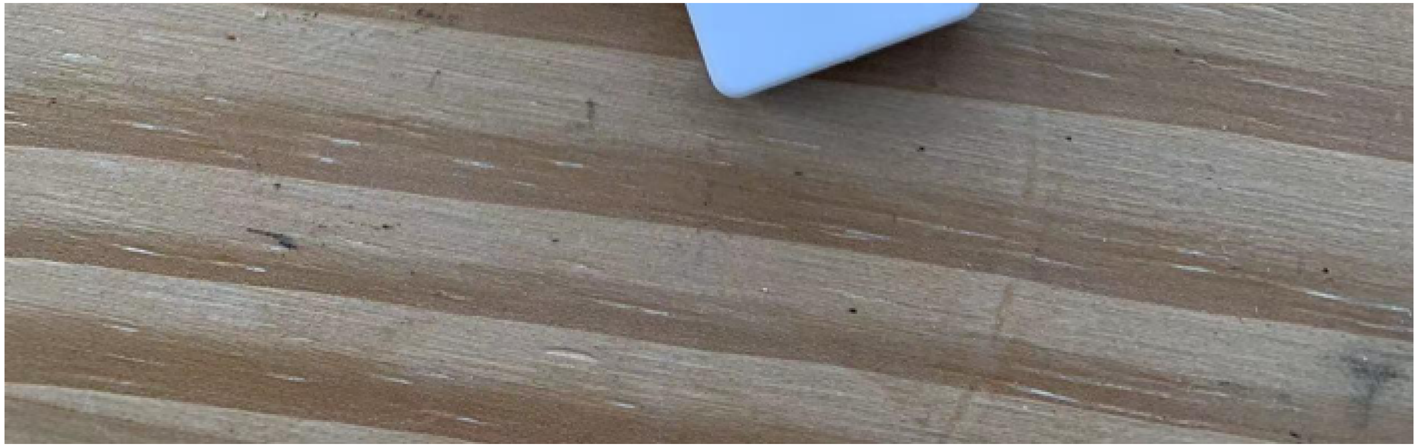

Say they did decide to do a RAT and the result is negative. Based on the information provided, please answer the following questions.

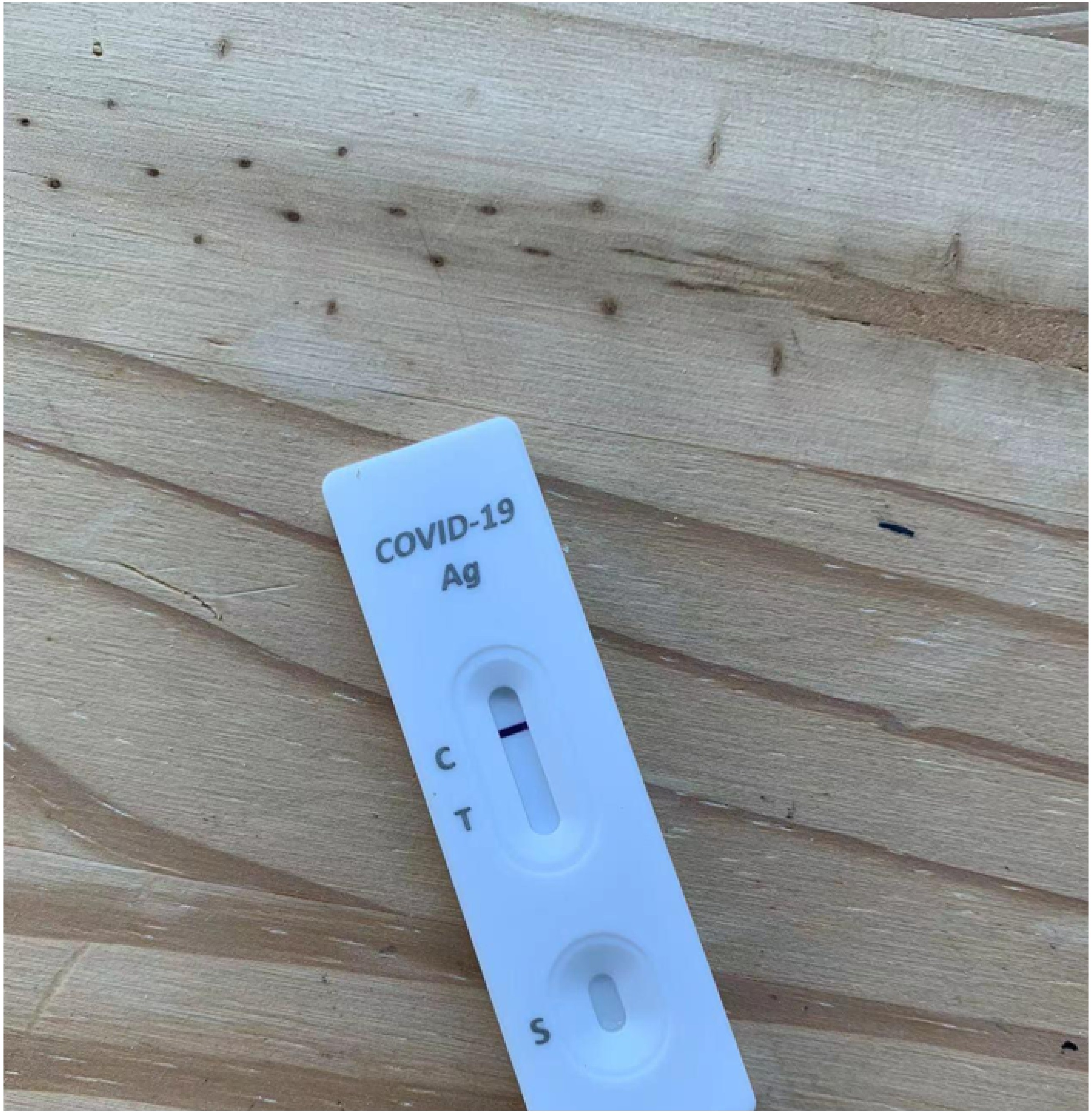
COVID-19
Ag
C
T
S

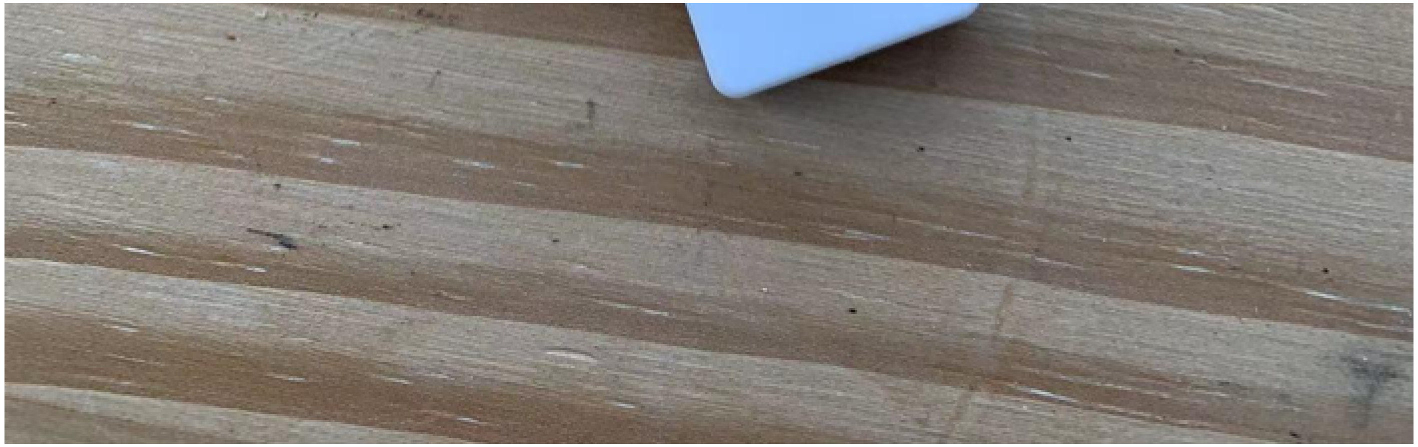

How likely do you think it is that they have COVID-19?

- Very likely
- Likely
- I don’t know
- Unlikely
- Very unlikely

Now we would like you to use numbers to say how likely it is that they have COVID-19 infection.
How likely do you think it is that they have COVID-19 infection? Please drag the slider to a number from 0% (no chance that they are infected) to 100% (they are definitely infected).

	No chance that they are infected		Equal chance of them being infected or not being infected		They are definitely infected
	0	25	50	75	100
Chance of being infectious					

How confident are you in your response above?

	Not confident at all		Medium level of confidence		Absolutely confident
	0	25	50	75	100
Confidence Level					

What advice would you give them to do next, in terms of staying at home?

- ◯ Stay at home without exception
- ◯ Stay at home except for shopping
- ◯ Stay at home except for work
- ◯ Stay at home except for shopping and work
- ◯ Continue to leave the house as normal

What other tests would you do in the next few days?

- ☐ Have 1 or more PCR tests
- ☐ Do further RAT(s)
- ☐ No further tests

What further actions would you advise them to take (select all that apply)?

- ☐ Avoid visiting people at higher risk of developing complications from COVID-19 (e.g. older people)
- ☐ Avoid crowds
- ☐ Keep 1.5m away from others
- ☐ Wash hands more often
- ☐ Wear a mask indoors when around others

Scenario 5/5:
Imagine it is now six months later and you have dinner at your friend’s house with 9 other people. The dinner lasted about 3 hours. 2 days later your friend told you that they and 2 other people at the dinner party have tested positive for COVID-19. You are experiencing a sore throat and runny nose. Would you do a RAT?

- ○ Yes

◯ No

You take a RAT but the result is negative. Based on the information provided, please answer the following questions.

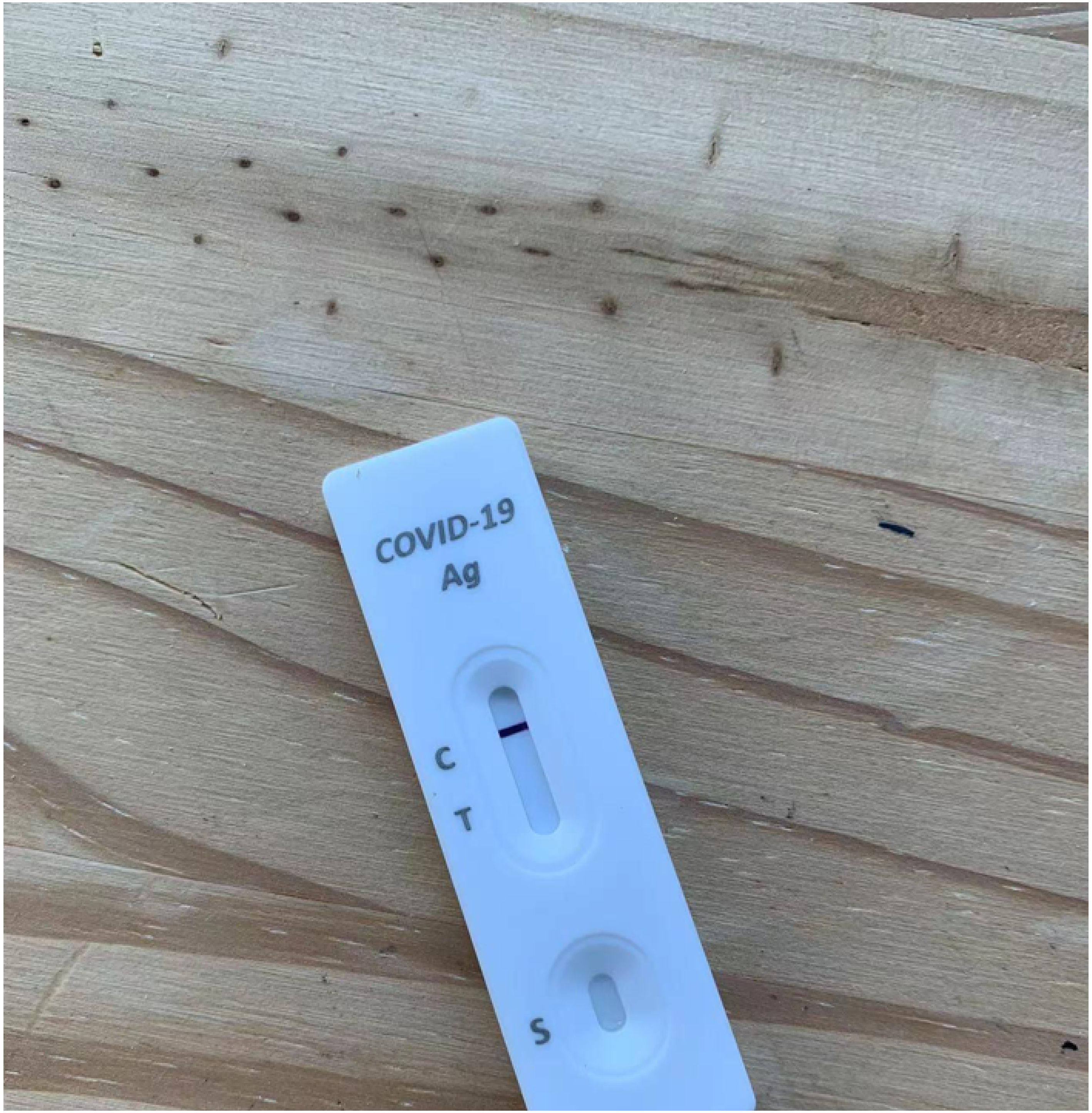
COVID-19
Ag
C
T
S

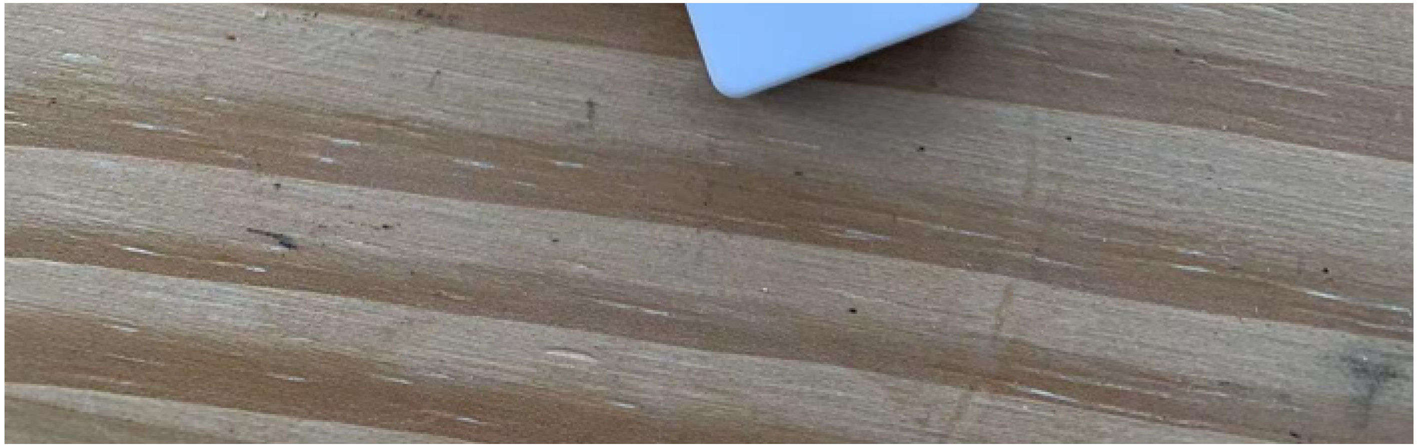

Say you did decide to do a RAT but the result is negative. Based on the information provided, please answer the following questions.

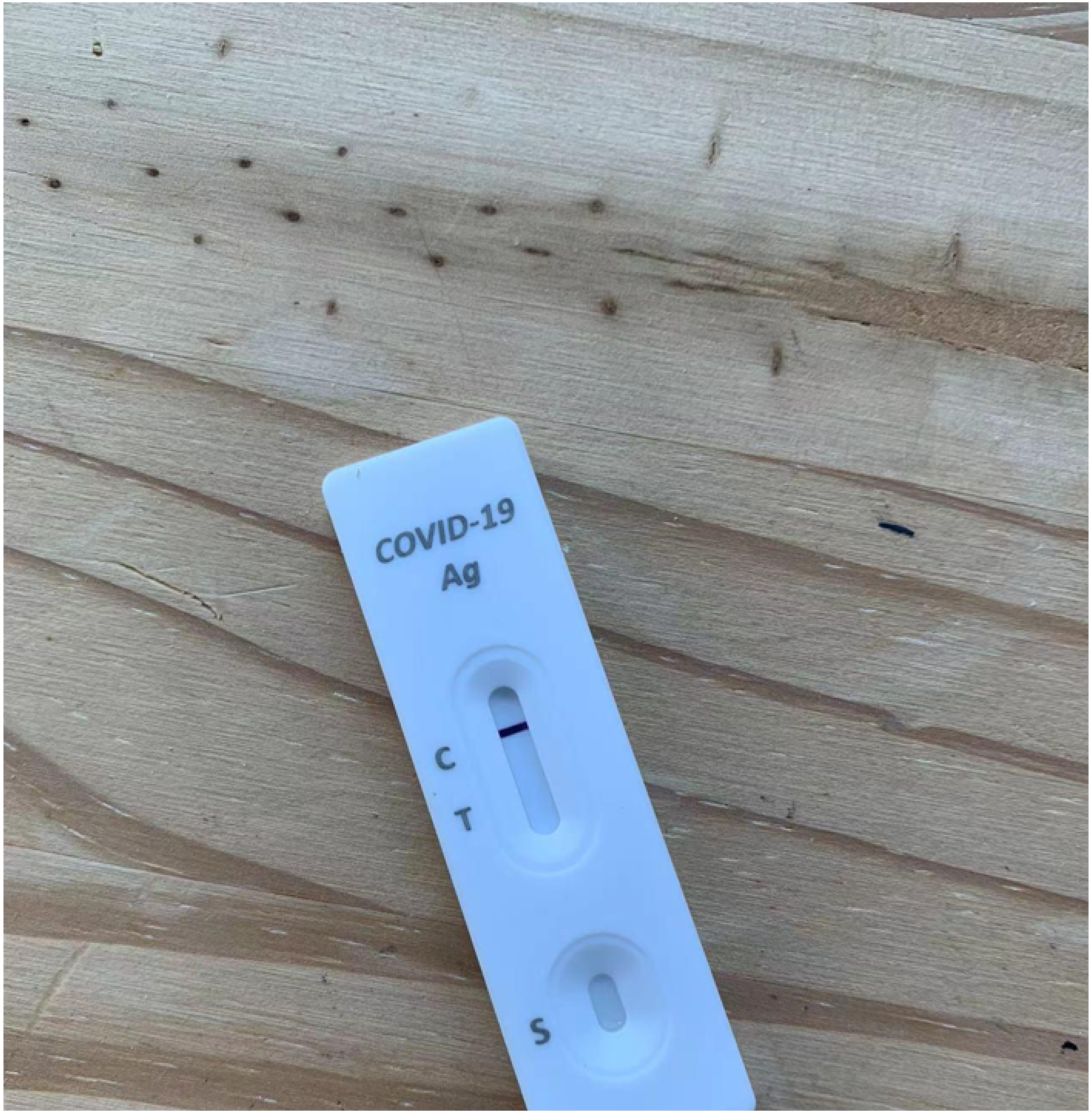
COVID-19
Ag
C
T
S

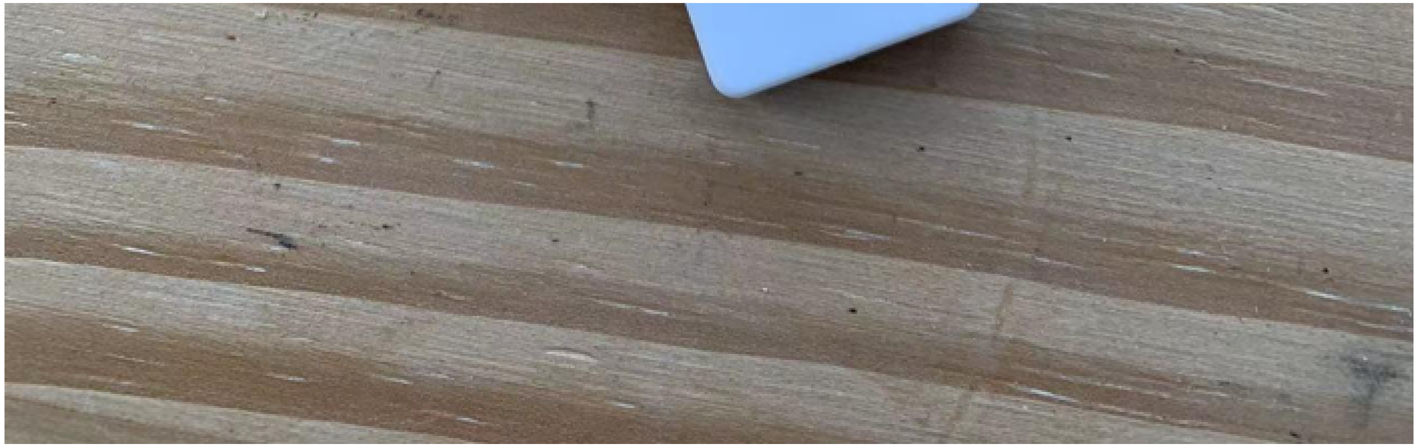

How likely do you think it is that you have COVID-19?

- ◯ Very likely
- ◯ Likely
- ◯ I don’t know
- ◯ Unlikely
- ◯ Very unlikely

Now we would like you to use numbers to say how likely it is that you have COVID-19 infection.
How likely do you think it is that you have COVID-19 infection? Please drag the slider to a number from 0% (no chance that I am infected) to 100% (I am definitely infected).

	No chance that I am infected		Equal chance of being infected or not being infected		I am definitely infected
	0	25	50	75	100
Chance of being infectious					

How confident are you in your response above?

	Not confident at all		Medium level of confidence		Absolutely confident
	0	25	50	75	100
Confidence Level					

What would you do next, in terms of staying at home?

- Stay at home without exception
- Stay at home except for shopping
- Stay at home except for work
- Stay at home except for shopping and work
- Continue to leave the house as normal

If you live with other people, would you:

- ◯ Isolate from other household members
- ◯ Not isolate from other household members
- ◯ [ ] Other
- ◯ Not Applicable (I live alone)

What other tests would you do in the next few days?

- ☐ Have 1 or more PCR tests
- ☐ Do further RAT(s)
- ☐ No further tests

What further actions would you take (select all that apply)?

- ☐ Avoid visiting people at higher risk of developing complications from COVID-19 (e.g. older people)
- ☐ Avoid crowds
- ☐ Keep 1.5m away from others
- ☐ Wash hands more often
- ☐ Wear a mask indoors when around others

Quota Questions and Covariates

We would like to know a bit more about your experiences during COVID-19.

Have you ever tested positive for COVID-19?

- ○ Yes
- ○ No

How many times have you tested positive?

[dropdown]

Provide answers for the most recent time you had COVID-19:

Did you do a Rapid Antigen Test (RAT) test?

- ○ Yes
- ○ No

Was it positive?

- Yes
- No

Did you do a PCR test?

- Yes
- No

Was it positive?

- Yes
- No

Please provide the date (month/year) for each time you had COVID-19.

We are interested in your recent experience of using COVID-19 tests.

Have you had a COVID-19 test in the last month?

- ◯ Yes
- ◯ No

What kind of COVID-19 tests have you used in the last month?

- ☐ Rapid Antigen Test (RAT)
- ☐ PCR

How many Rapid Antigen Tests (RATs) for COVID-19 have you done in the last month?

- ◯ 1-4
- ◯ 4-8
- ◯ >8

Are you required to do RATs for work?

- ◯ Yes
- ◯ No

Have you been vaccinated against COVID-19?

- ◯ Yes, I have had four or more doses
- ◯ Yes, I have had three doses
- ◯ Yes, I have had two doses
- ◯ Yes, I have had one dose
- ◯ No, I have not been vaccinated against COVID-19

Demographic Questions 2

We would like to ask a few more questions about yourself.

Are you living with someone?

- ◯ Yes

○ No

Who are you living with (select all that apply)?

☐ I’m living with adults (18 years or older)
☐ I’m living with children (<18 years old)

How many people live in your household (including you)?

○ 1
○ 2
○ 3
○ 4
○ 5 or more

What state or territory are you currently living in?

○ Australian Capital Territory
○ New South Wales
○ Northern Territory
○ Queensland

- South Australia
- Tasmania
- Victoria
- Western Australia

What is your postcode?

Were you born in Australia?

- Yes
- No

Which country were you born in?

Which year did you arrive in Australia?

What is the main language you speak at home?

- English
- Other

Are you of Aboriginal and/or Torres Strait Islander origin?

- Aboriginal
- Torres Strait Islander
- Both Aboriginal and Torres Strait Islander
- Neither Aboriginal or Torres Strait Islander
- Prefer not to say

How confident are you with filling out medical forms by yourself?

- Not at all
- A little bit
- Somewhat
- Quite a bit

◯ Extremely

Are you a full-time, part-time or a casual worker?

◯ Full-time
◯ Part-time
◯ Casual
◯ N/A

What industry do you work in?

[dropdown]

Does your employer support you to work from home if you are sick?

◯ Yes
◯ No

Thank you note

Thank you very much for taking part in this important research. We really appreciate your time.

Powered by Qualtrics
